# Genome-wide association mapping of gene loci affecting disease resistance in the rice-*Fusarium fujikuroi* pathosystem

**DOI:** 10.1186/s12284-019-0337-3

**Published:** 2019-11-21

**Authors:** Szu-Yu Chen, Ming-Hsin Lai, Chih-Wei Tung, Dong-Hong Wu, Fang-Yu Chang, Tsung-Chun Lin, Chia-Lin Chung

**Affiliations:** 1 Department of Plant Pathology and Microbiology, National Taiwan University, No. 1, Sec. 4, Roosevelt Rd., Taipei City, 10617 Taiwan; 2 Crop Science Division, Taiwan Agricultural Research Institute, No. 189, Zhongzheng Rd., Wufeng Dist., Taichung City, 41362 Taiwan; 3 Department of Agronomy, National Taiwan University, No. 1, Sec. 4, Roosevelt Rd., Taipei City, 10617 Taiwan; 4 Kaohsiung District Agricultural Research and Extension Station, No.2-6, Dehe Rd., Changzhi Township, Pingtung County, 90846 Taiwan; 5 Plant Pathology Division, Taiwan Agricultural Research Institute, No. 189, Zhongzheng Rd., Wufeng Dist., Taichung City, 41362 Taiwan

**Keywords:** *Fusarium fujikuroi*, Genome-wide association mapping, Rice diversity panel 1

## Abstract

**Background:**

Rice bakanae disease has emerged as a new threat to rice production. In recent years, an increase in the occurrence and severity of bakanae disease has been reported in several areas in Asia. Although bakanae disease affects rice yield and quality, little is known about the genetics of bakanae resistance in rice. The lack of large-scale screens for bakanae resistance in rice germplasm has also limited the development and deployment of resistant varieties.

**Results:**

A genome-wide association study (GWAS) was conducted to identify genes/loci conferring bakanae resistance in rice. A total of 231 diverse accessions from Rice Diversity Panel 1 (RDP1) were inoculated with a highly virulent Taiwanese *Fusarium fujikuroi* isolate and assessed for resistance using two parameters: (1) disease severity index based on visual rating and (2) colonization rate determined by reisolation of *F. fujikuroi* from the basal stems of infected rice seedlings. We identified 14 quantitative trait loci (QTLs) (10 for disease severity and 4 for colonization rate), including 1 mapped for both parameters. A total of 206 candidate genes were identified within the 14 QTLs, including genes encoding leucine-rich repeat (LRR)-containing and NB-ARC (nucleotide-binding adaptor shared by APAF-1, R proteins, and CED-4) proteins, hormone-related genes, transcription factor genes, ubiquitination-related genes, and oxidase/oxidoreductase genes. In addition, a candidate QTL (*qBK1.7*) that co-localized with the previously identified QTLs *qBK1* and *qFfR1*, was verified by linkage analysis using a population of 132 recombinant inbred lines derived from IR64 x Nipponbare. GWAS delineated *qBK1.7* to a region of 8239 bp containing three genes. Full-length sequencing across *qBK1.7* in 20 rice accessions revealed that the coding regions of two LRR-containing genes *Os01g0601625* and *Os01g0601675* may be associated with bakanae resistance.

**Conclusions:**

This study facilitates the exploitation of bakanae resistance resources in RDP1. The information on the resistance performance of 231 rice accessions and 14 candidate QTLs will aid efforts to breed resistance to bakanae and uncover resistance mechanisms. Quantification of the level of *F. fujikuroi* colonization in addition to the conventional rating of visual symptoms offers new insights into the genetics of bakanae resistance.

## Background

Rice is an important crop for more than half of the people worldwide. Rice bakanae disease caused by *Fusarium fujikuroi* is widely distributed in rice growing areas, causing reduced grain quality and yield loss up to 40% (Takahashi et al. 1991). In recent years, the disease has become a new threat to rice production. Increasing severity of the disease has been reported in many Asian countries such as Pakistan, Bangladesh, northern India, south Korea, and Taiwan (Khan et al. 2000; Chu et al. 2010; Haq et al. 2011; Gupta et al. 2014; Kim et al. 2015). Seed disinfection using fungicides has long been considered an effective method for the control of bakanae disease. However, fungicide-resistant strains have emerged in China, Korea, and Taiwan. Benzimidazole-resistant isolates were found in Jiangsu, China (Chen et al. 2014), prochloraz-resistant isolates were discovered in Korea and Taiwan (Kim et al. 2010; Chen et al. 2016), and a few tebuconazole-resistant isolates were also found in Taiwan (Chen et al. 2016).

*F. fujikuroi* infects rice grains, and the infected rice seedlings can show diverse morphological changes including abnormal elongation of the stem or internodes, development of adventitious roots on the stem, a wider leaf angle, slenderness, and even death. The level of rice resistance to bakanae is difficult to evaluate owing to the complexity of disease symptoms. In most previous studies, resistance was assessed based on mortality rate, disease incidence, or disease severity (Yang et al. 2006; Hur et al. 2015; Fiyaz et al. 2016; Volante et al. 2017; Ji et al. 2017; Lee et al. 2018). In addition to making visual observations, Chen et al. (2015) investigated the colonization of *F. fujikuroi* in eight cultivars by cultivating five consecutive 1-cm-segments cut from the basal stem of infected plants on FFC selective medium (Hsu 2013). Higher re-isolation frequencies were observed from susceptible than resistant cultivars. Carneiro et al. (2017) developed a TaqMan real-time PCR assay to quantify *F. fujikuroi* in rice tissues. Higher biomass of *F. fujikuroi* was detected in three susceptible cultivars than in three resistant cultivars. These lines of evidence suggest that bakanae resistance is associated with restriction of the spread and colonization of *F. fujikuroi*.

Although bakanae disease has been recognized since it was first reported in 1898 (Takahashi et al. 1991), little is known about how rice defends against it. Insufficient information about the resistance of rice varieties to bakanae disease has impeded the development of resistance breeding and disease control. Hsu et al. (2013) and Kim et al. (2014) developed convenient inoculation systems for large-scale screening of bakanae-resistant rice cultivars. A few research teams in China, Korea, India, and Italy conducted quantitative trait locus (QTL) analysis to identify QTLs and putative genes related to bakanae resistance. A total of 11 QTLs located on chromosomes 1, 3, 4 and 10 were identified by linkage mapping using bi-parental populations and a genome-wide association study (GWAS) using a population of 138 diverse *japonic*a accessions (Table 1) (Yang et al. 2006; Hur et al. 2015; Fiyaz et al. 2016; Volante et al. 2017; Ji et al. 2017; Lee et al. 2018, 2019). Among the eight QTLs identified on chromosome 1, three QTLs (*qFfR1*, *qBK1* and *qBK1.1*) mapped in different populations were co-localized in an ~ 1.54-Mb region (22.56–24.10 Mb). Though several QTLs have been identified, the causal genes controlling bakanae disease resistance remain to be verified.

**Table 1:** Bakanae resistance QTLs mapped from previous studies

QTL	Chr.	QTL region (Mb)^a^	PVE (%)^b^	Mapping population	Trait	Publication
*qBK1_628091*	1	0.62–1.04	–	*Japonica* germplasm (138 accessions)	0–4 disease scale	Volante et al. 2017
*qBK1.2*	1	3.10–3.36*	24.74	F_14_ RILs	mortality rate	Fiyaz et al. 2016
*qBK1.3*	1	4.65–8.41*	6.49	F_14_ RILs	mortality rate	Fiyaz et al. 2016
*qBK1*^*WD*^	1	13.54–15.13	20.20	F_2:4_ RILs	proportion of healthy plants	Lee et al. 2018
*qFfR1*	1	22.56–24.10	–	F_2_ and F_3_	mortality rate	Ji et al. 2017
*qBK1*	1	23.21–23.72	65	BC_6_F_4_	proportion of healthy plants	Hur et al. 2015
*qBK1*	1	23.64–23.67	–	BC_7_F_4_	proportion of healthy plants	Lee et al. 2019
*qBK1.1*	1	23.32–23.34*	4.76	F_14_ RILs	mortality rate	Fiyaz et al. 2016
*qB1*	1	34.10–34.95*	13.40	Double haploid	Difference in seedling length	Yang et al. 2006
*qBK3.1*	3	21.43–21.78	9.10	F_14_ RILs	mortality rate	Fiyaz et al. 2016
*qBK4_31750955*	4	31.16–31.75	–	*Japonica *germplasm (138 accessions)	0–4 disease scale	Volante et al. 2017
*qB10*	10	18.72–19.23*	13.30	Double haploid	Difference in seedling length	Yang et al. 2006

^a^The genomic position is based on the MSU7 Nipponbare reference genome

^b^Phenotypic variation explained

*QTL region was estimated using the physical positions of the markers

To help manage bakanae disease in an economic and eco-friendly way, the goal of this study was to use GWAS to mine rice accessions and genes/loci for resistance to *F. fujikuroi*. Rice Diversity Panel 1 (RDP1), which is estimated to have an average gene diversity of 0.68 (Ali et al. 2011), is a well-known open access collection of 421 diverse accessions from 79 countries (Ali et al. 2011; Eizenga et al. 2014). Several studies have used RDP1 to successfully identify QTLs controlling resistance to major rice diseases such as rice blast (Kang et al. 2016; Mgonja et al. 2016; Zhu et al. 2016; Lin et al. 2018), sheath blight (Chen et al. 2019), and bacterial blight (Li et al. 2018). However, bakanae resistance in RDP1 remains to be explored. In this study, RDP1 was inoculated with a highly virulent *F. fujikuroi* isolate and assessed for resistance by performing visual rating and reisolation of *F. fujikuroi* from the basal stems of infected rice seedlings. Novel QTLs for disease severity and pathogen colonization were identified. Furthermore, a candidate QTL co-localizing with *qBK1* and *qFfR1* was validated using a bi-parental population and narrowed down by sequence analysis. The genetic information from RDP1 can provide a useful basis for resistance breeding and uncovering the resistance mechanisms for bakanae disease.

## Materials and methods

### Plant and fungal materials

GWAS and linkage mapping were performed using 231 RDP1 accessions and 132 F_10_ recombinant inbred lines (RILs) derived from an IR64 x Nipponbare cross (Yan et al. 2015), respectively. RDP1 was provided by the Genetics Stocks Oryza (GSOR) germplasm collection (Agricultural Research Service, US Department of Agriculture) and the RILs were provided by Dr. Susan McCouch from Cornell University. Because some rice varieties did not grow or reproduce well in the greenhouse at Kaohsiung District Agriculture Research and Extension Station, Taiwan Agricultural Research Institute, or in the Phytotron at National Taiwan University, a sufficient number of seeds were only available for some of the RDP1 accessions and RIL population lines. RDP1 was classified into *indica*, *aus*, *temperate japonica*, *tropical japonica* and *aromatic* subpopulations, and the accessions that did not fit into any subpopulation were defined as admixed accessions (Zhao et al. 2011). The 231 accessions tested in this study were 43 *indica*, 33 *aus*, 57 *temperate japonica*, 63 *tropical japonica*, 3 *aromatic*, and 32 admixed varieties (Additional file 1: Table S1). According to genetic relatedness (Kovach et al. 2007), the all population was further divided into *indica* (*indica* and *aus*) and *japonica* (*temperate japonica*, *tropical japonica*, and *aromatic*) varietal subgroups in this study.

A highly virulent *F. fujikuroi* isolate, Ff266, isolated from a diseased adult rice plant collected from Ilan in 2012 (Chen et al. 2016), was used for evaluation of bakanae resistance. Ff266 was one of 24 representative isolates selected based on the genetic analysis of 637 *F. fujikuroi* isolates collected from 14 counties/cities around Taiwan from 1996 to 2013. Evaluation of the 24 representative isolates on 8 rice varieties suggested no clear pattern of specific variety x isolate interaction (Chen et al. 2016); therefore, we only used Ff266, which grows well on artificial media, as the inoculum.

### Evaluation of bakanae disease resistance

Inoculation was conducted following methods modified from Chen et al. (2016) and Kim et al. (2014). *F. fujikuroi* Ff266 was cultured on 1/2 potato dextrose agar for 4 days at 25 °C under a 12/12-h light/dark photoperiod. The spores were collected in sterile dH2O, filtered through Kimwipes, and adjusted to 10^5^ spores/mL. Rice seeds were put in a cassette and disinfected in sterile water at 60 °C for 10 min, then immersed in water at room temperature for 4 days. The pre-germinated seeds were then soaked in the spore suspension or sterile water (as a control) and shaken for 1 h. The seeds were sown in Akadama soil and cultivated in a walk-in incubator (32/28 °C day/night temperature, 12/12-h light/dark photoperiod, luminous intensity 7000–8000 lx). Twenty-one days after inoculation, two methods (visual disease assessment and quantification of *F. fujikuroi* colonization, as described below) were used to evaluate resistance to bakanae disease. Based on our preliminary test, 21 days post inoculation was the time point that most susceptible rice accessions showed severe bakanae symptoms, and different levels of resistance in RDP1 were easily distinguished.

For GWAS, inoculation of the 331 diverse accessions was conducted in two independent trials, each containing 10 seedlings per rice accession per treatment grown in a single pot (L x W x H = 3.5 × 4.5 × 5.5 cm). The experiment was performed following a randomized complete block design, with 48 accessions, 1 resistant control and 1 susceptible control in each block. A total of 231 accessions with at least 4 seedlings per treatment in each trial were used in further analysis. For linkage analysis using the 132 RILs, there were 16–30 seedlings for each treatment (3 pots per RIL per treatment, 4–10 seedlings per pot).

Visual disease assessment was conducted by naked-eye examination. Each infected seedling was compared to healthy ones (from the sterile water treatment) and rated based on a 0–3 scale (Chen et al. 2016). The overall disease severity index for each accession was calculated as: $\frac{\sum \mathrm{scale}\ \mathrm{x}\ \mathrm{No}.\mathrm{of}\ \mathrm{seedlings}\ \mathrm{with}\ \mathrm{the}\ \mathrm{scale}\ }{\operatorname{Max}.\mathrm{scale}\ \mathrm{x}\ \mathrm{Total}\ \mathrm{no}.\mathrm{of}\ \mathrm{seedlings}}\ \mathrm{X}\ 100\%$.

To examine the colonization rate of *F. fujikuroi* in a rice seedling, whole inoculated seedling was surface-sterilized by spraying with 75% EtOH and a 2-cm segment (1–3 cm from the stem base) was excised and placed on FFC selective medium (Hsu 2013). After 7 days of cultivation at 25 °C under 12/12-h light/dark photoperiod, stem segments from which *F. fujikuroi* could be re-isolated were counted (the distinct orange colonies of *F. fujikuroi* could grow out from two ends of the colonized segment). The colonization rate for each accession was calculated as: $\frac{\mathrm{No}.\mathrm{of}\ \mathrm{segments}\ \mathrm{showing}\ F. fujikuroi\ \mathrm{colonies}}{\mathrm{Total}\ \mathrm{no}.\mathrm{of}\ \mathrm{segments}}\mathrm{X}\ 100\%$. This method was modified from that used in our previous evaluation of *F. fujikuroi* Ff266 colonization on eight rice varieties (Chen et al. 2015). Among five consecutive 1-cm-segments of the basal stem, significant differences between resistant and susceptible varieties were consistently observed from the segments 1–2 cm and 2–3 cm from the base of the infected seedlings. Both resistant and susceptible varieties showed re-isolation frequencies > 90.5% from the 0–1 cm segment and < 40.6% from the 4–5 cm segment, so these segments were excluded from the colonization test in this study.

### Genome-wide association mapping

The 44 K single nucleotide polymorphism (SNP) data for RDP1 were downloaded from http://www.ricediversity.org/index.cfm (Zhao et al. 2011). The SNPs with a minor allele frequency (MAF) < 0.05 were excluded. To control for variation among blocks in different inoculation trials, best linear unbiased estimates (BLUEs) for phenotypic data were generated using TASSEL 5.2.24 (Bradbury et al. 2007). Association analyses using three datasets (all population, *indica*, and *japonica*) and two traits (disease severity index and colonization rate) were conducted in TASSEL 5.2.24.

A generalized linear model (GLM) and a mixed linear model (MLM) were used for association mapping. The formula for the GLM was *y = Xβ + e*, which includes the vector of phenotypic data (*y*), the matrix of genotype and population structure (*X*), the vector of genotype and population structure (*β*), and the vector of residuals (*e*). The formula for the MLM was *y = Xβ + Zu + e*, which additionally includes the vector estimated from the kinship matrix (*u*) and the known design matrices (*Z*) (Bradbury et al. 2007). In both models, the population structure (Q) and kinship (K) were obtained by performing principal component analysis (PCA) in TASSEL 5.2.24. For the all population, we tested GLM and MLM with/without the population structure as covariates (GLM, GLM-Q, MLM-K, MLM-K + Q); for the *indica* and *japonica* sub-populations, GLM and MLM-K were tested. Quantile-Quantile (Q-Q) plots and Manhattan plots were generated using the qqman package in R (R Development Core Team). The fitness of different models for each phenotype dataset was determined based on the Q-Q plots.

The genomic regions containing more than five significant SNPs with *P* < 10^− 3^ [−Log_10_(*P*) > 3] within 200 kb were considered candidate QTLs. The QTL intervals were defined by linkage disequilibrium (LD) blocks calculated using Haploview 4.2 (Barrett et al. 2005). Putative resistance and susceptibility haplotypes at each candidate QTL (significant LD block) were defined by Chi-square analysis using Haploview 4.2. Because binary data were used for the haplotype analysis in Haploview, accessions with disease severity indexes > 0.3 and ≤ 0.3 were arbitrarily assigned as resistant (R) and susceptible (S), respectively; accessions with colonization rates > 0.3 and ≤ 0.3 were arbitrarily assigned as R and S, respectively. Genes located in candidate QTLs were annotated according to the Nipponbare reference genome (MSU v7.0). Gene descriptions were acquired from The Rice Annotation Project website (http://rapdb.dna.affrc.go.jp/index.html) (Sakai et al. 2013; Kawahara et al. 2013).

### Linkage mapping

Genotypic data of the IR64 x Nipponbare RILs (total 35,460 SNPs) were derived from genotyping by sequencing (Yan et al. 2015). Recombination breakpoints were inferred from the 35,460 SNPs, and the first SNP of each recombination bin was assigned as a bin marker. Linkage mapping was conducted with 7466 bin markers by R/qtl (Broman et al. 2003). All heterozygous genotypes were defined as missing values. Genetic map was constructed based on Kosambi function. Composite interval mapping (CIM) was performed to detect the QTLs controlling disease severity index. The logarithm of odds (LOD) thresholds were determined based on 1000 permutations.

### Sequencing and candidate gene analysis for *qBK1.7*

Full-length sequences of *qBK1.7* (~ 8.3 kb) in 20 accessions were obtained using Sanger sequencing. The 20 accessions included 11 accessions carrying the resistance haplotype [IR64 (NSFTV_644), NSFTV_18, NSFTV_19, NSFTV_74, NSFTV_85, NSFTV_137, NSFTV_161, NSFTV_171, NSFTV_209, NSFTV_313 and NSFTV_337] and 9 accessions carrying the susceptibility haplotype [Nipponbare (NSFTV_173), NSFTV_17, NSFTV_66, NSFTV_110, NSFTV_138, NSFTV_145, NSFTV_252, NSFTV_255 and NSFTV_304] at *qBK1.7*. Genomic DNA was extracted from rice leaves following a standard cetyltrimethylammonium bromide (CTAB) extraction protocol (Doyle 1987). Primers used for the sequencing of *qBK1.7* are listed in Additional file 2: Table S2. PCR was run using Taq DNA Polymerase 2x Master Mix RED (Ampliqon, Denmark) following the manufacturer’s protocol. Sequence alignment and amino acid translation were conducted using Vector NTI 11 (Invitrogen, USA). The association between the sequences and disease severity index was assessed using the GLM (as mentioned above) in TASSEL 5.2.24.

### Statistical analysis

All statistical analyses were conducted using SAS Enterprise Guide 6.1 (SAS Institute, Cary, NC). Analysis of variance (ANOVA) and Student’s *t*-test were performed to analyze the phenotypic differences between subpopulations (*indica* and *japonica*). Pearson correlation analysis was used to examine the correlation between disease severity index and colonization rate.

## Results

### Bakanae disease resistance in RDP1

**Fig. 1:**
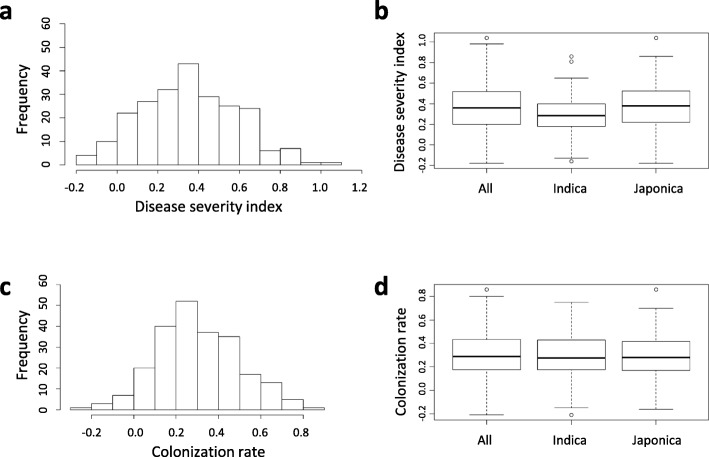
Distribution of bakanae disease resistance scores. The histograms show the distribution of resistance scores in the all population, and boxplots show the phenotypic distributions of the all population and the two subgroups. (**a** and **b**) Disease severity index; (**c** and **d**) Colonization rate

**Fig. 2:**
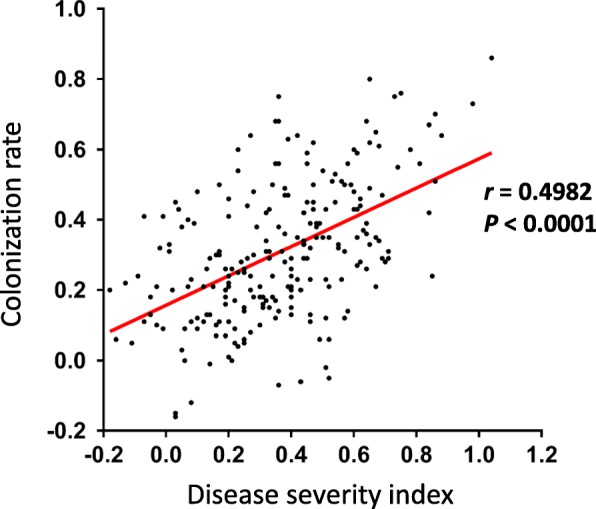
Correlation between disease severity index and colonization rate. *r*: Pearson correlation coefficient

The *F. fujikuroi*-inoculated seedlings showed multiple morphological symptoms. In general, the infected plants were elongated and slender and had a wide leaf angle. Elongation of the second internode, the second leaf, and the third leaf could also be observed. For the 231 accessions tested, phenotypic values for disease severity index and colonization rate were consistent between the two trials (Additional file 1: Table S1). The distributions of disease severity index and colonization rate are shown for the all population and the *indica* and *japonica* subgroups (Fig. 1). In the all population, disease severity index ranged from − 0.18 to 1.04 and colonization rate ranged from − 0.21 to 0.86. Ninety-two accessions showed good resistance based on disease severity (disease severity index ≤0.3), and 120 accessions had good resistance based on colonization rate (colonization rate ≤ 0.3). Significantly lower disease severity indices were observed for the *indica* than *japonica* subgroup (*p* = 0.0071; average disease severity indices of the *indica* and *japonica* subgroups were 0.29 and 0.37, respectively) (Fig. 1b). No significant difference in colonization rate was found between the two subgroups (Fig. 1d). Disease severity index and colonization rate were moderately correlated (Fig. 2; Pearson correlation coefficient *r* = 0.498, *p* < 0.0001).

### Genome-wide association mapping in RDP1

**Fig. 3:**
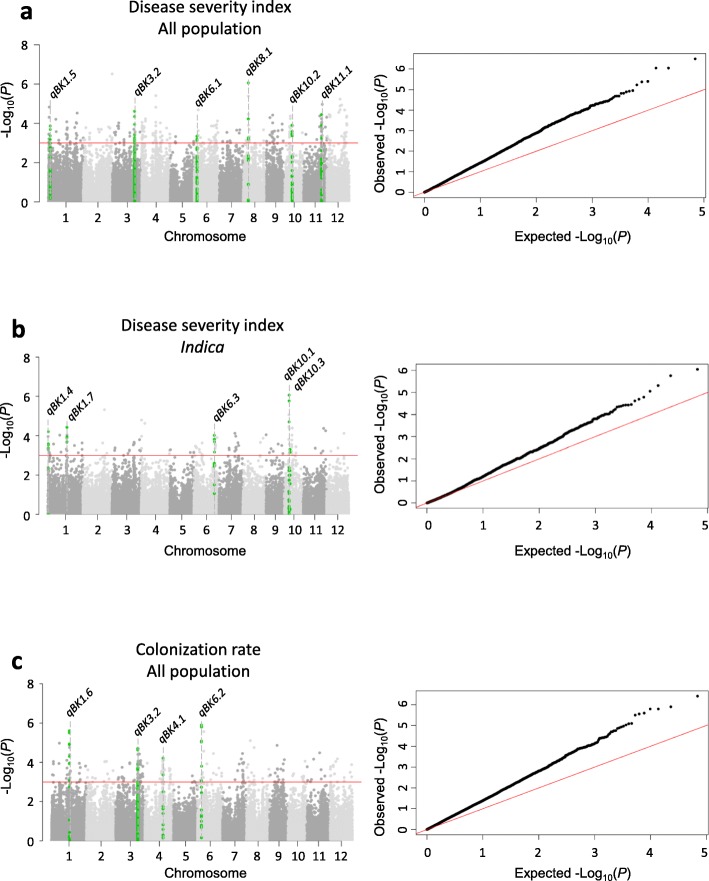
Genome-wide association mapping in the Rice Diversity Panel 1. Loci significantly associated with bakanae resistance were identified for (**a**) disease severity index in the all population, (**b**) disease severity index in the *indica* subgroup, and (**c**) colonization rate in the all population. Manhattan plots on the left show significant genomic regions identified for disease severity index (**a** and **b**) and colonization rate (**c**). X axis: rice chromosomes; Y axis: -Log_10_(*P*). Q-Q plots on the right show the fitness of the selected models used for different traits in the all population or subgroups. X axis: expected –Log_10_(*P*); Y axis: observed –Log_10_(*P*)

The results of GWAS and model fitness are shown in Manhattan plots and Q-Q plots (Fig. 3). The models GLM-Q and GLM were optimal for GWAS at the all population and subgroup levels, respectively. A total of 14 candidate QTLs were located on chromosomes 1, 3, 4, 6, 8, 10, and 11: 6 QTLs for severity in the all population, 5 QTLs for severity in the *indica* subgroup, and 4 QTLs for colonization rate in the all population (Table 2 and Fig. 3). Only *qBK3.2* was identified for both severity and colonization rate. No candidate QTL was mapped for the *japonica* subgroup.

**Table 2:** Candidate QTLs associated with resistance to bakanae disease

QTL	Population	Trait	Chr	QTL region (bp)^a^	Genes within region	*R*^2 b^
*qBK1.4*	*Indica*	Severity index	1	401,383-426,527	5	0.27
*qBK1.5*	All	Severity index	1	2,254,725-2,329,243	10	0.18
*qBK1.6*	All	Colonization rate	1	22,088,636-22,248,843	12	0.28
*qBK1.7*	*Indica*	Severity index	1	23,631,992-23,640,231	3	0.25
*qBK3.2*	All	Colonization rate	3	27,480,288-27,635,650	12	0.19
	All	Severity index	3	27,480,288-27,635,650	12	0.22
*qBK4.1*	All	Colonization rate	4	22,371,038-22,428,157	14	0.33
*qBK6.1*	All	Severity index	6	3,276,254-3,639,339	49	0.22
*qBK6.2*	All	Colonization rate	6	4,866,345-5,059,806	29	0.28
*qBK6.3*	*Indica*	Severity index	6	25,298,288-25,638,876	19	0.20
*qBK8.1*	All	Severity index	8	6,142,736-6,239,144	15	0.28
*qBK10.1*	*Indica*	Severity index	10	5,678,051-6,024,705	20	0.25
*qBK10.2*	All	Severity index	10	6,849,663-6,864,693	1	0.21
*qBK10.3*	*Indica*	Severity index	10	9,090,969-9,337,961	16	0.26
*qBK11.1*	All	Severity index	11	22,576,995-22,582,906	1	0.13

^a^The genomic position is based on the MSU7 Nipponbare reference genome

^b^*R*^2^ represents the phenotypic variation explained by the most significant SNP in the QTL

A total of 206 genes were identified within the regions of candidate QTLs (Additional file 3: Table S3). These included five genes containing a leucine-rich repeat (LRR) domain (*Os01g0601625*, *Os01g0601675*, *Os06g0167500*, *Os06g0627500*, and *Os10g0183000*; *Os10g0183000* is an NB-LRR gene), two genes containing an NB-ARC (nucleotide-binding adaptor shared by APAF-1, R proteins, and CED-4) domain (*Os03g0689400* and *Os03g0689833*), and an OsWAK receptor-like protein kinase (RLK) gene (*Os06g0170100*). Three hormone-related genes were found: *Os04g0448900* is involved in abscisic acid (ABA) biosynthetic process, and *Os06g0166500* and *Os06g0196700* are related to the auxin signaling pathway. Among the other annotated candidate genes, six genes encode oxidases/oxidoreductases (*Os01g0574600*, *Os03g0690500*, *Os04g0448900*, *Os06g0168600*, *Os06g0196300*, and *Os06g0632300*), nine genes encode transcription factors (*Os01g0575200*, *Os03g0690600*, *Os06g0164400*, *Os06g0165600*, *Os06g0166100*, *Os06g0166400*, *Os06g0166500*, *Os06g0171700*, and *Os08g0206500*), and three genes are related to ubiquitination (*Os01g0141700*, *Os06g0167200*, and *Os06g0167600*). In addition, a gene associated with growth regulation (*Os06g0199500*) was identified.

### Resistance and susceptibility haplotypes in RDP1

Putative resistance and susceptibility haplotypes of the 14 candidate QTLs in 231 accessions are shown in Additional file 4: Table S4. The numbers of resistance haplotypes and susceptibility haplotypes in an accession ranged from 0 to 6 and 0–4, respectively. The total numbers of R and S haplotypes were negatively (*r* = − 0.3, *P* < 0.001) and positively (*r* = 0.29, *P* < 0.001) correlated with disease severity index, respectively (Additional file 5: Table S5). The total number of S haplotypes was also negatively correlated with colonization rate (*r* = 0.22, *P* < 0.001).

### Validation of *qBK1.7* in the IR64 x Nipponbare population

**Fig. 4:**
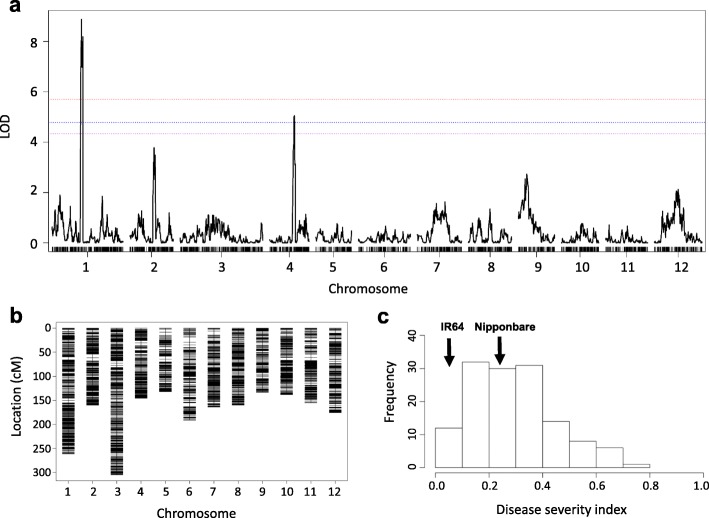
Linkage mapping in the IR64 x Nipponbare population. (**a**) QTLs detected by composite interval mapping. The horizontal lines represent the logarithm of odds (LOD) thresholds at 90% (purple), 95% (blue), and 99% (red) confidence levels based on 1000 permutations; (**b**) Genetic map of 7466 bin markers; (**c**) Frequency distribution of the disease severity index in the 132 recombinant inbred lines

A set of 132 F_10_ RILs derived from IR64 x Nipponbare (both parents belong to RDP1) was available to us. Haplotype analysis suggested that IR64 (NSFTV_644) may contain resistance haplotypes at *qBK1.5*, *qBK1.7*, and *qBK6.3*, whereas Nipponbare (NSFTV_173) may contain a resistance haplotype at *qBK4.1* (Additional file 4: Table S4). To confirm the effects of the QTLs associated with disease severity index, linkage mapping was conducted using the IR64 x Nipponbare population. The LOD thresholds at 90%, 95%, and 99% confidence levels were 4.33, 4.78 and 5.70, respectively. Two QTL peaks (LOD > 4.33) were observed on chromosomes 1 and 4 (Fig. 4). The most significant QTL (LOD = 8.88) was identified between S1_22464052 (22.46 Mb) and S1_23641393 (23.64 Mb). This region was colocalized with *qBK1.7* (23,63–23,64 Mb). The other QTL (LOD = 5.05) was identified between S4_22371369 (22.55 Mb) and S4_23129489 (23.31 Mb), which was close to *qBK4.1* (22,37–22,42 Mb). *qBK1.5* and *qBK6.3* were not detected in the IR64 x Nipponbare population, perhaps due to their minor effects or epistatic interactions with the genetic background.

### Sequence analysis of *qBK1.7*

To identify the causal element(s) of *qBK1.7* (~ 8.3-kb; 23,631,992-23,640,231 bp), we sequenced a ~ 12-kb region (23,630,923-23,642,918 bp) covering *qBK1.7* in 20 rice accessions carrying the resistance or susceptibility haplotype (Additional file 6: Fig. S1). Among 743 SNPs across the *qBK1.7* region, 310 SNPs had values of -Log_10_(*P*) > 2. 230, and 70 SNPs with -Log_10_(*P*) > 2 were located in the coding regions of *Os01g0601625* and *Os01g0601675*. For *Os01g0601625*, in addition to 16 nonsynonymous SNPs with -Log_10_(*P*) > 2, a 210-bp deletion (23,633,925-23,634,134 bp) causing the deletion of 70 amino acids was identified, which is a notable difference between the accessions carrying resistance and susceptibility haplotypes (Additional file 7: Fig. S2). Twenty-four nonsynonymous SNPs with -Log_10_(*P*) > 2 were located within *Os01g0601675*. In addition, eight accessions carrying the susceptibility haplotype (not including Nipponbare) contain a premature termination codon in *Os01g0601675* (Additional file 8: Fig. S3), which might cause a C-terminally truncated translation product. The nonsynonymous substitutions, deletion, and early termination would affect the structures and functions of Os01g0601625 and Os01g0601675.

## Discussion

Compared with the abundant knowledge of the genetics underlying resistance to other rice diseases (e.g., rice blast and bacterial blight), knowledge of the genetic basis for rice resistance to bakanae disease is limited. Based on the literature, large-scale screening of bakanae resistance has only been conducted on 72 Korean varieties (Lee et al. 2011), 92 Indian varieties and landraces (Fiyaz et al. 2014), and a collection of 138 (67 domestic and 71 foreign) *japonica* rice varieties from the rice germplasm in Italy (Volante et al. 2017). To better represent worldwide rice diversity in bakanae disease resistance, we used a high-throughput inoculation system to evaluate the resistance performance of 231 diverse accessions from the open-access RDP1. A total of 14 QTLs were identified: 13 novel QTLs and a QTL co-localizing with the known QTLs *qBK1* and *qFfR1* (Hur et al. 2015; Ji et al. 2017; Lee et al. 2019). Resistance and susceptibility haplotypes of the 14 QTLs were also determined. This information should help selection of rice accessions with favorable alleles at the QTLs of interest. Eleven accessions (NSFTV_18, NSFTV_74, NSFTV_85, NSFTV_220, NSFTV_243, NSFTV_250, NSFTV_251, NSFTV_257, NSFTV_325, NSFTV_327, NSFTV_337) that showed both low disease severity and low colonization rate can be used as donors in resistance breeding programs.

Colonization rate is a new parameter used to map QTLs associated with bakanae resistance. In six previous QTL studies, bakanae resistance was assessed by determining mortality rate, incidence, disease index, and seedling height, which were all based on direct observation or quantification of visual symptoms (Table 1) (Yang et al. 2006; Hur et al. 2015; Fiyaz et al. 2016; Volante et al. 2017; Ji et al. 2017; Lee et al. 2018). In this study, distinct sets of QTLs were identified from two different traits: disease severity index and colonization rate. By examining the frequency of isolation of *F. fujikuroi* from the basal stem, three additional novel QTLs were mapped. A weak positive correlation (*r* = 0.498) was observed between disease severity index and colonization rate. Some rice accessions showed different levels of resistance/susceptibility based on the two different traits. Rice accessions NSFTV_99, NSFTV_120, NSFTV_131, NSFTV_284, and NSFTV_643 had a low level of *F. fujikuroi* colonization (colonization rate < 0.06) but susceptible symptoms (disease severity index = 0.43–0.52); NSFTV_246, NSFTV_252, and NSFTV_395 had a high level of *F. fujikuroi* colonization (colonization rate = 0.68–0.75) but moderate symptoms (disease severity index = 0.35–0.36). These results suggest different mechanisms control the development of bakanae symptoms and the spread/colonization of *F. fujikuroi* in rice seedlings.

A plant can utilize different strategies to protect itself against invading pathogens. Wheat resistance to *Fusarium* head blight caused by *F. graminearum* has been classified into two types: type I is the resistance to initial infection and type II is the resistance to fungal spread within the head (Schroeder and Christensen 1963). In the rice - *F. fujikuroi* pathosystem, disease symptoms such as abnormal elongation or stunting have been associated with gibberellin (GA), fusaric acid, fumonisins and/or novel secondary metabolites produced by *F. fujikuroi* (Nyvall 1989; Niehaus et al. 2017). The morphological changes of infected seedlings (i.e., the observed disease severity) may be affected by not only the quantities/types of fungal secondary metabolites (which are related to the level of *F. fujikuroi* colonization) but also the sensitivity of the host in responding to them. It would be interesting to further investigate how the secondary metabolites and effectors of *F. fujikuroi* interfere with the regulation of growth, development, and defenses in rice. Because the appearance of bakanae symptoms may not fully reflect the level of resistance in rice, quantification of the level of *F. fujikuroi* colonization (by isolation using a selective medium or by qPCR) can be a good complement to the conventional disease severity rating. For resistance breeding, incorporation of QTL(s) for colonization resistance will help lower the population of *F. fujikuroi* in the field.

**Fig. 5:**
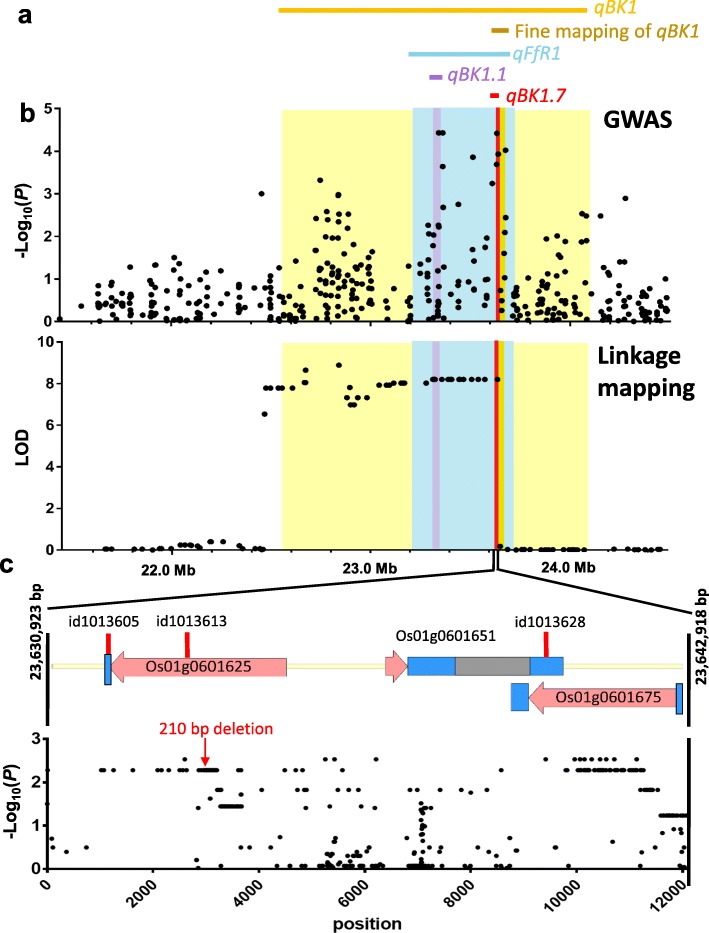
The location and sequence analysis of *qBK1.7*. (**a**) The positions of *qBK1.7* and previously identified QTLs. (**b**) Manhattan plots showing the results from genome-wide association study (GWAS) and linkage mapping in this study. (**c**) Analysis of the association between bakanae resistance and *qBK1.7* sequences across 11 accessions carrying the resistance haplotype and 9 accessions carrying the susceptibility haplotype. The pink, gray, and blue bars represent the predicted exons, introns, and untranslated regions, respectively, obtained from The Rice Annotation Project website

Among the 14 QTLs identified in RDP1 by GWAS, *qBK1.7* was further validated in an F_10_ RIL population of 132 individuals derived from a cross between IR64 and Nipponbare. Three co-localized QTLs (*qFfR1*, *qBK1*, and *qBK1.1*) were mapped to the same genomic region on the long arm of rice chromosome 1 in different bi-parental populations using different *F. fujikuroi* isolates, suggesting that this locus accounts for a significant proportion of bakanae resistance in rice germplasm. The major QTL was first designated *qBK1* and located to a 520-kb region (23.21–23.72 Mb) using 168 near-isogenic lines (NILs, BC_6_F_4_) (Hur et al. 2015). *qBK1.1* (23.32–23.34 Mb) (Fiyaz et al. 2016) and *qFfR1* (22.56–24.10 Mb) (Ji et al. 2017) were then mapped by linkage analysis using 180 F_3_ families and 168 F_14_ RILs, respectively (Fig. 5). Using seven homozygous RILs derived from 1485 BC_7_F_4_ plants segregating for the target region, a recent study fine mapped *qBK1* to a region (23.637–23.672 Mb) containing 4 candidate genes (*Os01g0601675*, *Os01g0601700*, *Os01g0601950*, and *Os01g0602200*) (Lee et al. 2019). In this study, GWAS identified the QTL at high resolution: *qBK1.7* was mapped to an 8239-bp region (23.632–23.640 Mb) containing *Os01g0601625*, *Os01g0601651*, and *Os01g0601675* (Fig. 5). *qBK1.7* and the fine-mapped *qBK1* (Lee et al. 2019) overlap at 23.637–23.640 Mb, with a common candidate gene *Os01g0601675*. Analysis of the sequences across the *qBK1.7* region in 20 rice accessions revealed that the coding regions of *Os01g0601625* and *Os01g0601675* may be associated with bakanae resistance. Of note, a 210-bp deletion was found in *Os01g0601625* in all 11 accessions carrying the resistance haplotype and a premature termination codon was detected in *Os01g0601675* in 8 of 9 accessions carrying the susceptibility haplotype. The intergenic regions and *Os01g0601651* are unlikely the causal element because of the generally low -Log_10_(*P*) values and the lack of a start codon in *Os01g0601651*. Both *Os01g0601625* and *Os01g0601675* are annotated as genes encoding LRR proteins, which may play a role in recognition and signaling in pathogen-associated molecular pattern-triggered immunity or effector-triggered immunity (Jones and Jones 1997; Padmanabhan et al. 2009).

A total of 206 candidate genes are located within the 14 QTLs for bakanae resistance. Among the six LRR- and NB-ARC-containing genes identified in *qBK1.7*, *qBK3.2*, *qBK6.1*, and *qBK6.3*, the NB-LRR gene *Os10g0183000* in *qBK10.1* has been shown to play a role in disease resistance. Changes in *Os10g0183000* expression caused accumulation of thiamine and activation of immune response in rice (Wang et al. 2016). An *OsWAK RLK* gene *Os06g0170100* was identified in *qBK6.1*. Some wall-associated kinases (WAKs) in rice are known to positively or negatively regulate basal defense and quantitative resistance to *Magnaporthe oryzae* (Delteil et al. 2016). Genes related to ABA biosynthesis (*Os04g0448900* in *qBK4.1*), auxin signaling (*Os06g0166500* in *qBK6.1* and *Os06g0196700* in *qBK6.2*), and GA signaling (a *scarecrow-like 3* gene *Os03g0690600* in *qBK3.2*) were also identified. Phytohormones ABA and auxin are important in regulating plant growth and development (Vishwakarma et al. 2017; Wang et al. 2018), as well as defense responses to various biotic/abiotic stresses (Cohen and Leach 2019). In Arabidopsis, SCARECROW-LIKE 3 antagonizes DELLA (a master repressor of GA responses) and controls GA-biosynthetic and -responsive genes (Zhang et al. 2011). The roles of plant hormones and their interplay in rice immunity and the development of bakanae symptoms warrant further exploration.

## Conclusions

Complex morphological symptoms are characteristics of rice bakanae disease. In this study, levels of bakanae resistance in 231 diverse rice accessions were evaluated based on visual symptoms as well as the colonization rate of *F. fujikuroi*. The results suggest that different mechanisms underlie these two traits. While 11 QTLs associated with bakanae resistance were previously mapped (Table 1), this study identified 14 QTLs (including 13 novel QTLs) (Table 2) and 206 candidate genes (Additional file 2: Table S2), and provided information on putative resistance and susceptibility haplotypes of the 14 QTLs for each tested rice accession (Additional file 4: Table S4). The new QTLs, particularly *qBK3.2*, which was associated with both traits, can be useful in resistance breeding. Moreover, a QTL (*qBK1.7*) co-localizing with *qBK1* and *qFfR1* (Hur et al. 2015; Ji et al. 2017; Lee et al. 2019) was delineated to a region of 8239 bp, which overlaps with the *qBK1* region (35 kb) fine-mapped using homozygous recombinant lines (Lee et al. 2019). Significant differences in the sequences of two LRR-containing genes (*Os01g0601625* and *Os01g0601675*) located within *qBK1.7* were found between 11 accessions carrying the resistance haplotype and 9 accessions carrying the susceptibility haplotype. Cloning and functional characterization of these two genes will reveal their potential roles in bakanae resistance.

## Supplementary information

**Additional file 1: Table S1.** Rice accessions and their resistance performance based on disease severity index and colonization rate.

**Additional file 2: Table S2.** Primers used for sequencing analysis of *qBK1.7.*

**Additional file 3: Table S3.** Candidate genes within the regions associated with resistance to bakanae disease.

**Additional file 4: Table S4.** Resistance and susceptibility haplotypes in each rice accession.

**Additional file 5: Table S5.** Pearson correlation coefficient (*r*) values for the association between resistance and the total number of non-overlapping resistance (R) or susceptible (S) haplotypes’.

**Additional file 6: Fig. S1.** Alignment of the nucleotide sequences from 1069 bp upstream to 2687 bp downstream of *qBK1.7*.

**Additional file 7: Fig. S2.** Alignment of the amino acid sequences of *Os01g0601625.*

**Additional file 8: Fig. S3.** Alignment of the amino acid sequences of *Os01g0601675.*

## Data Availability

The datasets used and analyzed during the current study are available from the corresponding author on reasonable request.

## Abbreviations

ABA: Abscisic acid
ANOVA: Analysis of variance
BLUEs: Best linear unbiased estimates
CIM: Composite interval mapping
CTAB: Cetyltrimethylammonium bromide
GA: Gibberellin
GLM: Generalized linear model
GSOR: Genetics Stocks Oryza
GWAS: Genome-wide association study
LD: Linkage disequilibrium
LRR: Leucine-rich repeat
MLM: Mixed linear model
NB-ARC: Nucleotide-binding adaptor shared by APAF-1, R proteins, and CED-4
PAMP: Pathogen-associated molecular pattern
PCA: Principal component analysis
Q-Q: Quantile-Quantile
QTLs: Quantitative trait loci
R: Resistant
RDP1: Rice Diversity Panel 1
RILs: Recombinant inbred lines
RLK: Receptor-like protein kinase
S: Susceptible
SCL3: Scarecrow-LIKE 3
SNPs: Single nucleotide polymorphisms
WAKs: Wall-associated kinases
