# Supplementary material for: Genome-wide association mapping of gene loci affecting disease resistance in the rice-Fusarium fujikuroi pathosystem
Source: Rice (N Y). 2019 Nov 21;12:85. doi: 10.1186/s12284-019-0337-3 (PMC6872702; doi:10.1186/s12284-019-0337-3)
Supplement: Supplementary file 6 — Additional file 6: Fig. S1. Alignment of the nucleotide sequences from 1069 bp upstream to 2687 bp downstream of qBK1.7. [file 12284_2019_337_MOESM6_ESM.pdf]

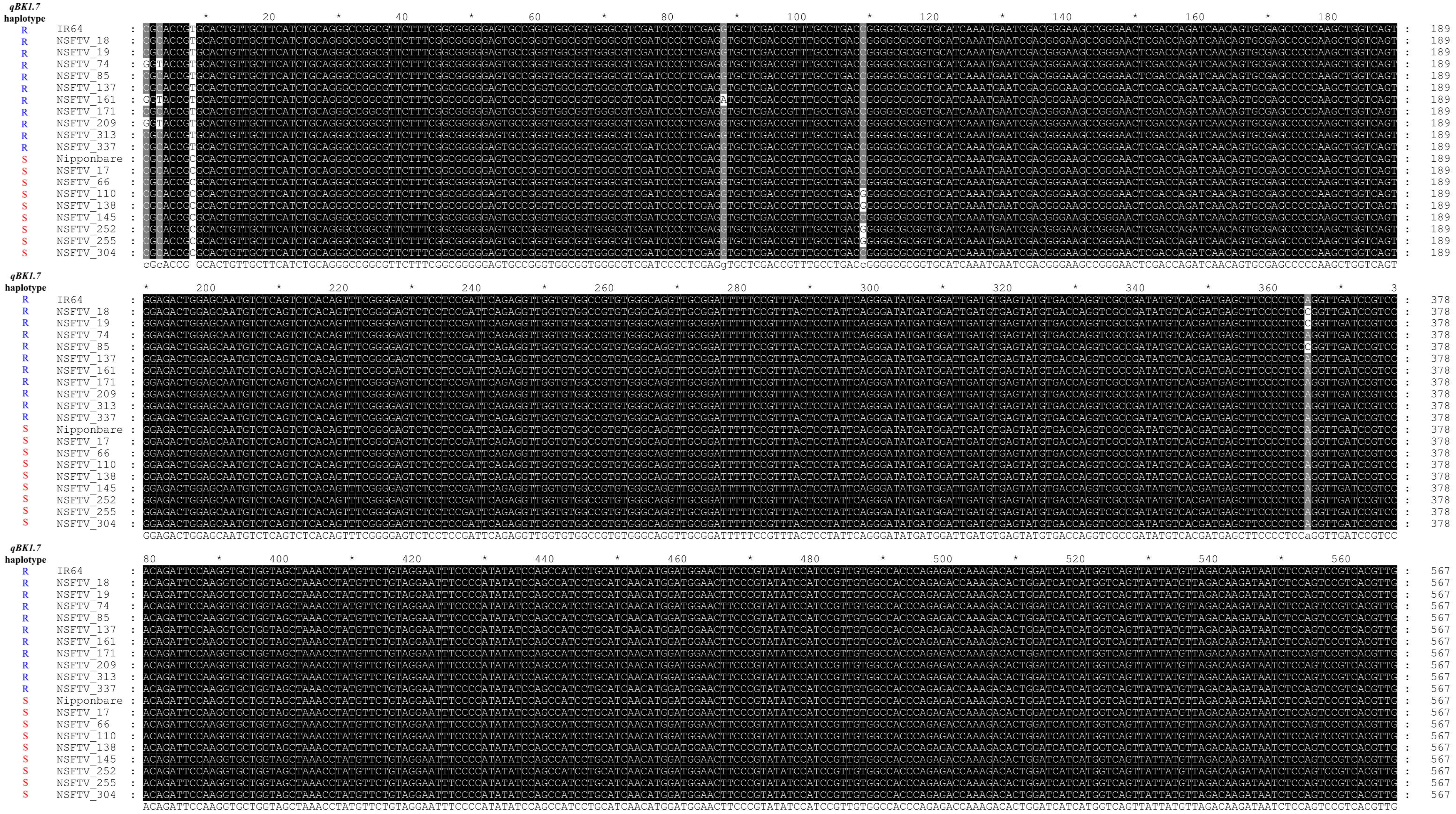
qBK1.7 haplotype
R IR64
R NSFTV_18
R NSFTV_19
R NSFTV_74
R NSFTV_85
R NSFTV_137
R NSFTV_161
R NSFTV_171
R NSFTV_209
R NSFTV_313
R NSFTV_337
S Nipponbare
S NSFTV_17
S NSFTV_66
S NSFTV_110
S NSFTV_138
S NSFTV_145
S NSFTV_252
S NSFTV_255
S NSFTV_304

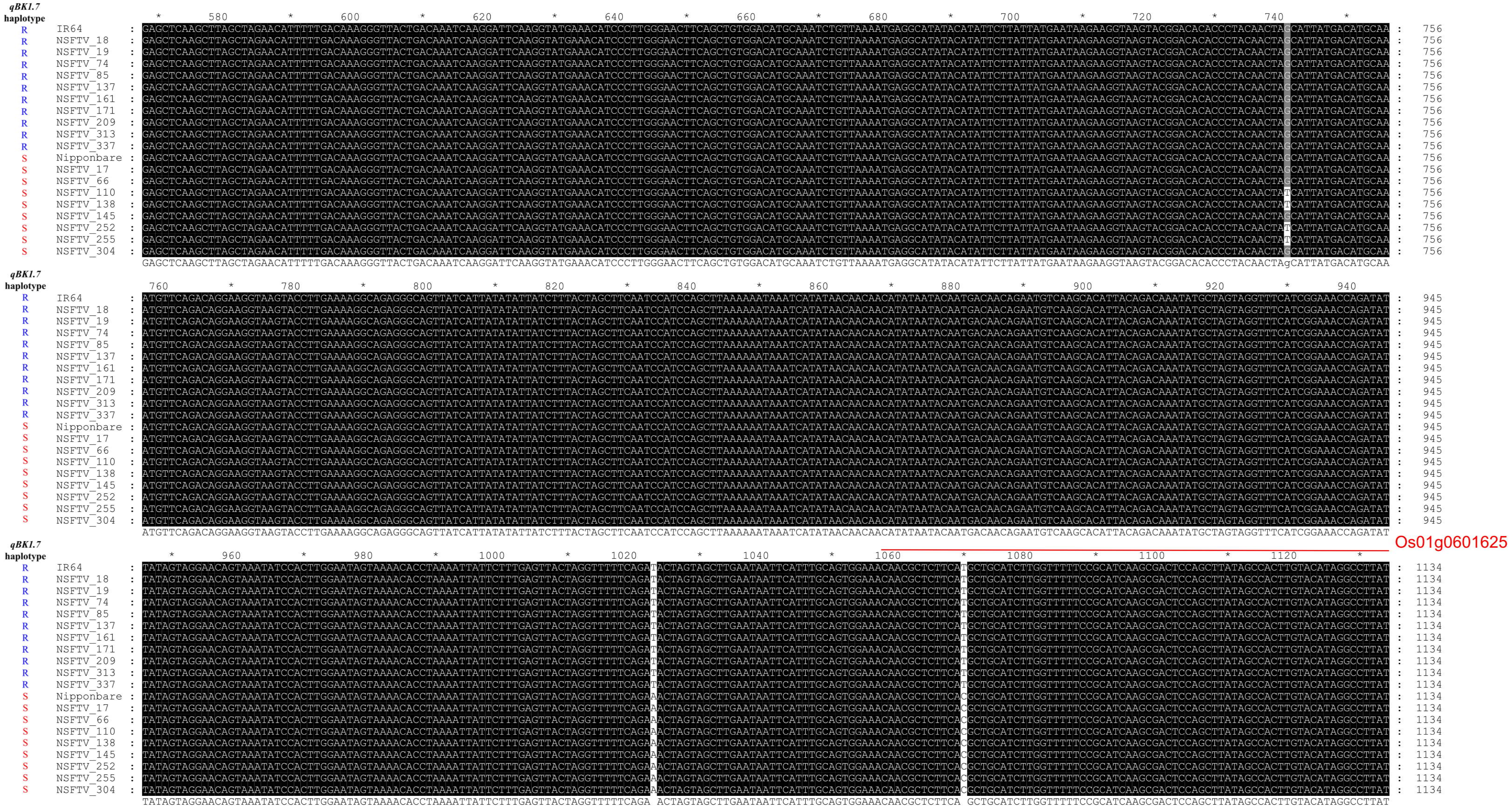
qBK1.7 haplotype
R IR64
R NSFTV_18
R NSFTV_19
R NSFTV_74
R NSFTV_85
R NSFTV_137
R NSFTV_161
R NSFTV_171
R NSFTV_209
R NSFTV_313
R NSFTV_337
S Nipponbare
S NSFTV_17
S NSFTV_66
S NSFTV_110
S NSFTV_138
S NSFTV_145
S NSFTV_252
S NSFTV_255
S NSFTV_304
GAGCTCAAGCTTAGCTAGAACATTTTTGACAAAGGGTTACTGACAAATCAAGGATTCAAGGTATGAAACATCCCTTGGGAACTTCAGCTGTGGACATGCAAATCTGTTAAAATGAGGCATATACATATTCTTATTATGAATAAGAAGGTAAGTACGGACACACCCTACAACTAgCATTATGACATGCAA
756
ATGTTCAGACAGGAAGGTAGTACCTTGAAAAGGCAGAGGGCAGTTATCATTATATATTATCTTTACTAGCTTCAATCCATCCAGCTTAAAAAATAAATCATATAACAACAACATATAATACAATGACAACAGAATGTCAAGCACATTACAGACAAATATGCTAGTAGGTTTCATCGGAAACCAGATAT
945
Os01g0601625
TATAGTAGGAACAGTAAATATCCACTTGGAATAGTAAAACACCTAAAATTATTCTTTGAGTTACTAGGTTTTTCAGA ACTAGTAGCTTGAATAATTCATTTGCAGTGGAAACAACGCTCTTCA GCTGCATCTTGGTTTTTCCGCATCAAGCGACTCCAGCTTATAGCCACTTGTACATAGGCCTTAT
1134

```
qBK1.7                                                                                                                                                        Os01g0601625
haplotype       1140         *        1160         *        1180         *        1200         *        1220         *        1240         *        1260         *        1280         *        1300         *        1320
R   IR64       : CATATAAATTGTCAATGATCCGAAAGTAAGCCATCATCCAGGTTCTCTTCATCATCATGGTGCAAAACACAGTCCATAGACCAATCACAAATCCTATGCTCATCCCGAGGTAGAGAGATCCCATATGGCTTCTATCTTCATAGAAACTTTGTTGTGTACCATTAGTTGAACAGTTCTTCAAGAGAGGAG : 1323
R   NSFTV_18   : CATATAAATTGTCAATGATCCGAAAGTAAGCCATCATCCAGGTTCTCTTCATCATCATGGTGCAAAACACAGTCCATAGACCAATCACAAATCCTATGCTCATCCCGAGGTAGAGAGATCCCATATGGCTTCTATCTTCATAGAAACTTTGTTGTGTACCATTAGTTGAACAGTTCTTCAAGAGAGGAG : 1323
R   NSFTV_19   : CATATAAATTGTCAATGATCCGAAAGTAAGCCATCATCCAGGTTCTCTTCATCATCATGGTGCAAAACACAGTCCATAGACCAATCACAAATCCTATGCTCATCCCGAGGTAGAGAGATCCCATATGGCTTCTATCTTCATAGAAACTTTGTTGTGTACCATTAGTTGAACAGTTCTTCAAGAGAGGAG : 1323
R   NSFTV_74   : CATATAAATTGTCAATGATCCGAAAGTAAGCCATCATCCAGGTTCTCTTCATCATCATGGTGCAAAACACAGTCCATAGACCAATCACAAATCCTATGCTCATCCCGAGGTAGAGAGATCCCATATGGCTTCTATCTTCATAGAAACTTTGTTGTGTACCATTAGTTGAACAGTTCTTCAAGAGAGGAG : 1323
R   NSFTV_85   : CATATAAATTGTCAATGATCCGAAAGTAAGCCATCATCCAGGTTCTCTTCATCATCATGGTGCAAAACACAGTCCATAGACCAATCACAAATCCTATGCTCATCCCGAGGTAGAGAGATCCCATATGGCTTCTATCTTCATAGAAACTTTGTTGTGTACCATTAGTTGAACAGTTCTTCAAGAGAGGAG : 1323
R   NSFTV_137  : CATATAAATTGTCAATGATCCGAAAGTAAGCCATCATCCAGGTTCTCTTCATCATCATGGTGCAAAACACAGTCCATAGACCAATCACAAATCCTATGCTCATCCCGAGGTAGAGAGATCCCATATGGCTTCTATCTTCATAGAAACTTTGTTGTGTACCATTAGTTGAACAGTTCTTCAAGAGAGGAG : 1323
R   NSFTV_161  : CATATAAATTGTCAATGATCCGAAAGTAAGCCATCATCCAGGTTCTCTTCATCATCATGGTGCAAAACACAGTCCATAGACCAATCACAAATCCTATGCTCATCCCGAGGTAGAGAGATCCCATATGGCTTCTATCTTCATAGAAACTTTGTTGTGTACCATTAGTTGAACAGTTCTTCAAGAGAGGAG : 1323
R   NSFTV_171  : CATATAAATTGTCAATGATCCGAAAGTAAGCCATCATCCAGGTTCTCTTCATCATCATGGTGCAAAACACAGTCCATAGACCAATCACAAATCCTATGCTCATCCCGAGGTAGAGAGATCCCATATGGCTTCTATCTTCATAGAAACTTTGTTGTGTACCATTAGTTGAACAGTTCTTCAAGAGAGGAG : 1323
R   NSFTV_209  : CATATAAATTGTCAATGATCCGAAAGTAAGCCATCATCCAGGTTCTCTTCATCATCATGGTGCAAAACACAGTCCATAGACCAATCACAAATCCTATGCTCATCCCGAGGTAGAGAGATCCCATATGGCTTCTATCTTCATAGAAACTTTGTTGTGTACCATTAGTTGAACAGTTCTTCAAGAGAGGAG : 1323
R   NSFTV_313  : CATATAAATTGTCAATGATCCGAAAGTAAGCCATCATCCAGGTTCTCTTCATCATCATGGTGCAAAACACAGTCCATAGACCAATCACAAATCCTATGCTCATCCCGAGGTAGAGAGATCCCATATGGCTTCTATCTTCATAGAAACTTTGTTGTGTACCATTAGTTGAACAGTTCTTCAAGAGAGGAG : 1323
R   NSFTV_337  : CATATAAATTGTCAATGATCCGAAAGTAAGCCATCATCCAGGTTCTCTTCATCATCATGGTGCAAAACACAGTCCATAGACCAATCACAAATCCTATGCTCATCCCGAGGTAGAGAGATCCCATATGGCTTCTATCTTCATAGAAACTTTGTTGTGTACCATTAGTTGAACAGTTCTTCAAGAGAGGAG : 1323
S   Nipponbare : CATATAAATTGTCAATGATCCGAAAGTAAGCCATCATCCAGGTTCTCTTCATCATCATGGTGCAAAACACAGTCCATAGACCAATCACAAATCCTATGCTCATCCCGAGGTAGAGAGATCTCATATGGCTTCTATCTTCATAGAAACTTTGTTGTGTACCATTAGTTGAACAGTTCTTCAAGAGAGGAG : 1323
S   NSFTV_17   : CATATAAATTGTCAATGATCCGAAAGTAAGCCATCATCCAGGTTCTCTTCATCATCATGGTGCAAAACACAGTCCATAGACCAATCACAAATCCTATGCTCATCCCGAGGTAGAGAGATCTCATATGGCTTCTATCTTCATAGAAACTTTGTTGTGTACCATTAGTTGAACAGTTCTTCAAGAGAGGAG : 1323
S   NSFTV_66   : CATATAAATTGTCAATGATCCGAAAGTAAGCCATCATCCAGGTTCTCTTCATCATCATGGTGCAAAACACAGTCCATAGACCAATCACAAATCCTATGCTCATCCCGAGGTAGAGAGATCTCATATGGCTTCTATCTTCATAGAAACTTTGTTGTGTACCATTAGTTGAACAGTTCTTCAAGAGAGGAG : 1323
S   NSFTV_110  : CATATAAATTGTCAATGATCCGAAAGTAAGCCATCATCCAGGTTCTCTTCATCATCATGGTGCAAAACACAGTCCATAGACCAATCACAAATCCTATGCTCATCCCGAGGTAGAGAGATCTCATATGGCTTCTATCTTCATAGAAACTTTGTTGTGTACCATTAGTTGAACAGTTCTTCAAGAGAGGAG : 1323
S   NSFTV_138  : CATATAAATTGTCAATGATCCGAAAGTAAGCCATCATCCAGGTTCTCTTCATCATCATGGTGCAAAACACAGTCCATAGACCAATCACAAATCCTATGCTCATCCCGAGGTAGAGAGATCTCATATGGCTTCTATCTTCATAGAAACTTTGTTGTGTACCATTAGTTGAACAGTTCTTCAAGAGAGGAG : 1323
S   NSFTV_145  : CATATAAATTGTCAATGATCCGAAAGTAAGCCATCATCCAGGTTCTCTTCATCATCATGGTGCAAAACACAGTCCATAGACCAATCACAAATCCTATGCTCATCCCGAGGTAGAGAGATCTCATATGGCTTCTATCTTCATAGAAACTTTGTTGTGTACCATTAGTTGAACAGTTCTTCAAGAGAGGAG : 1323
S   NSFTV_252  : CATATAAATTGTCAATGATCCGAAAGTAAGCCATCATCCAGGTTCTCTTCATCATCATGGTGCAAAACACAGTCCATAGACCAATCACAAATCCTATGCTCATCCCGAGGTAGAGAGATCTCATATGGCTTCTATCTTCATAGAAACTTTGTTGTGTACCATTAGTTGAACAGTTCTTCAAGAGAGGAG : 1323
S   NSFTV_255  : CATATAAATTGTCAATGATCCGAAAGTAAGCCATCATCCAGGTTCTCTTCATCATCATGGTGCAAAACACAGTCCATAGACCAATCACAAATCCTATGCTCATCCCGAGGTAGAGAGATCTCATATGGCTTCTATCTTCATAGAAACTTTGTTGTGTACCATTAGTTGAACAGTTCTTCAAGAGAGGAG : 1323
S   NSFTV_304  : CATATAAATTGTCAATGATCCGAAAGTAAGCCATCATCCAGGTTCTCTTCATCATCATGGTGCAAAACACAGTCCATAGACCAATCACAAATCCTATGCTCATCCCGAGGTAGAGAGATCTCATATGGCTTCTATCTTCATAGAAACTTTGTTGTGTACCATTAGTTGAACAGTTCTTCAAGAGAGGAG : 1323
                 CATATAAATTGTCAATGATCCGAAAGTAAGCCATCATCCAGGTTCTCTTCATCATCATGGTGCAAAACACAGTCCATAGACCAATCACAAATCCTATGCTCATCCCGAGGTAGAGAGATC CATATGGCTTCTATCTTCATAGAAACTTTGTTGTGTACCATTAGTTGAACAGTTCTTCAAGAGAGGAG
```

```
qBK1.7                                                                                                                                                        Os01g0601625
haplotype          *        1340         *        1360         *        1380         *        1400         *        1420         *        1440         *        1460         *        1480         *        1500         *
R   IR64       : GCCCACAAAGACCAGGATTTCCAACATATATATAAATCTGGTCATCGAGTGCTTGTAGCTGTGATCCTGATGGTATTGTGCCTGATAAATTGTTGTATGATAGGTTCAAGTGGCTTAGAGAAGTTAGAGCTGACAAACTTGGGGGGATTTCACCAGATAATTCATTGTAGGACAAGTCAAGGGATTCCA : 1512
R   NSFTV_18   : GCCCACAAAGACCAGGATTTCCAACATATATATAAATCTGGTCATCGAGTGCTTGTAGCTGTGATCCTGATGGTATTGTGCCTGATAAATTGTTGTATGATAGGTTCAAGTGGCTTAGAGAAGTTAGAGCTGACAAACTTGGGGGGATTTCACCAGATAATTCATTGTAGGACAAGTCAAGGGATTCCA : 1512
R   NSFTV_19   : GCCCACAAAGACCAGGATTTCCAACATATATATAAATCTGGTCATCGAGTGCTTGTAGCTGTGATCCTGATGGTATTGTGCCTGATAAATTGTTGTATGATAGGTTCAAGTGGCTTAGAGAAGTTAGAGCTGACAAACTTGGGGGGATTTCACCAGATAATTCATTGTAGGACAAGTCAAGGGATTCCA : 1512
R   NSFTV_74   : GCCCACAAAGACCAGGATTTCCAACATATATATAAATCTGGTCATCGAGTGCTTGTAGCTGTGATCCTGATGGTATTGTGCCTGATAAATTGTTGTATGATAGGTTCAAGTGGCTTAGAGAAGTTAGAGCTGACAAACTTGGGGGGATTTCACCAGATAATTCATTGTAGGACAAGTCAAGGGATTCCA : 1512
R   NSFTV_85   : GCCCACAAAGACCAGGATTTCCAACATATATATAAATCTGGTCATCGAGTGCTTGTAGCTGTGATCCTGATGGTATTGTGCCTGATAAATTGTTGTATGATAGGTTCAAGTGGCTTAGAGAAGTTAGAGCTGACAAACTTGGGGGGATTTCACCAGATAATTCATTGTAGGACAAGTCAAGGGATTCCA : 1512
R   NSFTV_137  : GCCCACAAAGACCAGGATTTCCAACATATATATAAATCTGGTCATCGAGTGCTTGTAGCTGTGATCCTGATGGTATTGTGCCTGATAAATTGTTGTATGATAGGTTCAAGTGGCTTAGAGAAGTTAGAGCTGACAAACTTGGGGGGATTTCACCAGATAATTCATTGTAGGACAAGTCAAGGGATTCCA : 1512
R   NSFTV_161  : GCCCACAAAGACCAGGATTTCCAACATATATATAAATCTGGTCATCGAGTGCTTGTAGCTGTGATCCTGATGGTATTGTGCCTGATAAATTGTTGTATGATAGGTTCAAGTGGCTTAGAGAAGTTAGAGCTGACAAACTTGGGGGGATTTCACCAGATAATTCATTGTAGGACAAGTCAAGGGATTCCA : 1512
R   NSFTV_171  : GCCCACAAAGACCAGGATTTCCAACATATATATAAATCTGGTCATCGAGTGCTTGTAGCTGTGATCCTGATGGTATTGTGCCTGATAAATTGTTGTATGATAGGTTCAAGTGGCTTAGAGAAGTTAGAGCTGACAAACTTGGGGGGATTTCACCAGATAATTCATTGTAGGACAAGTCAAGGGATTCCA : 1512
R   NSFTV_209  : GCCCACAAAGACCAGGATTTCCAACATATATATAAATCTGGTCATCGAGTGCTTGTAGCTGTGATCCTGATGGTATTGTGCCTGATAAATTGTTGTATGATAGGTTCAAGTGGCTTAGAGAAGTTAGAGCTGACAAACTTGGGGGGATTTCACCAGATAATTCATTGTAGGACAAGTCAAGGGATTCCA : 1512
R   NSFTV_313  : GCCCACAAAGACCAGGATTTCCAACATATATATAAATCTGGTCATCGAGTGCTTGTAGCTGTGATCCTGATGGTATTGTGCCTGATAAATTGTTGTATGATAGGTTCAAGTGGCTTAGAGAAGTTAGAGCTGACAAACTTGGGGGGATTTCACCAGATAATTCATTGTAGGACAAGTCAAGGGATTCCA : 1512
R   NSFTV_337  : GCCCACAAAGACCAGGATTTCCAACATATATATAAATCTGGTCATCGAGTGCTTGTAGCTGTGATCCTGATGGTATTGTGCCTGATAAATTGTTGTATGATAGGTTCAAGTGGCTTAGAGAAGTTAGAGCTGACAAACTTGGGGGGATTTCACCAGATAATTCATTGTAGGACAAGTCAAGGGATTCCA : 1512
S   Nipponbare : GCCCACAAAGACCAGGATTTCCAACATATATATAAATCTGGTCATCGAGTGCTTGTAGCTGTGATCCTGATGGTATTGTGCCTGATAAATTGTTGTATGATAGGTTCAAGTGGCTTAGAGAAGTTAGAGCTGACAAACTTGGGGGGATTTCACCAGATAATTCATTGTAGGACAAGTCAAGGGATTCCA : 1512
S   NSFTV_17   : GCCCACAAAGACCAGGATTTCCAACATATATATAAATCTGGTCATCGAGTGCTTGTAGCTGTGATCCTGATGGTATTGTGCCTGATAAATTGTTGTATGATAGGTTCAAGTGGCTTAGAGAAGTTAGAGCTGACAAACTTGGGGGGATTTCACCAGATAATTCATTGTAGGACAAGTCAAGGGATTCCA : 1512
S   NSFTV_66   : GCCCACAAAGACCAGGATTTCCAACATATATATAAATCTGGTCATCGAGTGCTTGTAGCTGTGATCCTGATGGTATTGTGCCTGATAAATTGTTGTATGATAGGTTCAAGTGGCTTAGAGAAGTTAGAGCTGACAAACTTGGGGGGATTTCACCAGATAATTCATTGTAGGACAAGTCAAGGGATTCCA : 1512
S   NSFTV_110  : GCCCACAAAGACCAGGATTTCCAACATATATATAAATCTGGTCATCGAGTGCTTGTAGCTGTGATCCTGATGGTATTGTGCCTGATAAATTGTTGTATGATAGGTTCAAGTGGCTTAGAGAAGTTAGAGCTGACAAACTTGGGGGGATTTCACCAGATAATTCATTGTAGGACAAGTCAAGGGATTCCA : 1512
S   NSFTV_138  : GCCCACAAAGACCAGGATTTCCAACATATATATAAATCTGGTCATCGAGTGCTTGTAGCTGTGATCCTGATGGTATTGTGCCTGATAAATTGTTGTATGATAGGTTCAAGTGGCTTAGAGAAGTTAGAGCTGACAAACTTGGGGGGATTTCACCAGATAATTCATTGTAGGACAAGTCAAGGGATTCCA : 1512
S   NSFTV_145  : GCCCACAAAGACCAGGATTTCCAACATATATATAAATCTGGTCATCGAGTGCTTGTAGCTGTGATCCTGATGGTATTGTGCCTGATAAATTGTTGTATGATAGGTTCAAGTGGCTTAGAGAAGTTAGAGCTGACAAACTTGGGGGGATTTCACCAGATAATTCATTGTAGGACAAGTCAAGGGATTCCA : 1512
S   NSFTV_252  : GCCCACAAAGACCAGGATTTCCAACATATATATAAATCTGGTCATCGAGTGCTTGTAGCTGTGATCCTGATGGTATTGTGCCTGATAAATTGTTGTATGATAGGTTCAAGTGGCTTAGAGAAGTTAGAGCTGACAAACTTGGGGGGATTTCACCAGATAATTCATTGTAGGACAAGTCAAGGGATTCCA : 1512
S   NSFTV_255  : GCCCACAAAGACCAGGATTTCCAACATATATATAAATCTGGTCATCGAGTGCTTGTAGCTGTGATCCTGATGGTATTGTGCCTGATAAATTGTTGTATGATAGGTTCAAGTGGCTTAGAGAAGTTAGAGCTGACAAACTTGGGGGGATTTCACCAGATAATTCATTGTAGGACAAGTCAAGGGATTCCA : 1512
S   NSFTV_304  : GCCCACAAAGACCAGGATTTCCAACATATATATAAATCTGGTCATCGAGTGCTTGTAGCTGTGATCCTGATGGTATTGTGCCTGATAAATTGTTGTATGATAGGTTCAAGTGGCTTAGAGAAGTTAGAGCTGACAAACTTGGGGGGATTTCACCAGATAATTCATTGTAGGACAAGTCAAGGGATTCCA : 1512
                 GCCCACAAAGACCAGGATTTCCAACATATATATAAATCTGGTCATCGAGTGCTTGTAGCTGTGATCCTGATGGTATTGTGCCTGATAAATTGTTGTATGATAGGTTCAAGTGGCTTAGAGAAGTTAGAGCTGACAAACTTGGGGGGATTTCACCAGATAATTCATTGTAGGACAAGTCAAGGGATTCCA
```

```
qBK1.7                                                                                                                                                        Os01g0601625
haplotype         1520         *        1540         *        1560         *        1580         *        1600         *        1620         *        1640         *        1660         *        1680         *        1700
R   IR64       : ACTGCTTCAAATCACCAATTTGATCATGGATTGTTCCACTGAACTGATTACTTGATAAATTTAAATTGGTAAGTCCAATAAGTAAATGTATTTCTTCTGGAATATGCGCTGTTAACTTGTTACATGAAAAATCAAGATTAACCACCTGATTGTAGATTTCAAAGGTGTAGTCACGCTGTTGATCTTTCG : 1701
R   NSFTV_18   : ACTGCTTCAAATCACCAATTTGATCATGGATTGTTCCACTGAACTGATTACTTGATAAATTTAAATTGGTAAGTCCAATAAGTAAATGTATTTCTTCTGGAATATGCGCTGTTAACTTGTTACATGAAAAATCAAGATTAACCACCTGATTGTAGATTTCAAAGGTGTAGTCACGCTGTTGATCTTTCG : 1701
R   NSFTV_19   : ACTGCTTCAAATCACCAATTTGATCATGGATTGTTCCACTGAACTGATTACTTGATAAATTTAAATTGGTAAGTCCAATAAGTAAATGTATTTCTTCTGGAATATGCGCTGTTAACTTGTTACATGAAAAATCAAGATTAACCACCTGATTGTAGATTTCAAAGGTGTAGTCACGCTGTTGATCTTTCG : 1701
R   NSFTV_74   : ACTGCTTCAAATCACCAATTTGATCATGGATTGTTCCACTGAACTGATTACTTGATAAATTTAAATTGGTAAGTCCAATAAGTAAATGTATTTCTTCTGGAATATGCGCTGTTAACTTGTTACATGAAAAATCAAGATTAACCACCTGATTGTAGATTTCAAAGGTGTAGTCACGCTGTTGATCTTTCG : 1701
R   NSFTV_85   : ACTGCTTCAAATCACCAATTTGATCATGGATTGTTCCACTGAACTGATTACTTGATAAATTTAAATTGGTAAGTCCAATAAGTAAATGTATTTCTTCTGGAATATGCGCTGTTAACTTGTTACATGAAAAATCAAGATTAACCACCTGATTGTAGATTTCAAAGGTGTAGTCACGCTGTTGATCTTTCG : 1701
R   NSFTV_137  : ACTGCTTCAAATCACCAATTTGATCATGGATTGTTCCACTGAACTGATTACTTGATAAATTTAAATTGGTAAGTCCAATAAGTAAATGTATTTCTTCTGGAATATGCGCTGTTAACTTGTTACATGAAAAATCAAGATTAACCACCTGATTGTAGATTTCAAAGGTGTAGTCACGCTGTTGATCTTTCG : 1701
R   NSFTV_161  : ACTGCTTCAAATCACCAATTTGATCATGGATTGTTCCACTGAACTGATTACTTGATAAATTTAAATTGGTAAGTCCAATAAGTAAATGTATTTCTTCTGGAATATGCGCTGTTAACTTGTTACATGAAAAATCAAGATTAACCACCTGATTGTAGATTTCAAAGGTGTAGTCACGCTGTTGATCTTTCG : 1701
R   NSFTV_171  : ACTGCTTCAAATCACCAATTTGATCATGGATTGTTCCACTGAACTGATTACTTGATAAATTTAAATTGGTAAGTCCAATAAGTAAATGTATTTCTTCTGGAATATGCGCTGTTAACTTGTTACATGAAAAATCAAGATTAACCACCTGATTGTAGATTTCAAAGGTGTAGTCACGCTGTTGATCTTTCG : 1701
R   NSFTV_209  : ACTGCTTCAAATCACCAATTTGATCATGGATTGTTCCACTGAACTGATTACTTGATAAATTTAAATTGGTAAGTCCAATAAGTAAATGTATTTCTTCTGGAATATGCGCTGTTAACTTGTTACATGAAAAATCAAGATTAACCACCTGATTGTAGATTTCAAAGGTGTAGTCACGCTGTTGATCTTTCG : 1701
R   NSFTV_313  : ACTGCTTCAAATCACCAATTTGATCATGGATTGTTCCACTGAACTGATTACTTGATAAATTTAAATTGGTAAGTCCAATAAGTAAATGTATTTCTTCTGGAATATGCGCTGTTAACTTGTTACATGAAAAATCAAGATTAACCACCTGATTGTAGATTTCAAAGGTGTAGTCACGCTGTTGATCTTTCG : 1701
R   NSFTV_337  : ACTGCTTCAAATCACCAATTTGATCATGGATTGTTCCACTGAACTGATTACTTGATAAATTTAAATTGGTAAGTCCAATAAGTAAATGTATTTCTTCTGGAATATGCGCTGTTAACTTGTTACATGAAAAATCAAGATTAACCACCTGATTGTAGATTTCAAAGGTGTAGTCACGCTGTTGATCTTTCG : 1701
S   Nipponbare : ACTGCTTCAAATCACCAATTTGATCATGGATTGTTCCACTGAACTGATTACTTGATAAATTTAAATTGGTAAGTCCAATAAGTAAATGTATTTCTTCTGGAATATGCCCTGTTAACTTGTTACATGAAAAATCAAGATTAACCACCTGATTGTAGATTTCAAAGGTGTAGTCACGCTGTTGATCTTTCG : 1701
S   NSFTV_17   : ACTGCTTCAAATCACCAATTTGATCATGGATTGTTCCACTGAACTGATTACTTGATAAATTTAAATTGGTAAGTCCAATAAGTAAATGTATTTCTTCTGGAATATGCCCTGTTAACTTGTTACATGAAAAATCAAGATTAACCACCTGATTGTAGATTTCAAAGGTGTAGTCACGCTGTTGATCTTTCG : 1701
S   NSFTV_66   : ACTGCTTCAAATCACCAATTTGATCATGGATTGTTCCACTGAACTGATTACTTGATAAATTTAAATTGGTAAGTCCAATAAGTAAATGTATTTCTTCTGGAATATGCCCTGTTAACTTGTTACATGAAAAATCAAGATTAACCACCTGATTGTAGATTTCAAAGGTGTAGTCACGCTGTTGATCTTTCG : 1701
S   NSFTV_110  : ACTGCTTCAAATCACCAATTTGATCATGGATTGTTCCACTGAACTGATTACTTGATAAATTTAAATTGGTAAGTCCAATAAGTAAATGTATTTCTTCTGGAATATGCCCTGTTAACTTGTTACATGAAAAATCAAGATTAACCACCTGATTGTAGATTTCAAAGGTGTAGTCACGCTGTTGATCTTTCG : 1701
S   NSFTV_138  : ACTGCTTCAAATCACCAATTTGATCATGGATTGTTCCACTGAACTGATTACTTGATAAATTTAAATTGGTAAGTCCAATAAGTAAATGTATTTCTTCTGGAATATGCCCTGTTAACTTGTTACATGAAAAATCAAGATTAACCACCTGATTGTAGATTTCAAAGGTGTAGTCACGCTGTTGATCTTTCG : 1701
S   NSFTV_145  : ACTGCTTCAAATCACCAATTTGATCATGGATTGTTCCACTGAACTGATTACTTGATAAATTTAAATTGGTAAGTCCAATAAGTAAATGTATTTCTTCTGGAATATGCCCTGTTAACTTGTTACATGAAAAATCAAGATTAACCACCTGATTGTAGATTTCAAAGGTGTAGTCACGCTGTTGATCTTTCG : 1701
S   NSFTV_252  : ACTGCTTCAAATCACCAATTTGATCATGGATTGTTCCACTGAACTGATTACTTGATAAATTTAAATTGGTAAGTCCAATAAGTAAATGTATTTCTTCTGGAATATGCCCTGTTAACTTGTTACATGAAAAATCAAGATTAACCACCTGATTGTAGATTTCAAAGGTGTAGTCACGCTGTTGATCTTTCG : 1701
S   NSFTV_255  : ACTGCTTCAAATCACCAATTTGATCATGGATTGTTCCACTGAACTGATTACTTGATAAATTTAAATTGGTAAGTCCAATAAGTAAATGTATTTCTTCTGGAATATGCCCTGTTAACTTGTTACATGAAAAATCAAGATTAACCACCTGATTGTAGATTTCAAAGGTGTAGTCACGCTGTTGATCTTTCG : 1701
S   NSFTV_304  : ACTGCTTCAAATCACCAATTTGATCATGGATTGTTCCACTGAACTGATTACTTGATAAATTTAAATTGGTAAGTCCAATAAGTAAATGTATTTCTTCTGGAATATGCCCTGTTAACTTGTTACATGAAAAATCAAGATTAACCACCTGATTGTAGATTTCAAAGGTGTAGTCACGCTGTTGATCTTTCG : 1701
                 ACTGCTTCAAATCACCAATTTGATCATGGATTGTTCCACTGAACTGATTACTTGATAAATTTAAATTGGTAAGTCCAATAAGTAAATGTATTTCTTCTGGAATATGC CTGTTAACTTGTTACATGAAAAATCAAGATTAACCACCTGATTGTAGATTTCAAAGGTGTAGTCACGCTGTTGATCTTTCG
```

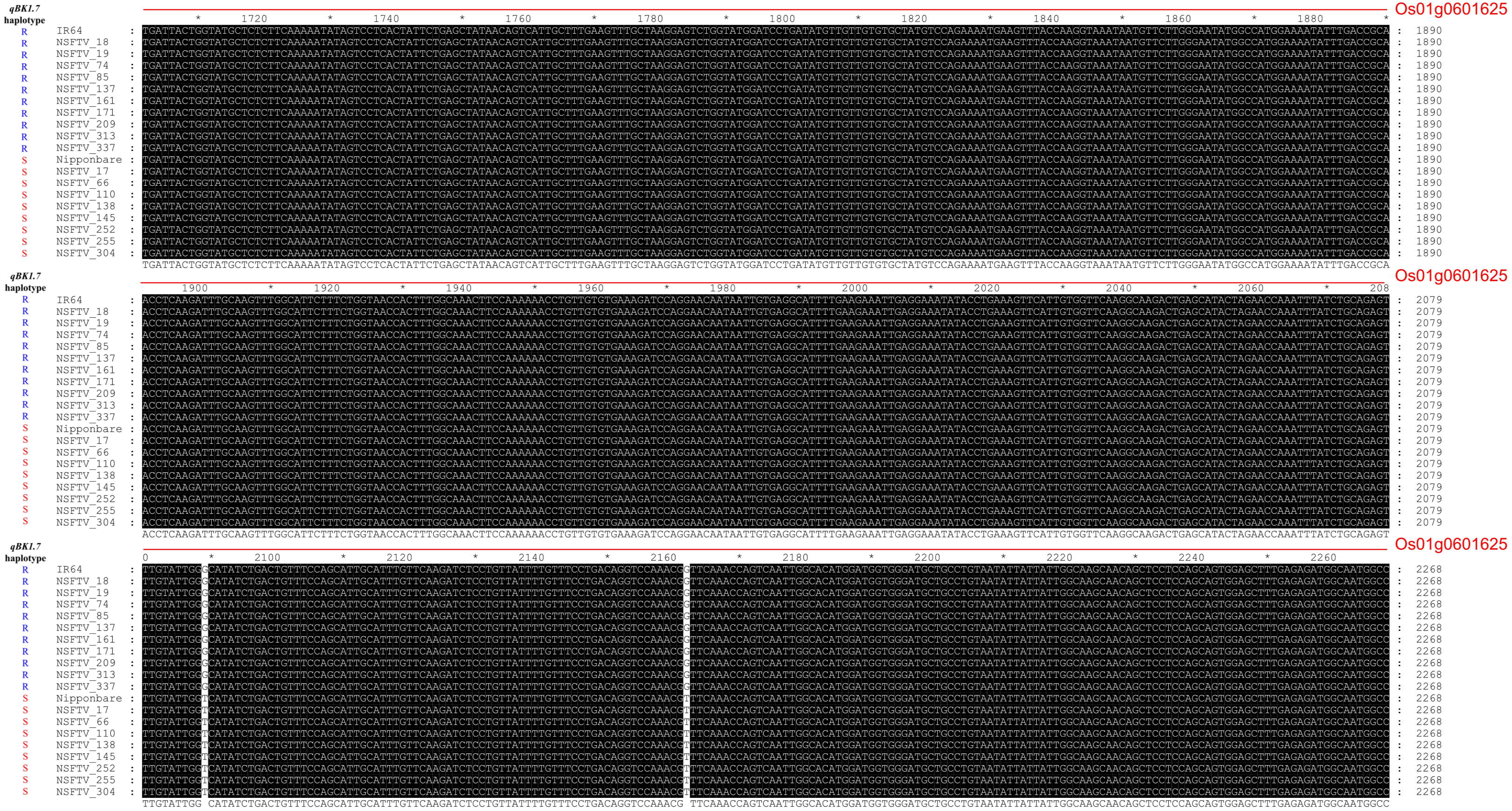
qBK1.7 haplotype
Os01g0601625
R IR64
R NSFTV_18
R NSFTV_19
R NSFTV_74
R NSFTV_85
R NSFTV_137
R NSFTV_161
R NSFTV_171
R NSFTV_209
R NSFTV_313
R NSFTV_337
S Nipponbare
S NSFTV_17
S NSFTV_66
S NSFTV_110
S NSFTV_138
S NSFTV_145
S NSFTV_252
S NSFTV_255
S NSFTV_304

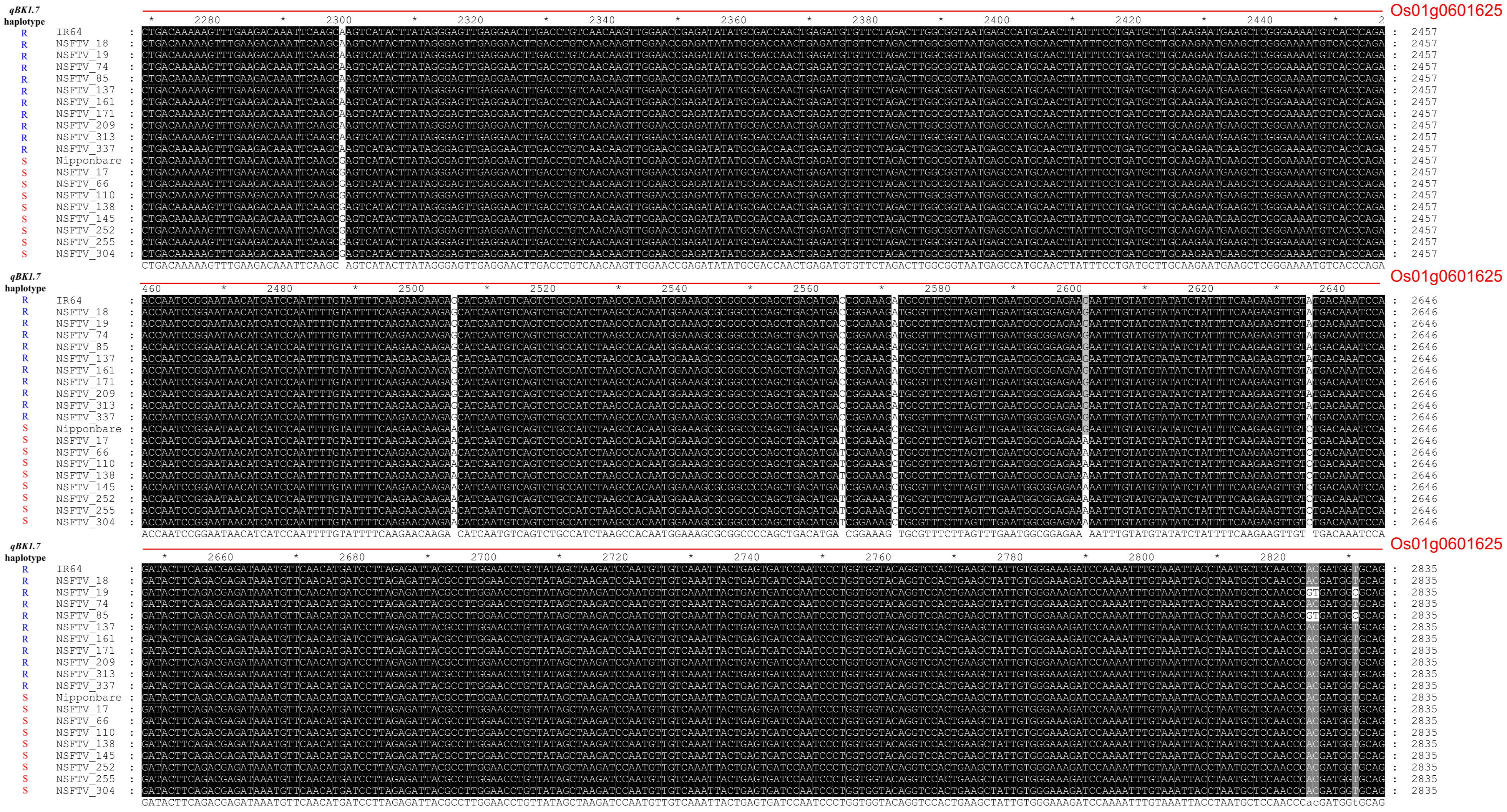
qBK1.7 haplotype
Os01g0601625

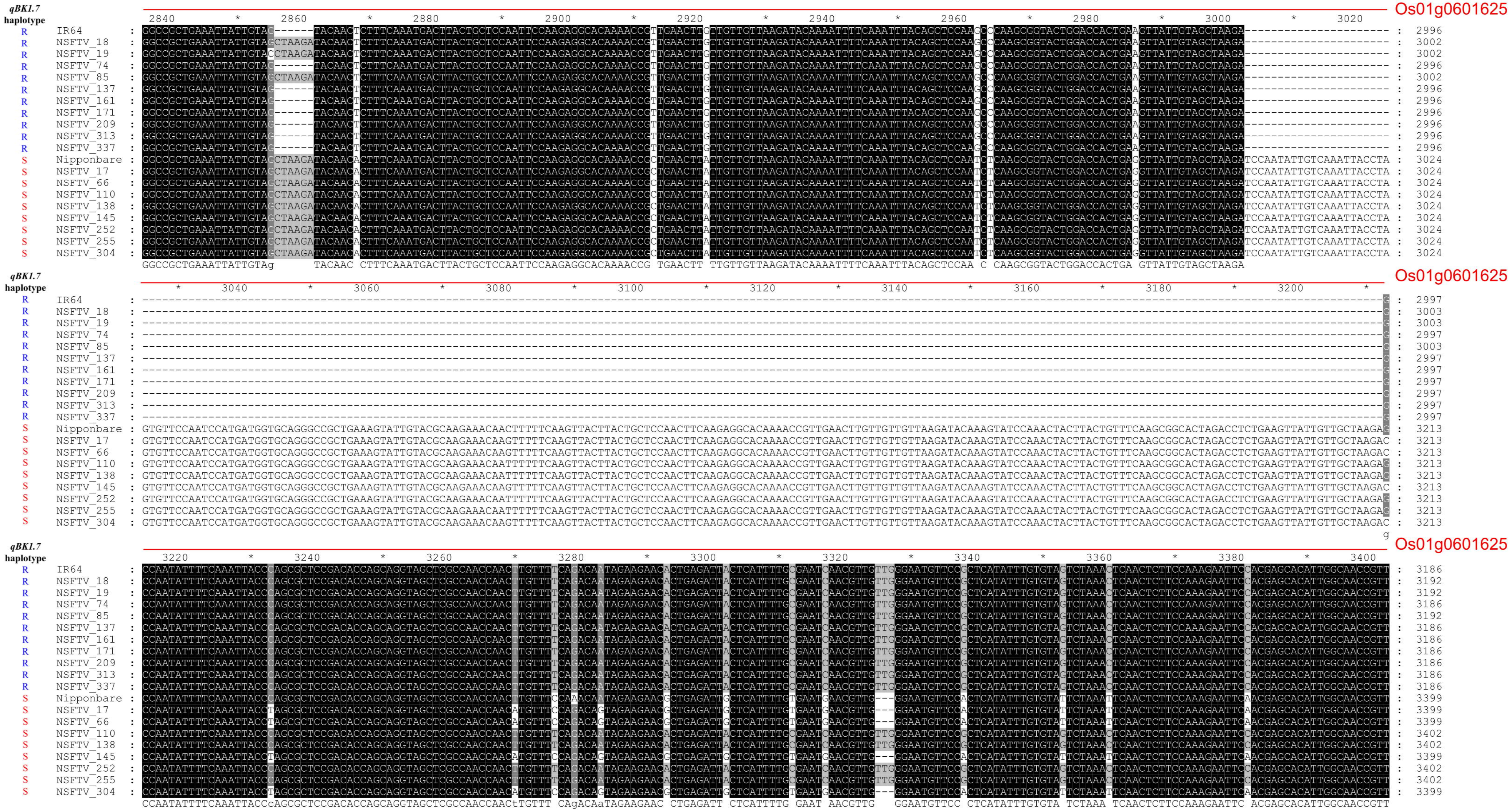
qBK1.7 haplotype
Os01g0601625

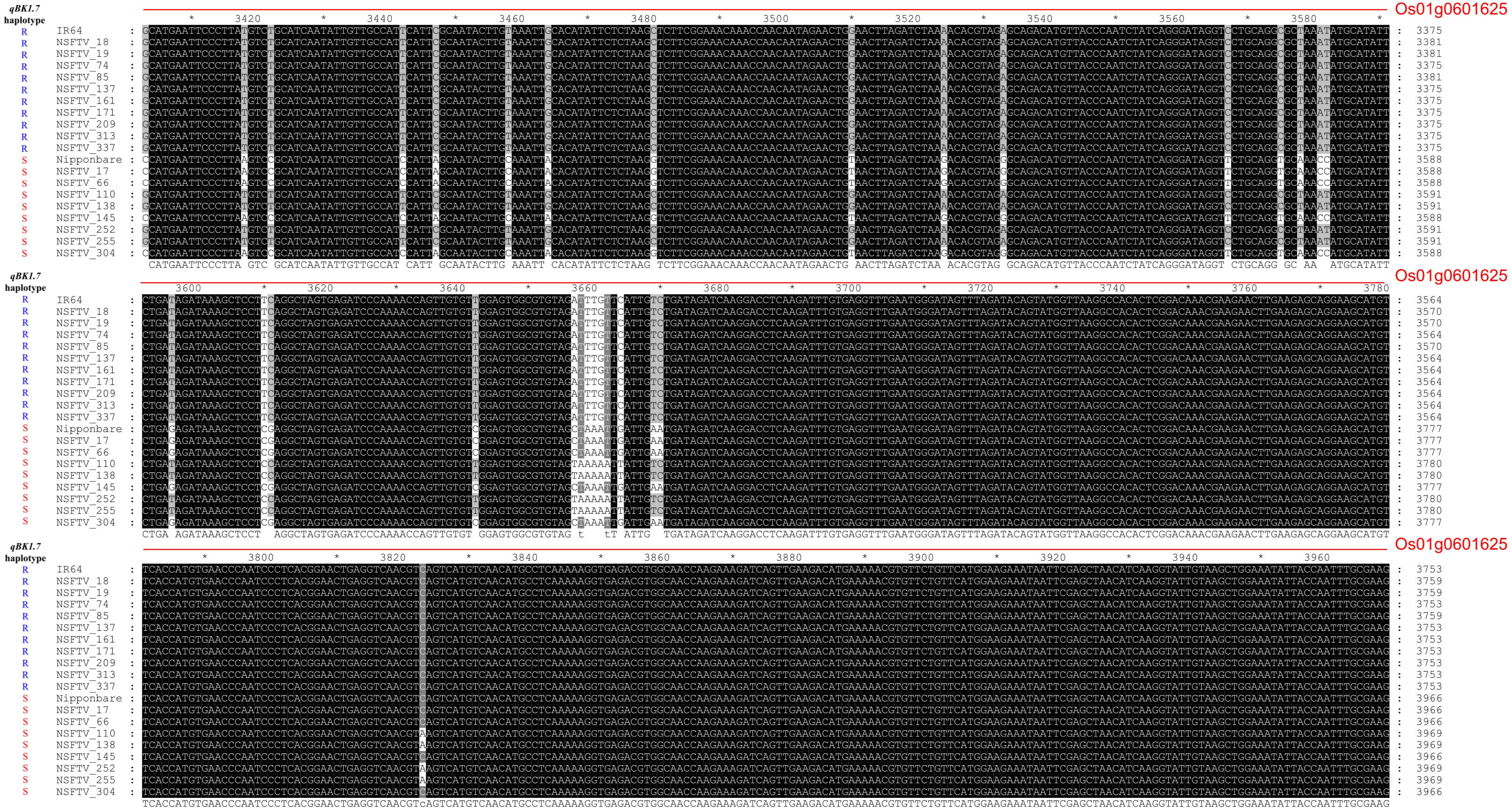
qBK1.7 haplotype
Os01g0601625
R
S
IR64
NSFTV_18
NSFTV_19
NSFTV_74
NSFTV_85
NSFTV_137
NSFTV_161
NSFTV_171
NSFTV_209
NSFTV_313
NSFTV_337
Nipponbare
NSFTV_17
NSFTV_66
NSFTV_110
NSFTV_138
NSFTV_145
NSFTV_252
NSFTV_255
NSFTV_304

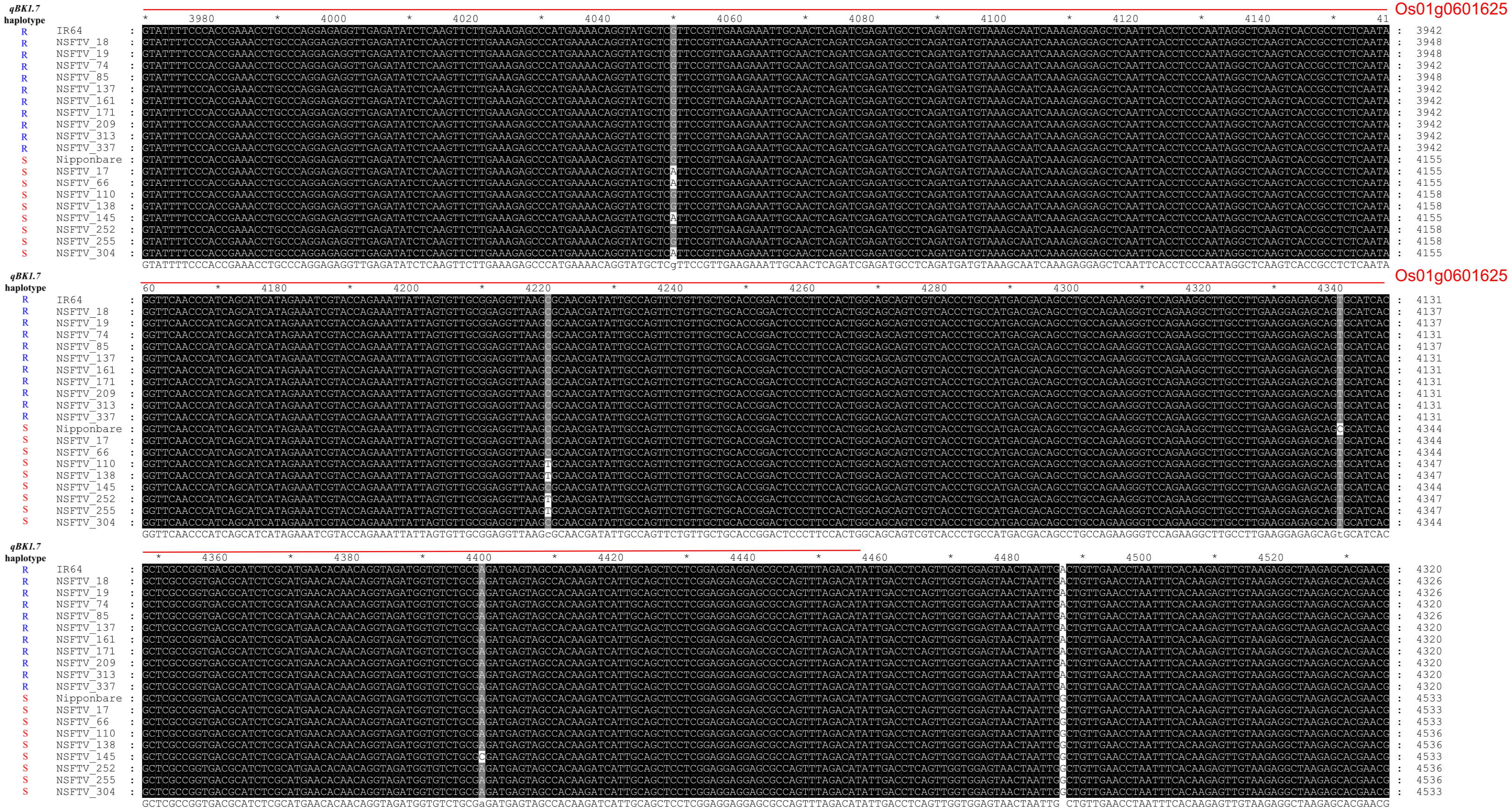
Os01g0601625
qBK1.7 haplotype
Os01g0601625
qBK1.7 haplotype
qBK1.7 haplotype

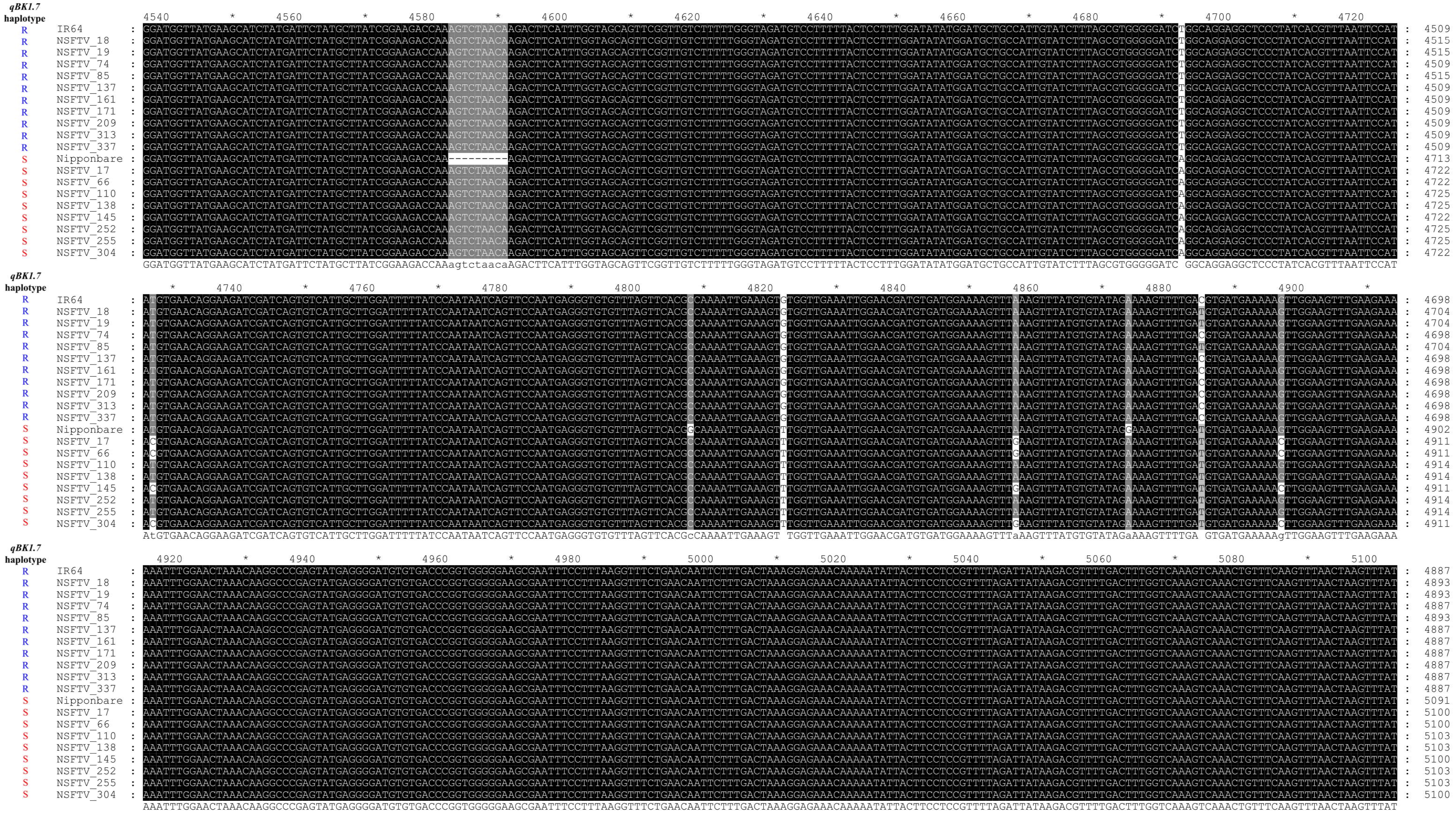
qBK1.7 haplotype
R IR64
R NSFTV_18
R NSFTV_19
R NSFTV_74
R NSFTV_85
R NSFTV_137
R NSFTV_161
R NSFTV_171
R NSFTV_209
R NSFTV_313
R NSFTV_337
S Nipponbare
S NSFTV_17
S NSFTV_66
S NSFTV_110
S NSFTV_138
S NSFTV_145
S NSFTV_252
S NSFTV_255
S NSFTV_304

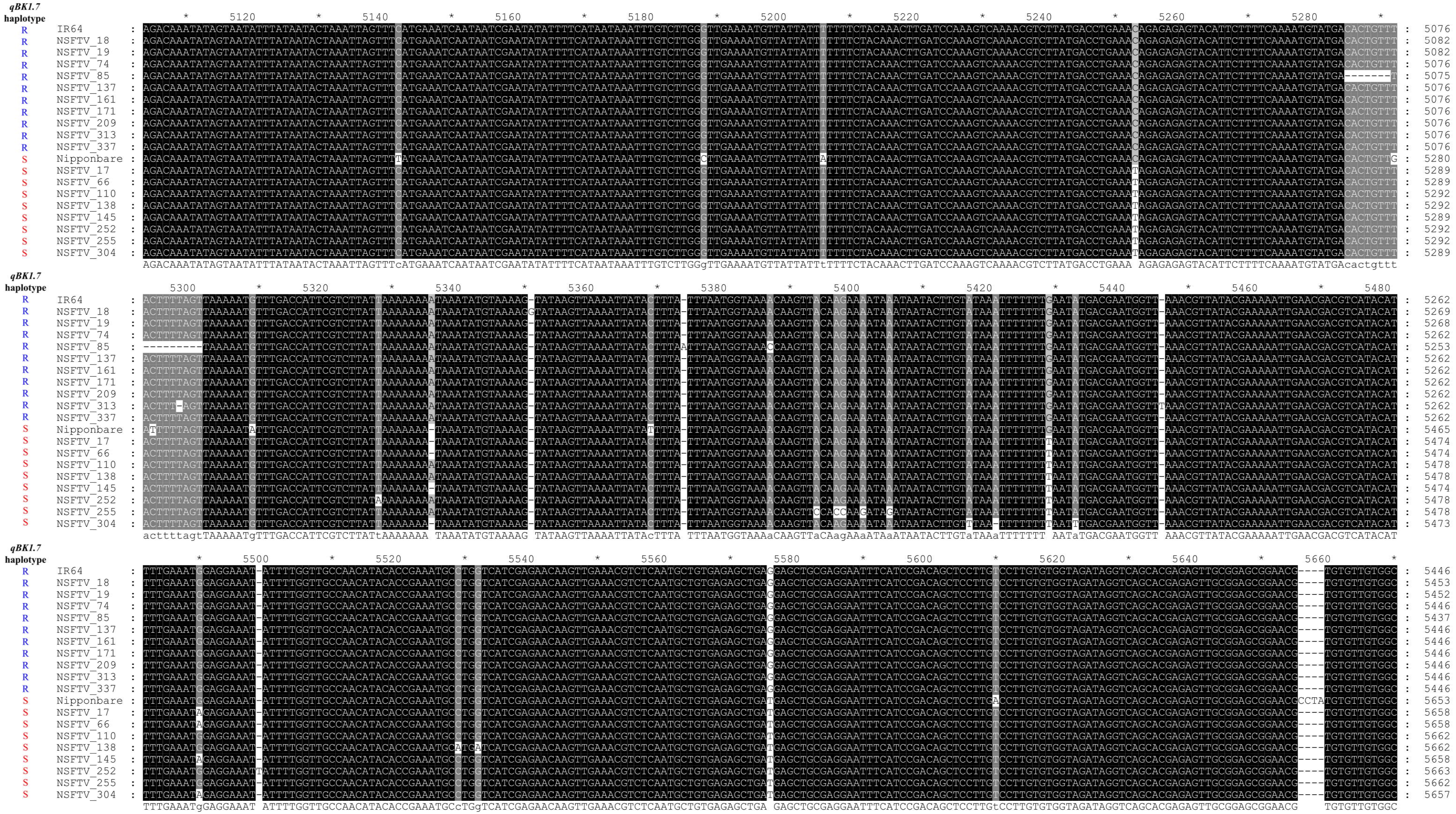
qBK1.7 haplotype
IR64
NSFTV_18
NSFTV_19
NSFTV_74
NSFTV_85
NSFTV_137
NSFTV_161
NSFTV_171
NSFTV_209
NSFTV_313
NSFTV_337
Nipponbare
NSFTV_17
NSFTV_66
NSFTV_110
NSFTV_138
NSFTV_145
NSFTV_252
NSFTV_255
NSFTV_304

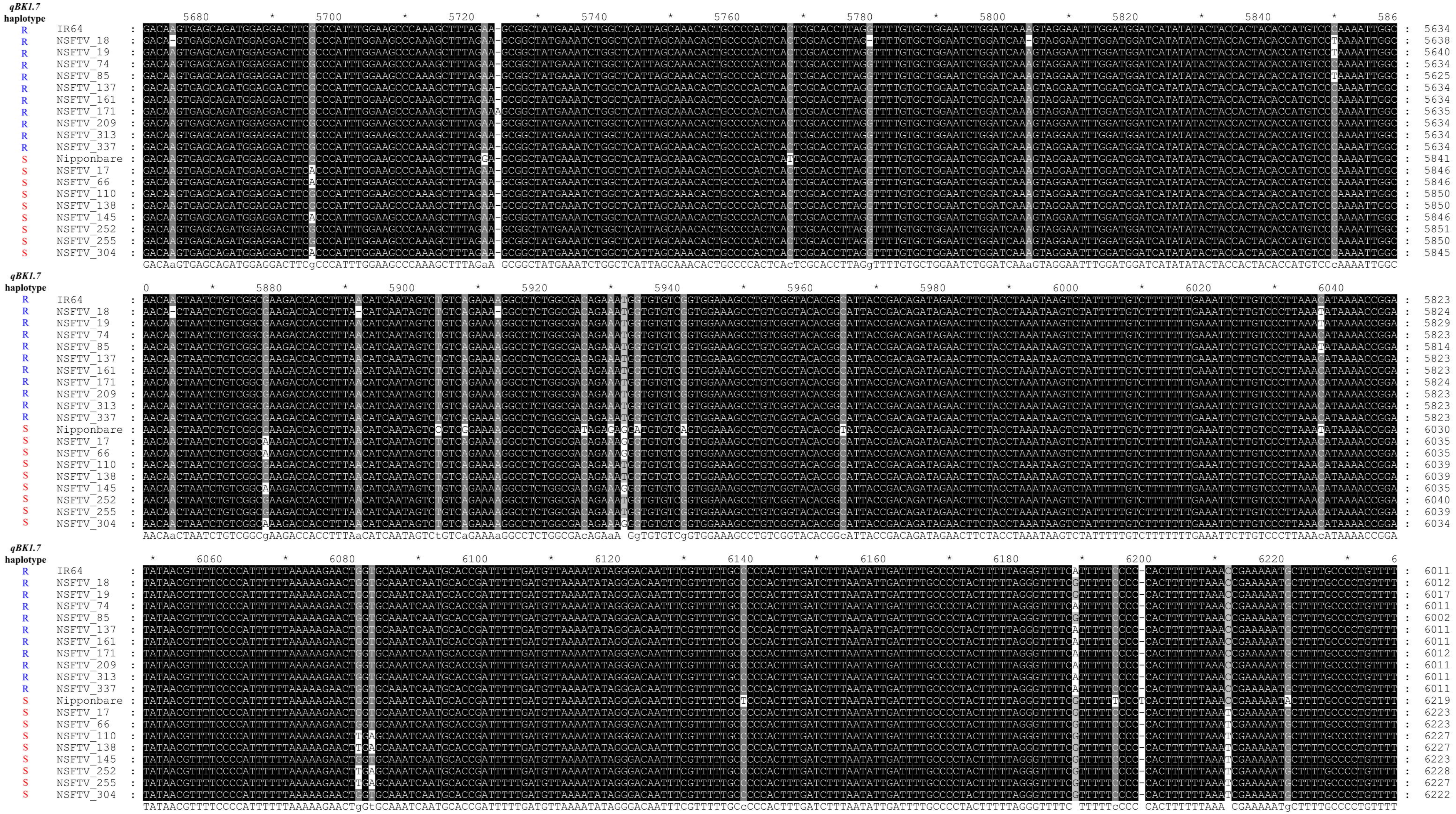

qBK1.7 haplotype
R IR64
R NSFTV_18
R NSFTV_19
R NSFTV_74
R NSFTV_85
R NSFTV_137
R NSFTV_161
R NSFTV_171
R NSFTV_209
R NSFTV_313
R NSFTV_337
S Nipponbare
S NSFTV_17
S NSFTV_66
S NSFTV_110
S NSFTV_138
S NSFTV_145
S NSFTV_252
S NSFTV_255
S NSFTV_304

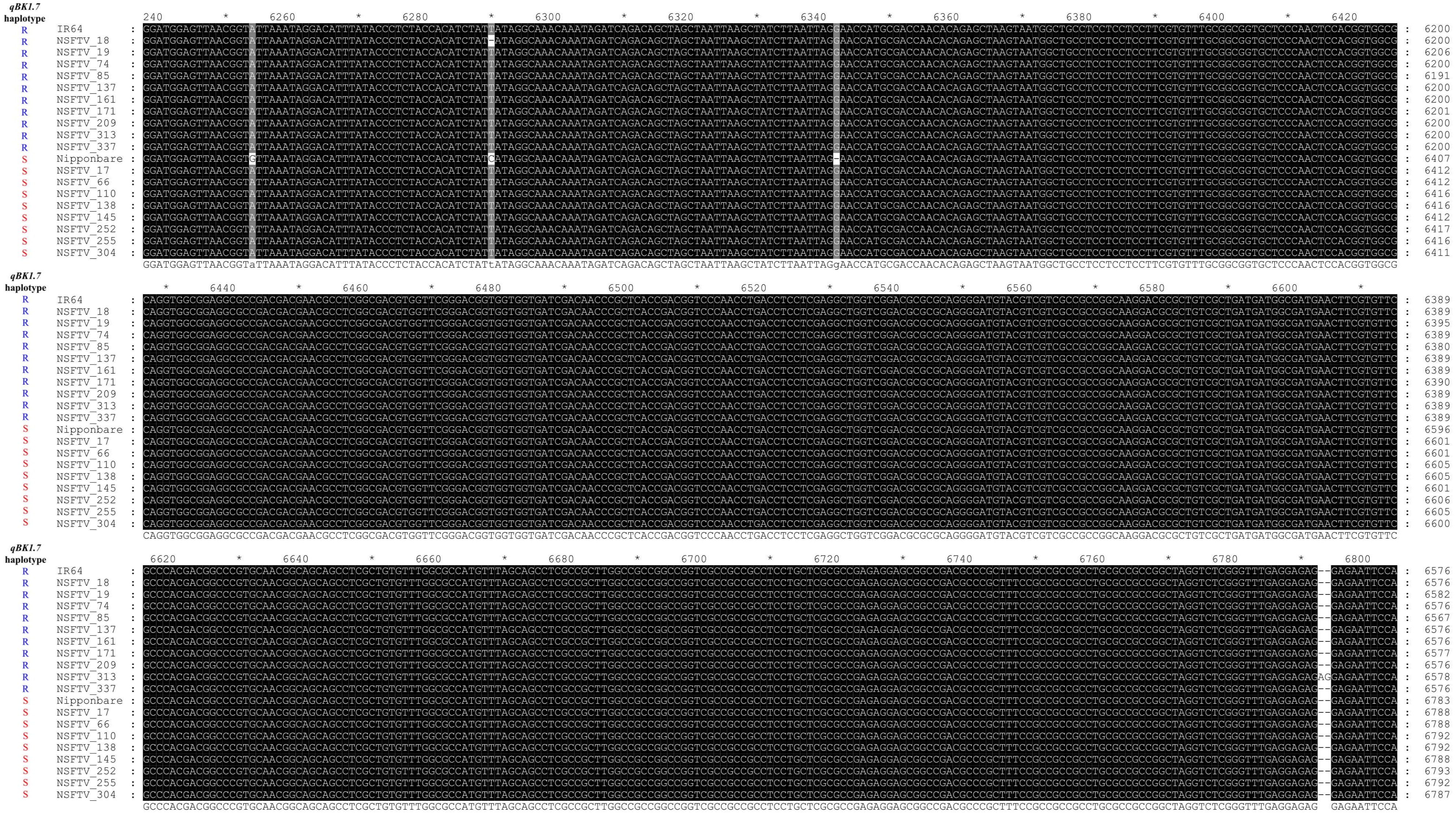
qBK1.7 haplotype
R IR64
R NSFTV_18
R NSFTV_19
R NSFTV_74
R NSFTV_85
R NSFTV_137
R NSFTV_161
R NSFTV_171
R NSFTV_209
R NSFTV_313
R NSFTV_337
S Nipponbare
S NSFTV_17
S NSFTV_66
S NSFTV_110
S NSFTV_138
S NSFTV_145
S NSFTV_252
S NSFTV_255
S NSFTV_304

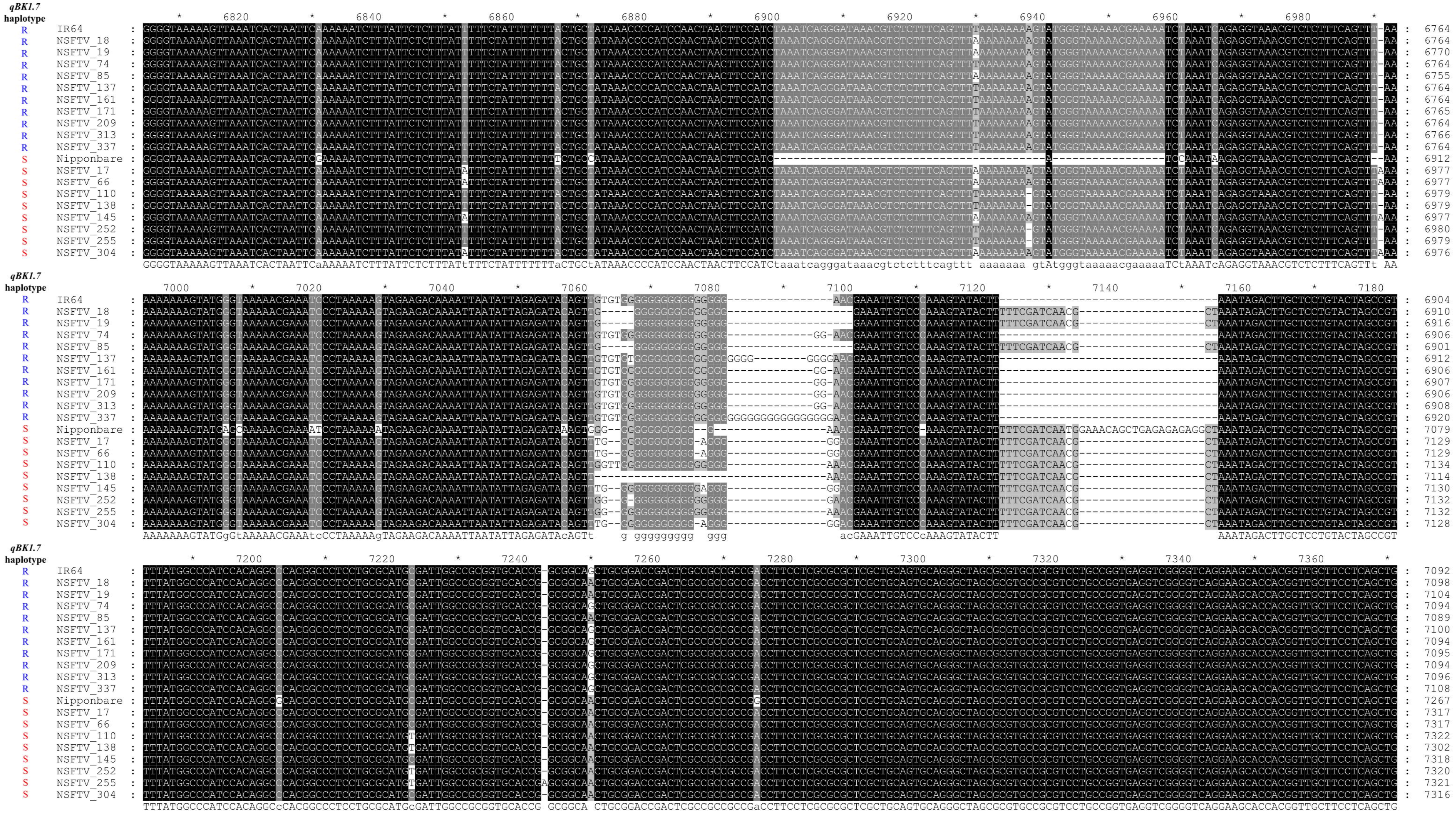

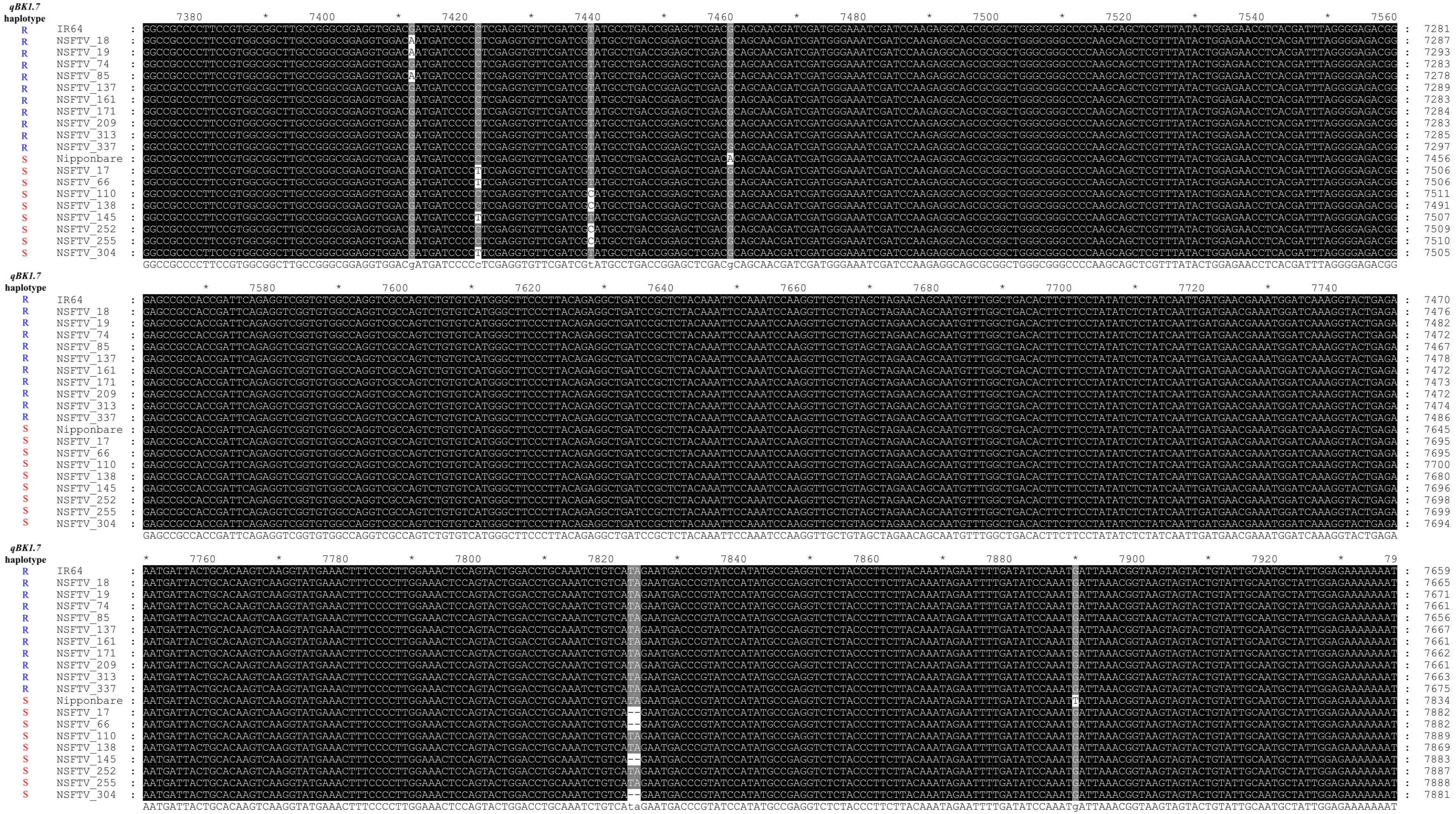
qBK1.7 haplotype
R IR64
R NSFTV_18
R NSFTV_19
R NSFTV_74
R NSFTV_85
R NSFTV_137
R NSFTV_161
R NSFTV_171
R NSFTV_209
R NSFTV_313
R NSFTV_337
S Nipponbare
S NSFTV_17
S NSFTV_66
S NSFTV_110
S NSFTV_138
S NSFTV_145
S NSFTV_252
S NSFTV_255
S NSFTV_304

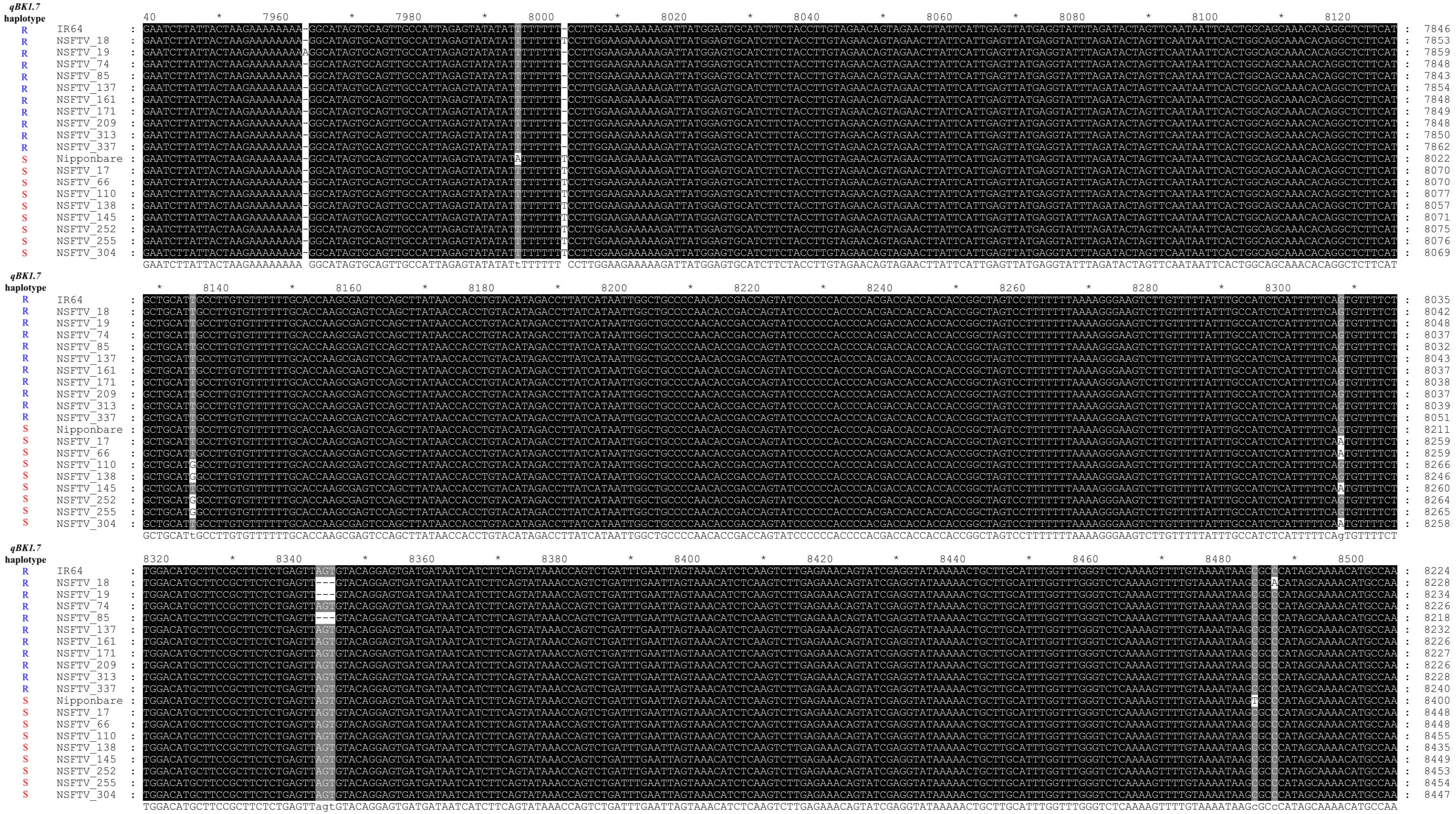
qBK1.7 haplotype
R IR64
R NSFTV_18
R NSFTV_19
R NSFTV_74
R NSFTV_85
R NSFTV_137
R NSFTV_161
R NSFTV_171
R NSFTV_209
R NSFTV_313
R NSFTV_337
S Nipponbare
S NSFTV_17
S NSFTV_66
S NSFTV_110
S NSFTV_138
S NSFTV_145
S NSFTV_252
S NSFTV_255
S NSFTV_304

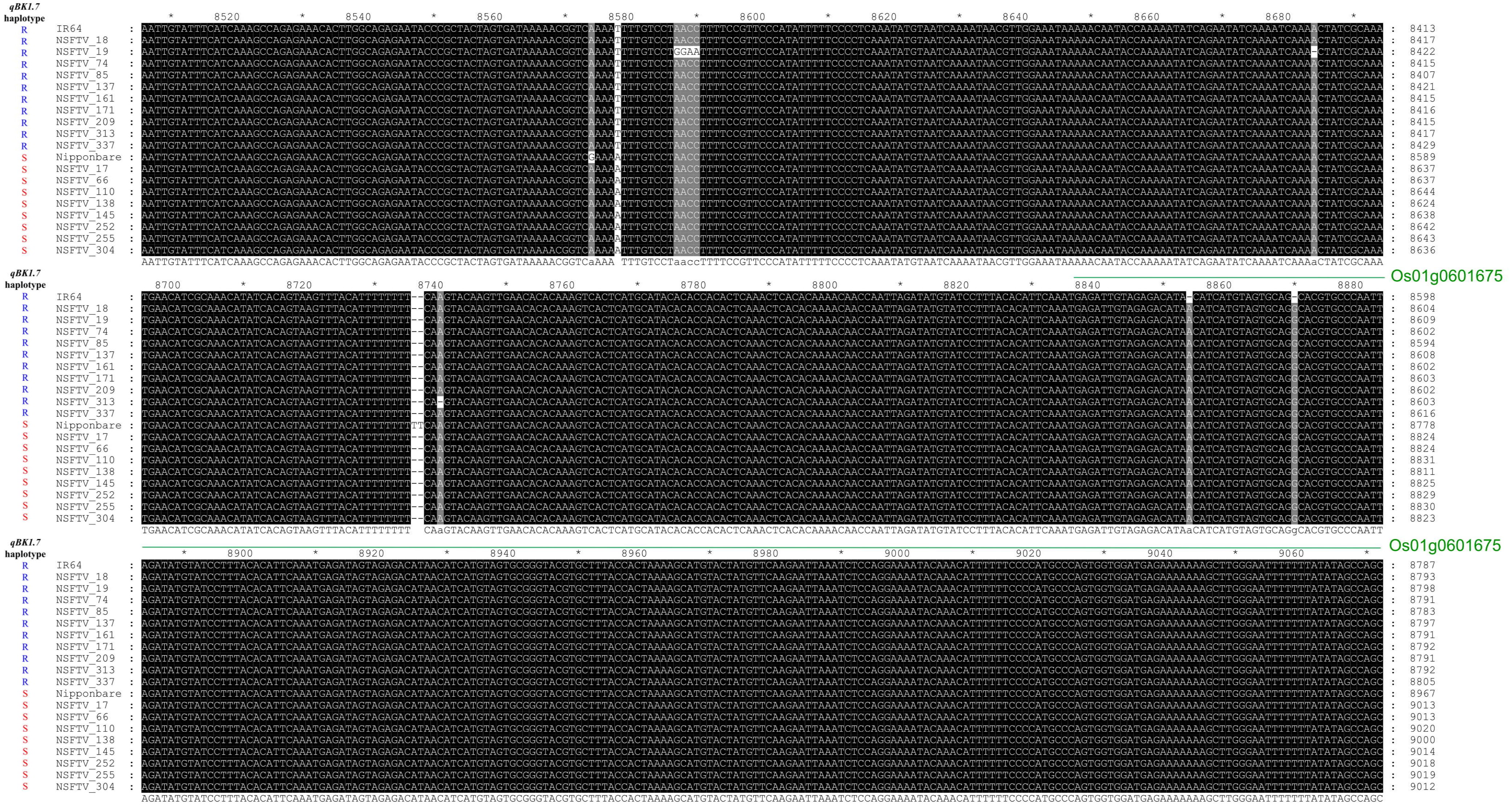
qBK1.7 haplotype
IR64
NSFTV_18
NSFTV_19
NSFTV_74
NSFTV_85
NSFTV_137
NSFTV_161
NSFTV_171
NSFTV_209
NSFTV_313
NSFTV_337
Nipponbare
NSFTV_17
NSFTV_66
NSFTV_110
NSFTV_138
NSFTV_145
NSFTV_252
NSFTV_255
NSFTV_304
Os01g0601675

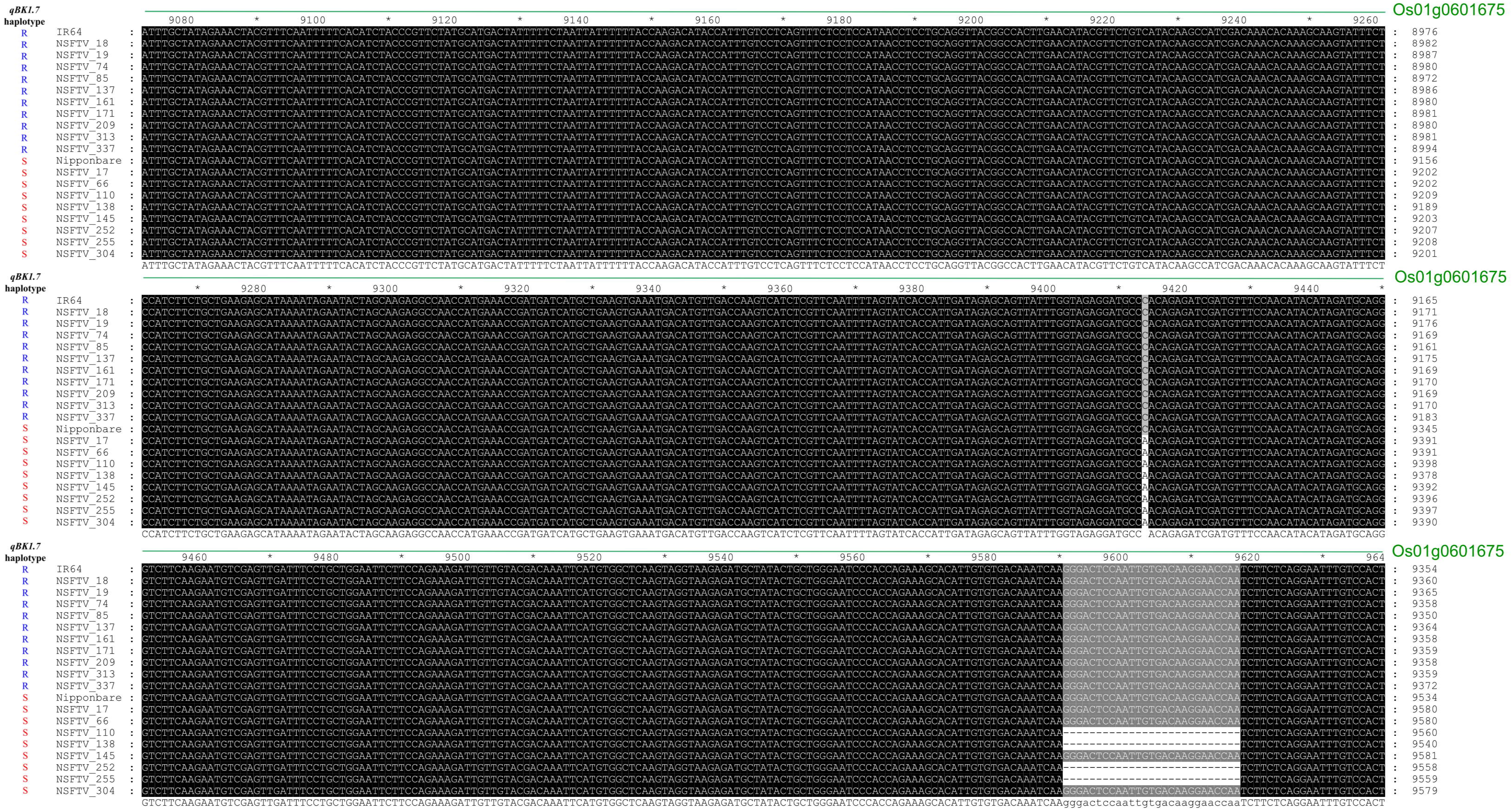
qBK1.7 haplotype
Os01g0601675
R IR64
R NSFTV_18
R NSFTV_19
R NSFTV_74
R NSFTV_85
R NSFTV_137
R NSFTV_161
R NSFTV_171
R NSFTV_209
R NSFTV_313
R NSFTV_337
S Nipponbare
S NSFTV_17
S NSFTV_66
S NSFTV_110
S NSFTV_138
S NSFTV_145
S NSFTV_252
S NSFTV_255
S NSFTV_304

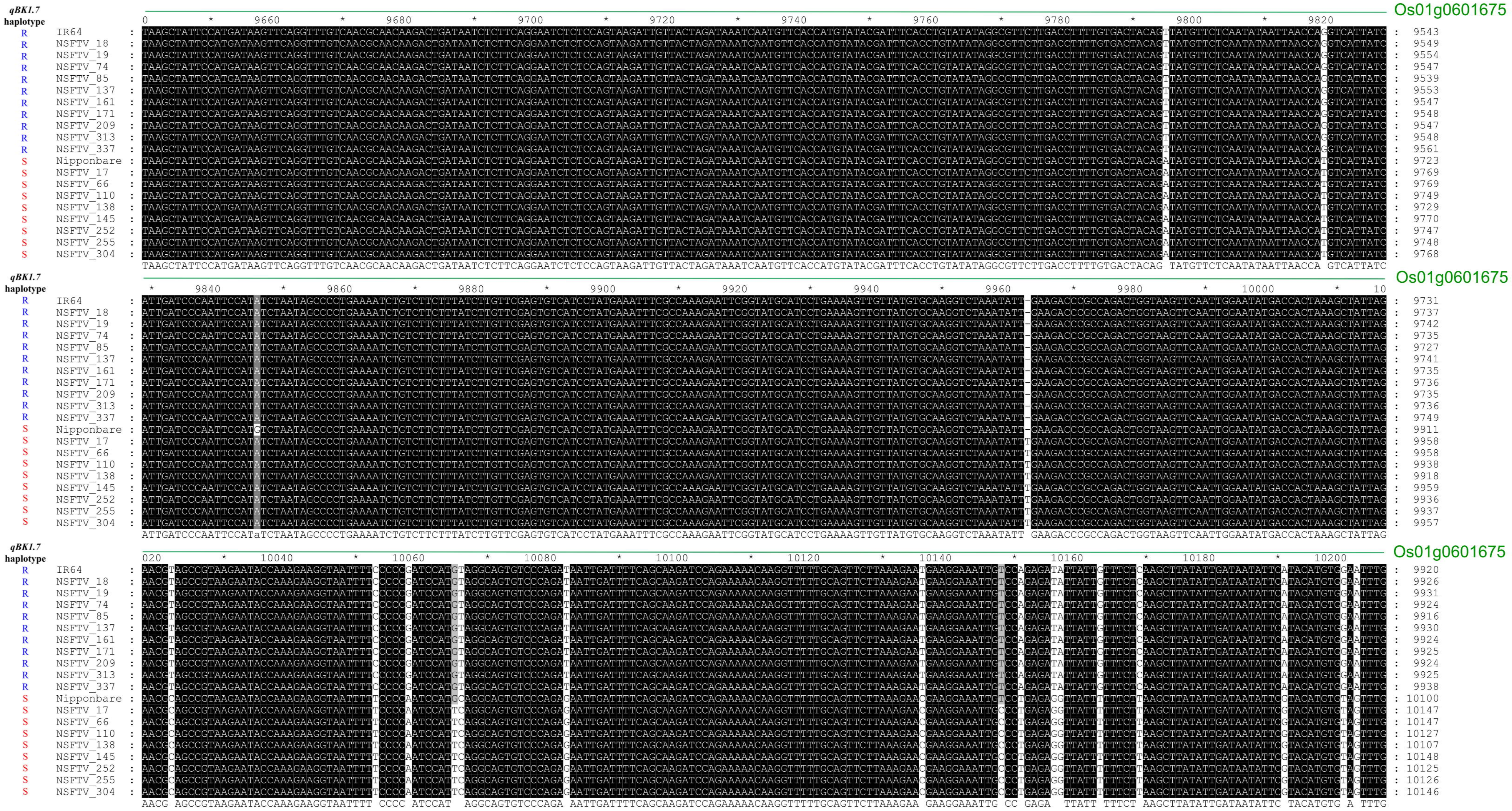

qBK1.7 haplotype
Os01g0601675
IR64
NSFTV_18
NSFTV_19
NSFTV_74
NSFTV_85
NSFTV_137
NSFTV_161
NSFTV_171
NSFTV_209
NSFTV_313
NSFTV_337
Nipponbare
NSFTV_17
NSFTV_66
NSFTV_110
NSFTV_138
NSFTV_145
NSFTV_252
NSFTV_255
NSFTV_304

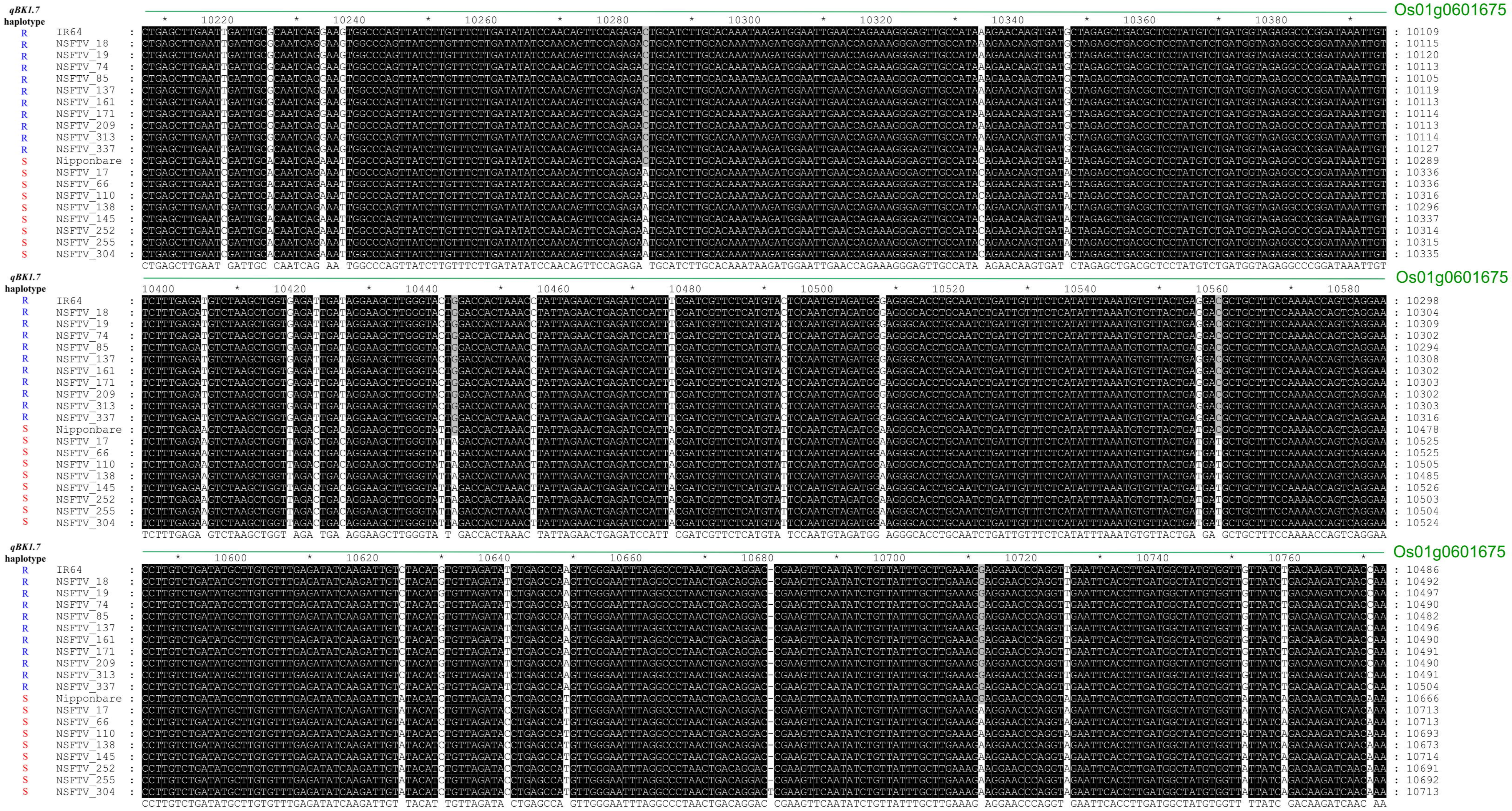
qBK1.7 haplotype
Os01g0601675
IR64
NSFTV_18
NSFTV_19
NSFTV_74
NSFTV_85
NSFTV_137
NSFTV_161
NSFTV_171
NSFTV_209
NSFTV_313
NSFTV_337
Nipponbare
NSFTV_17
NSFTV_66
NSFTV_110
NSFTV_138
NSFTV_145
NSFTV_252
NSFTV_255
NSFTV_304

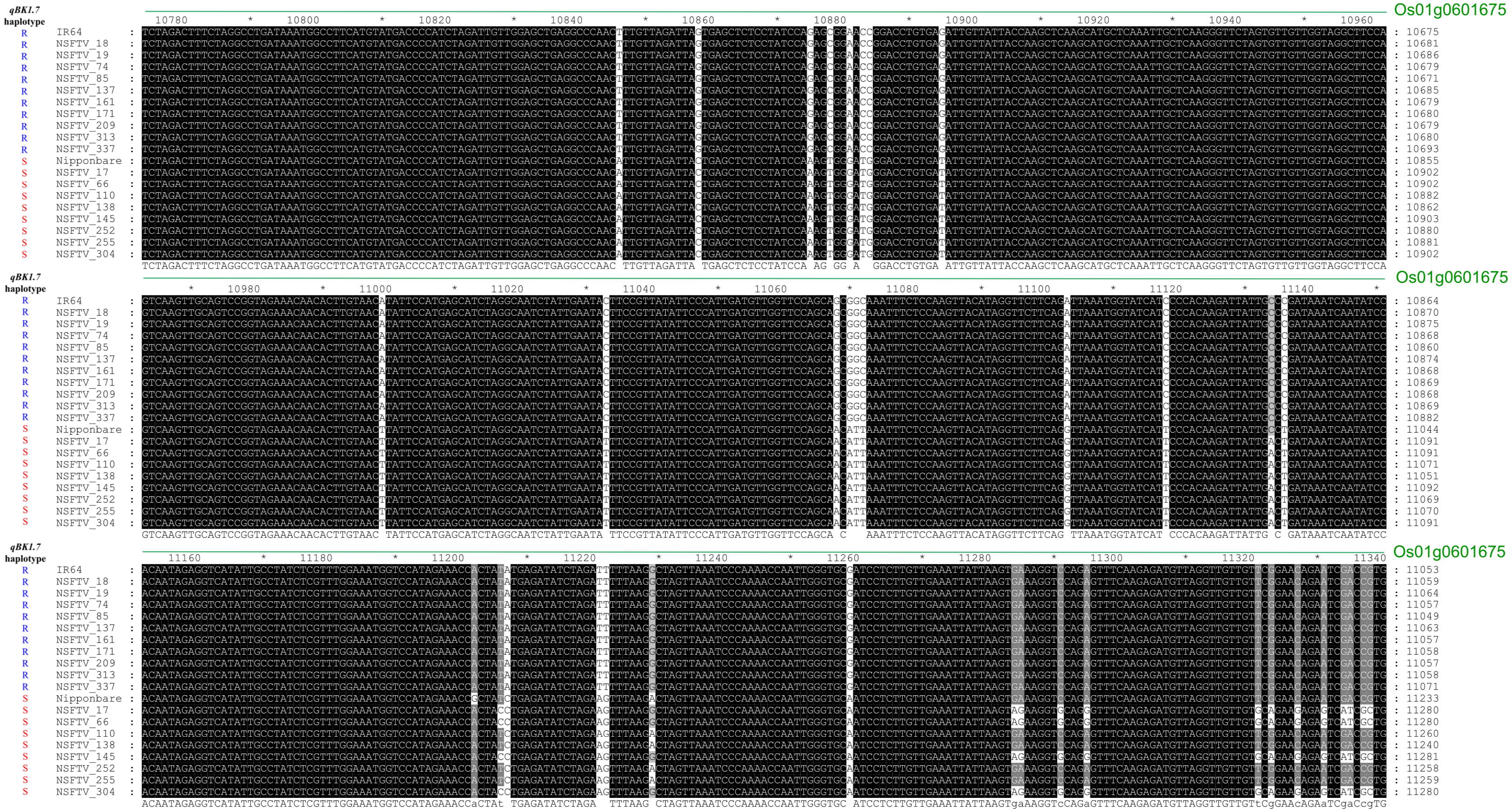
qBK1.7 haplotype
Os01g0601675
R IR64
R NSFTV_18
R NSFTV_19
R NSFTV_74
R NSFTV_85
R NSFTV_137
R NSFTV_161
R NSFTV_171
R NSFTV_209
R NSFTV_313
R NSFTV_337
S Nipponbare
S NSFTV_17
S NSFTV_66
S NSFTV_110
S NSFTV_138
S NSFTV_145
S NSFTV_252
S NSFTV_255
S NSFTV_304

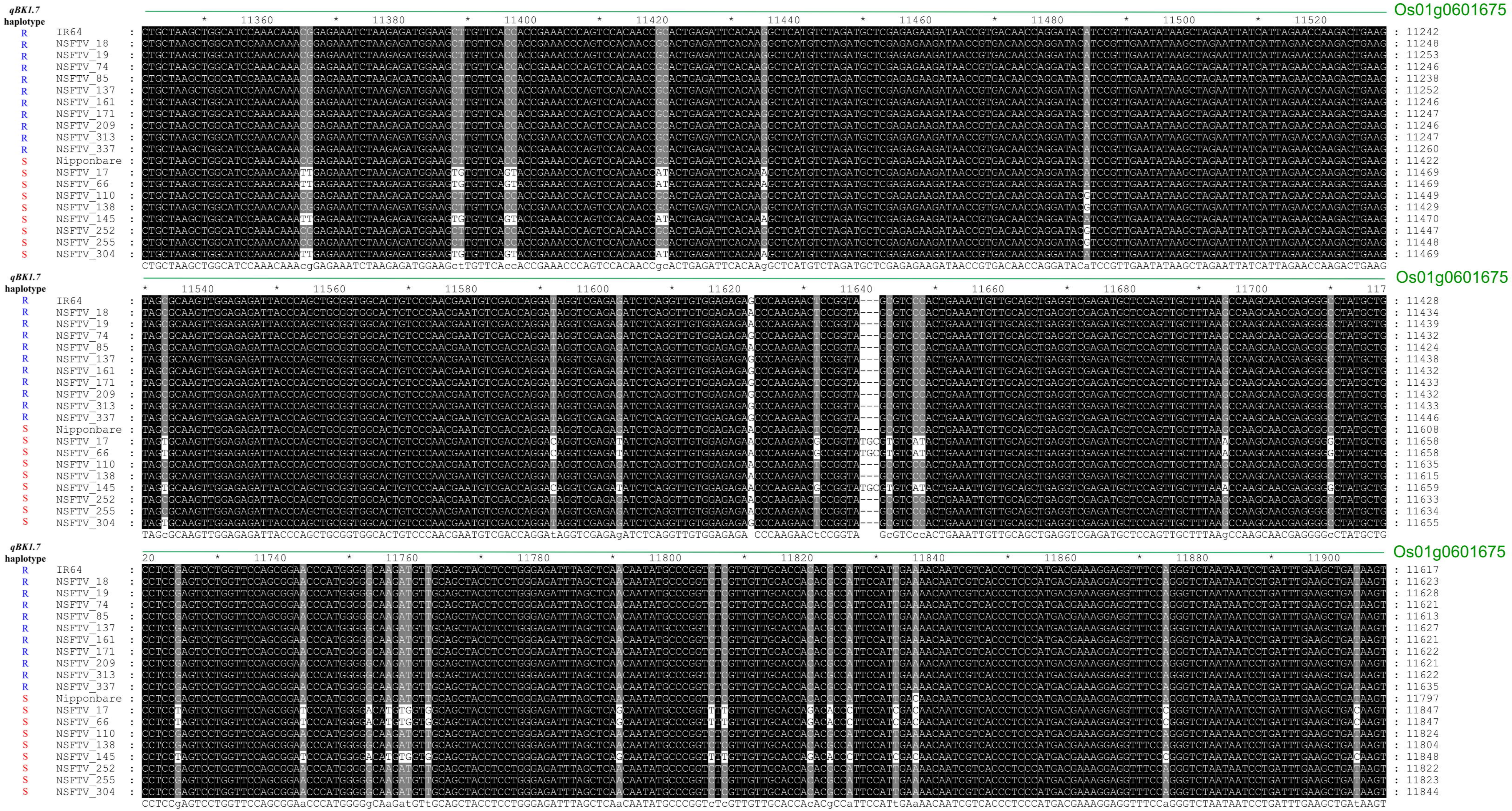
qBK1.7 haplotype
Os01g0601675
R IR64
R NSFTV_18
R NSFTV_19
R NSFTV_74
R NSFTV_85
R NSFTV_137
R NSFTV_161
R NSFTV_171
R NSFTV_209
R NSFTV_313
R NSFTV_337
S Nipponbare
S NSFTV_17
S NSFTV_66
S NSFTV_110
S NSFTV_138
S NSFTV_145
S NSFTV_252
S NSFTV_255
S NSFTV_304

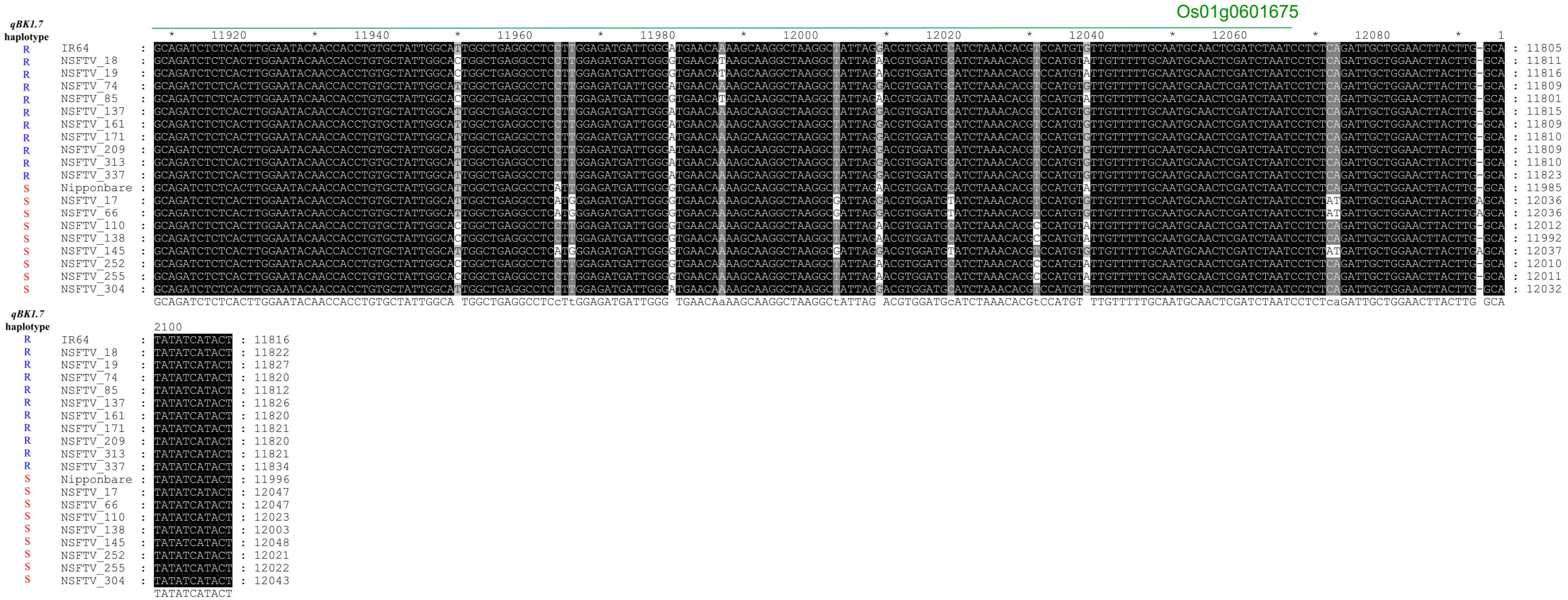
Os01g0601675
qBK1.7 haplotype
11920
11940
11960
11980
12000
12020
12040
12060
12080
R IR64 : 11805
R NSFTV_18 : 11811
R NSFTV_19 : 11816
R NSFTV_74 : 11809
R NSFTV_85 : 11801
R NSFTV_137 : 11815
R NSFTV_161 : 11809
R NSFTV_171 : 11810
R NSFTV_209 : 11809
R NSFTV_313 : 11810
R NSFTV_337 : 11823
S Nipponbare : 11985
S NSFTV_17 : 12036
S NSFTV_66 : 12036
S NSFTV_110 : 12012
S NSFTV_138 : 11992
S NSFTV_145 : 12037
S NSFTV_252 : 12010
S NSFTV_255 : 12011
S NSFTV_304 : 12032
GCAGATCTCTCACTTGGAATACAACCACCTGTGCTATTGGCA TGGCTGAGGCCTCcTtGGAGATGATTGGG TGAACAaAAGCAAGGCTAAGGCtATTAG ACGTGGATGcATCTAAACACGtCCATGT TTGTTTTTGCAATGCAACTCGATCTAATCCTCTcaGATTGCTGGAACTTACTTG GCA
qBK1.7 haplotype
2100
R IR64 : TATATCATACT : 11816
R NSFTV_18 : TATATCATACT : 11822
R NSFTV_19 : TATATCATACT : 11827
R NSFTV_74 : TATATCATACT : 11820
R NSFTV_85 : TATATCATACT : 11812
R NSFTV_137 : TATATCATACT : 11826
R NSFTV_161 : TATATCATACT : 11820
R NSFTV_171 : TATATCATACT : 11821
R NSFTV_209 : TATATCATACT : 11820
R NSFTV_313 : TATATCATACT : 11821
R NSFTV_337 : TATATCATACT : 11834
S Nipponbare : TATATCATACT : 11996
S NSFTV_17 : TATATCATACT : 12047
S NSFTV_66 : TATATCATACT : 12047
S NSFTV_110 : TATATCATACT : 12023
S NSFTV_138 : TATATCATACT : 12003
S NSFTV_145 : TATATCATACT : 12048
S NSFTV_252 : TATATCATACT : 12021
S NSFTV_255 : TATATCATACT : 12022
S NSFTV_304 : TATATCATACT : 12043
TATATCATACT
