# Supplementary material for: Genome-wide association mapping of gene loci affecting disease resistance in the rice-Fusarium fujikuroi pathosystem
Source: Rice (N Y). 2019 Nov 21;12:85. doi: 10.1186/s12284-019-0337-3 (PMC6872702; doi:10.1186/s12284-019-0337-3)
Supplement: Supplementary file 7 — Additional file 7: Fig. S2. Alignment of the amino acid sequences of Os01g0601625. [file 12284_2019_337_MOESM7_ESM.pdf]

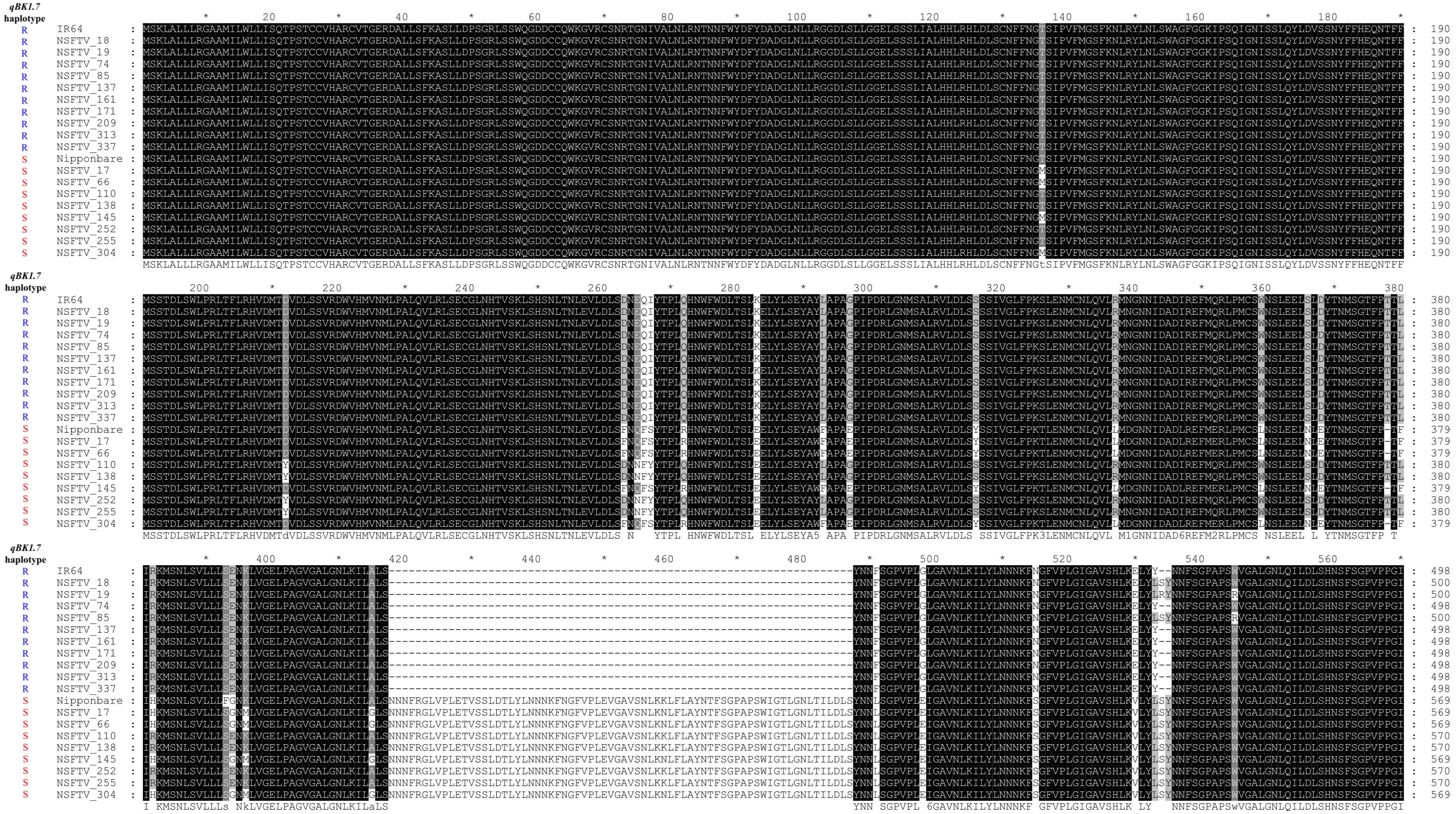

```
qBK1.7
haplotype               580         *       600         *       620         *       640         *       660         *       680         *       700         *       720         *       740         *       760
R  IR64        : GSLSNLTTLDLSYNRFQGVISKDHVEHLSRLKYLDLSYNFLKIDIHTNSSPPFKLRNASFRSCQLGPRFPLWLRWQTDIDALVLENTKLDDVIPDWFWVTFSRASFLQASGNKLHGSLPPSLEHISVGRIYLGSNLLTGQVPQLPISMTCLNLSSNFLSGPLPSLKAPLLEELLLANNNITGSIPPSMCQ : 688
R  NSFTV_18    : GSLSNLTTLDLSYNRFQGVISKDHVEHLSRLKYLDLSYNFLKIDIHTNSSPPFKLRNASFRSCQLGPRFPLWLRWQTDIDALVLENTKLDDVIPDWFWVTFSRASFLQASGNKLHGSLPPSLEHISVGRIYLGSNLLTGQVPQLPISMTCLNLSSNFLSGPLPSLKAPLLEELLLANNNITGSIPPSMCQ : 690
R  NSFTV_19    : GSLSNLTTLDLSYNRFQGVISKDHVEHLSRLKYLDLSYNFLKIDIHTNSSPPFKLRNASFRSCQLGPRFPLWLRWQTDIDALVLENTKLDDVIPDWFWVTFSRASFLQASGNKLHGSLPPSLEHISVGRIYLGSNLLTGQVPQLPISMTCLNLSSNFLSGPLPSLKAPLLEELLLANNNITGSIPPSMCQ : 690
R  NSFTV_74    : GSLSNLTTLDLSYNRFQGVISKDHVEHLSRLKYLDLSYNFLKIDIHTNSSPPFKLRNASFRSCQLGPRFPLWLRWQTDIDALVLENTKLDDVIPDWFWVTFSRASFLQASGNKLHGSLPPSLEHISVGRIYLGSNLLTGQVPQLPISMTCLNLSSNFLSGPLPSLKAPLLEELLLANNNITGSIPPSMCQ : 688
R  NSFTV_85    : GSLSNLTTLDLSYNRFQGVISKDHVEHLSRLKYLDLSYNFLKIDIHTNSSPPFKLRNASFRSCQLGPRFPLWLRWQTDIDALVLENTKLDDVIPDWFWVTFSRASFLQASGNKLHGSLPPSLEHISVGRIYLGSNLLTGQVPQLPISMTCLNLSSNFLSGPLPSLKAPLLEELLLANNNITGSIPPSMCQ : 690
R  NSFTV_137   : GSLSNLTTLDLSYNRFQGVISKDHVEHLSRLKYLDLSYNFLKIDIHTNSSPPFKLRNASFRSCQLGPRFPLWLRWQTDIDALVLENTKLDDVIPDWFWVTFSRASFLQASGNKLHGSLPPSLEHISVGRIYLGSNLLTGQVPQLPISMTCLNLSSNFLSGPLPSLKAPLLEELLLANNNITGSIPPSMCQ : 688
R  NSFTV_161   : GSLSNLTTLDLSYNRFQGVISKDHVEHLSRLKYLDLSYNFLKIDIHTNSSPPFKLRNASFRSCQLGPRFPLWLRWQTDIDALVLENTKLDDVIPDWFWVTFSRASFLQASGNKLHGSLPPSLEHISVGRIYLGSNLLTGQVPQLPISMTCLNLSSNFLSGPLPSLKAPLLEELLLANNNITGSIPPSMCQ : 688
R  NSFTV_171   : GSLSNLTTLDLSYNRFQGVISKDHVEHLSRLKYLDLSYNFLKIDIHTNSSPPFKLRNASFRSCQLGPRFPLWLRWQTDIDALVLENTKLDDVIPDWFWVTFSRASFLQASGNKLHGSLPPSLEHISVGRIYLGSNLLTGQVPQLPISMTCLNLSSNFLSGPLPSLKAPLLEELLLANNNITGSIPPSMCQ : 688
R  NSFTV_209   : GSLSNLTTLDLSYNRFQGVISKDHVEHLSRLKYLDLSYNFLKIDIHTNSSPPFKLRNASFRSCQLGPRFPLWLRWQTDIDALVLENTKLDDVIPDWFWVTFSRASFLQASGNKLHGSLPPSLEHISVGRIYLGSNLLTGQVPQLPISMTCLNLSSNFLSGPLPSLKAPLLEELLLANNNITGSIPPSMCQ : 688
R  NSFTV_313   : GSLSNLTTLDLSYNRFQGVISKDHVEHLSRLKYLDLSYNFLKIDIHTNSSPPFKLRNASFRSCQLGPRFPLWLRWQTDIDALVLENTKLDDVIPDWFWVTFSRASFLQASGNKLHGSLPPSLEHISVGRIYLGSNLLTGQVPQLPISMTCLNLSSNFLSGPLPSLKAPLLEELLLANNNITGSIPPSMCQ : 688
R  NSFTV_337   : GSLSNLTTLDLSYNRFQGVISKDHVEHLSRLKYLDLSYNFLKIDIHTNSSPPFKLRNASFRSCQLGPRFPLWLRWQTDIDALVLENTKLDDVIPDWFWVTFSRASFLQASGNKLHGSLPPSLEHISVGRIYLGSNLLTGQVPQLPISMTCLNLSSNFLSGPLPSLKAPLLEELLLANNNITGSIPPSMCQ : 688
S  Nipponbare  : GSLSNLTTLDLSYNRFQGVISKDHVEHLSRLKYLDLSDNFLKIDIHTNSSPPFKLRNAAFRSCQLGPRFPLWLRWQTDIDVLVLENTKLDDVIPDWFWVTFSRASFLQASGNKLHGSLPPSLEHISVGRIYLGSNLLTGQVPQLPISMTRLNLSSNFLSGPLPSLKAPLLEELLLANNNITGSIPPSMCQ : 759
S  NSFTV_17    : GSLSNLTTLDLSYNRFQGVISKDHVEHLSRLKYLDLSDNFLKIDIHTNFSPPFKLRNAAFRSCQLGPRFPLWLRWQTDIDVLVLENTKLDDVIPDWFWVTFSRASFLQASGNKLHGSLPPSLEHISVGRIYLGSNLLTGQVPQLPISMTRLNLSSNFLSGPLPSLKAPLLEELLLANNNITGSIPPSMCQ : 759
S  NSFTV_66    : GSLSNLTTLDLSYNRFQGVISKDHVEHLSRLKYLDLSDNFLKIDIHTNFSPPFKLRNAAFRSCQLGPRFPLWLRWQTDIDVLVLENTKLDDVIPDWFWVTFSRASFLQASGNKLHGSLPPSLEHISVGRIYLGSNLLTGQVPQLPISMTRLNLSSNFLSGPLPSLKAPLLEELLLANNNITGSIPPSMCQ : 759
S  NSFTV_110   : GSLSNLTTLDLSYNRFQGVISKDHVEHLSRLKYLDLSDNFLKIDIHTNFSPPFKLRNAAFRSCQLGPRFPLWLRWQTDIDVLVLENTKLDDVIPDWFWVTFSRASFLQASGNKLHGSLPPSLEHISVGRIYLGSNLLTGQVPQLPISMTRLNLSSNFLSGPLPSLKAPLLEELLLANNNITGSIPPSMCQ : 760
S  NSFTV_138   : GSLSNLTTLDLSYNRFQGVISKDHVEHLSRLKYLDLSDNFLKIDIHTNFSPPFKLRNAAFRSCQLGPRFPLWLRWQTDIDVLVLENTKLDDVIPDWFWVTFSRASFLQASGNKLHGSLPPSLEHISVGRIYLGSNLLTGQVPQLPISMTRLNLSSNFLSGPLPSLKAPLLEELLLANNNITGSIPPSMCQ : 760
S  NSFTV_145   : GSLSNLTTLDLSYNRFQGVISKDHVEHLSRLKYLDLSDNFLKIDIHTNFSPPFKLRNAAFRSCQLGPRFPLWLRWQTDIDVLVLENTKLDDVIPDWFWVTFSRASFLQASGNKLHGSLPPSLEHISVGRIYLGSNLLTGQVPQLPISMTRLNLSSNFLSGPLPSLKAPLLEELLLANNNITGSIPPSMCQ : 759
S  NSFTV_252   : GSLSNLTTLDLSYNRFQGVISKDHVEHLSRLKYLDLSDNFLKIDIHTNFSPPFKLRNAAFRSCQLGPRFPLWLRWQTDIDVLVLENTKLDDVIPDWFWVTFSRASFLQASGNKLHGSLPPSLEHISVGRIYLGSNLLTGQVPQLPISMTRLNLSSNFLSGPLPSLKAPLLEELLLANNNITGSIPPSMCQ : 760
S  NSFTV_255   : GSLSNLTTLDLSYNRFQGVISKDHVEHLSRLKYLDLSDNFLKIDIHTNFSPPFKLRNAAFRSCQLGPRFPLWLRWQTDIDVLVLENTKLDDVIPDWFWVTFSRASFLQASGNKLHGSLPPSLEHISVGRIYLGSNLLTGQVPQLPISMTRLNLSSNFLSGPLPSLKAPLLEELLLANNNITGSIPPSMCQ : 760
S  NSFTV_304   : GSLSNLTTLDLSYNRFQGVISKDHVEHLSRLKYLDLSDNFLKIDIHTNFSPPFKLRNAAFRSCQLGPRFPLWLRWQTDIDVLVLENTKLDDVIPDWFWVTFSRASFLQASGNKLHGSLPPSLEHISVGRIYLGSNLLTGQVPQLPISMTRLNLSSNFLSGPLPSLKAPLLEELLLANNNITGSIPPSMCQ : 759
                 GSLSNLTTLDLSYNRFQGVISKDHVEHLSRLKYLDLS NFLKIDIHTN SPPFKLRNA FRSCQLGPRFPLWLRWQTDID LVLENTKLDDVIPDWFWVTFSRASFLQASGNKLHGSLPPSLEHISVGRIYLGSNLLTGQVPQLPISMT LNLSSNFLSGPLPSLKAPLLEELLLANNNITGSIPPSMCQ
```

```
qBK1.7
haplotype                   *       780         *       800         *       820         *       840         *       860         *       880         *       900         *       920         *       940         *
R  IR64        : LTGLNRLDLSGNKITGDLEQMQCWKQSDMPNTNSADKFGSSMLSLALNHNELSGIFPQFLQNASQLLFLDLSHNRFFGSLPKWLPERMPNLQILRLRSNIFHGHIPKNIIYLGKLHFLDIAHNNISGSIPDSLANFKAMTVIAQNSEDYIFEESIPVITKDQQRDYTFEIYNQVVNLDFSCNKLTAHIPE : 878
R  NSFTV_18    : LTGLNRLDLSGNKITGDLEQMQCWKQSDMPNTNSADKFGSSMLSLALNHNELSGIFPQFLQNASQLLFLDLSHNRFFGSLPKWLPERMPNLQILRLRSNIFHGHIPKNIIYLGKLHFLDIAHNNISGSIPDSLANFKAMTVIAQNSEDYIFEESIPVITKDQQRDYTFEIYNQVVNLDFSCNKLTAHIPE : 880
R  NSFTV_19    : LTGLNRLDLSGNKITGDLEQMQCWKQSDMPNTNSADKFGSSMLSLALNHNELSGIFPQFLQNASQLLFLDLSHNRFFGSLPKWLPERMPNLQILRLRSNIFHGHIPKNIIYLGKLHFLDIAHNNISGSIPDSLANFKAMTVIAQNSEDYIFEESIPVITKDQQRDYTFEIYNQVVNLDFSCNKLTAHIPE : 880
R  NSFTV_74    : LTGLNRLDLSGNKITGDLEQMQCWKQSDMPNTNSADKFGSSMLSLALNHNELSGIFPQFLQNASQLLFLDLSHNRFFGSLPKWLPERMPNLQILRLRSNIFHGHIPKNIIYLGKLHFLDIAHNNISGSIPDSLANFKAMTVIAQNSEDYIFEESIPVITKDQQRDYTFEIYNQVVNLDFSCNKLTAHIPE : 878
R  NSFTV_85    : LTGLNRLDLSGNKITGDLEQMQCWKQSDMPNTNSADKFGSSMLSLALNHNELSGIFPQFLQNASQLLFLDLSHNRFFGSLPKWLPERMPNLQILRLRSNIFHGHIPKNIIYLGKLHFLDIAHNNISGSIPDSLANFKAMTVIAQNSEDYIFEESIPVITKDQQRDYTFEIYNQVVNLDFSCNKLTAHIPE : 880
R  NSFTV_137   : LTGLNRLDLSGNKITGDLEQMQCWKQSDMPNTNSADKFGSSMLSLALNHNELSGIFPQFLQNASQLLFLDLSHNRFFGSLPKWLPERMPNLQILRLRSNIFHGHIPKNIIYLGKLHFLDIAHNNISGSIPDSLANFKAMTVIAQNSEDYIFEESIPVITKDQQRDYTFEIYNQVVNLDFSCNKLTAHIPE : 878
R  NSFTV_161   : LTGLNRLDLSGNKITGDLEQMQCWKQSDMPNTNSADKFGSSMLSLALNHNELSGIFPQFLQNASQLLFLDLSHNRFFGSLPKWLPERMPNLQILRLRSNIFHGHIPKNIIYLGKLHFLDIAHNNISGSIPDSLANFKAMTVIAQNSEDYIFEESIPVITKDQQRDYTFEIYNQVVNLDFSCNKLTAHIPE : 878
R  NSFTV_171   : LTGLNRLDLSGNKITGDLEQMQCWKQSDMPNTNSADKFGSSMLSLALNHNELSGIFPQFLQNASQLLFLDLSHNRFFGSLPKWLPERMPNLQILRLRSNIFHGHIPKNIIYLGKLHFLDIAHNNISGSIPDSLANFKAMTVIAQNSEDYIFEESIPVITKDQQRDYTFEIYNQVVNLDFSCNKLTAHIPE : 878
R  NSFTV_209   : LTGLNRLDLSGNKITGDLEQMQCWKQSDMPNTNSADKFGSSMLSLALNHNELSGIFPQFLQNASQLLFLDLSHNRFFGSLPKWLPERMPNLQILRLRSNIFHGHIPKNIIYLGKLHFLDIAHNNISGSIPDSLANFKAMTVIAQNSEDYIFEESIPVITKDQQRDYTFEIYNQVVNLDFSCNKLTAHIPE : 878
R  NSFTV_313   : LTGLNRLDLSGNKITGDLEQMQCWKQSDMPNTNSADKFGSSMLSLALNHNELSGIFPQFLQNASQLLFLDLSHNRFFGSLPKWLPERMPNLQILRLRSNIFHGHIPKNIIYLGKLHFLDIAHNNISGSIPDSLANFKAMTVIAQNSEDYIFEESIPVITKDQQRDYTFEIYNQVVNLDFSCNKLTAHIPE : 878
R  NSFTV_337   : LTGLNRLDLSGNKITGDLEQMQCWKQSDMPNTNSADKFGSSMLSLALNHNELSGIFPQFLQNASQLLFLDLSHNRFFGSLPKWLPERMPNLQILRLRSNIFHGHIPKNIIYLGKLHFLDIAHNNISGSIPDSLANFKAMTVIAQNSEDYIFEESIPVITKDQQRDYTFEIYNQVVNLDFSCNKLTAHIPE : 878
S  Nipponbare  : LTGLKRLDLSGNKITGDLEQMQCWKQSDMTNTNSADKFGSSMLSLALNHNELSGIFPQFLQNASQLLFLDLSHNRFFGSLPKWLPERMPNLQILRLRSNIFHGHIPKNIIYLGKLHFLDIAHNNISGSIPDSLANFKAMTVIAQNSEDYIFEESIPVITKDQQRDYTFEIYNQVVNLDFSCNKLTGHIPE : 949
S  NSFTV_17    : LTGLKRLDLSGNKITGDLEQMQCWKQSDMTNTNSADKFGSSMLSLALNHNELSGIFPQFLQNASQLLFLDLSHNRFFGSLPKWLPERMPNLQILRLRSNIFHGHIPKNIIYLGKLHFLDIAHNNISGSIPDSLANFKAMTVIAQNSEDYIFEESIPVITKDQQRDYTFEIYNQVVNLDFSCNKLTGHIPE : 949
S  NSFTV_66    : LTGLKRLDLSGNKITGDLEQMQCWKQSDMTNTNSADKFGSSMLSLALNHNELSGIFPQFLQNASQLLFLDLSHNRFFGSLPKWLPERMPNLQILRLRSNIFHGHIPKNIIYLGKLHFLDIAHNNISGSIPDSLANFKAMTVIAQNSEDYIFEESIPVITKDQQRDYTFEIYNQVVNLDFSCNKLTGHIPE : 949
S  NSFTV_110   : LTGLKRLDLSGNKITGDLEQMQCWKQSDMTNTNSADKFGSSMLSLALNHNELSGIFPQFLQNASQLLFLDLSHNRFFGSLPKWLPERMPNLQILRLRSNIFHGHIPKNIIYLGKLHFLDIAHNNISGSIPDSLANFKAMTVIAQNSEDYIFEESIPVITKDQQRDYTFEIYNQVVNLDFSCNKLTGHIPE : 950
S  NSFTV_138   : LTGLKRLDLSGNKITGDLEQMQCWKQSDMTNTNSADKFGSSMLSLALNHNELSGIFPQFLQNASQLLFLDLSHNRFFGSLPKWLPERMPNLQILRLRSNIFHGHIPKNIIYLGKLHFLDIAHNNISGSIPDSLANFKAMTVIAQNSEDYIFEESIPVITKDQQRDYTFEIYNQVVNLDFSCNKLTGHIPE : 950
S  NSFTV_145   : LTGLKRLDLSGNKITGDLEQMQCWKQSDMTNTNSADKFGSSMLSLALNHNELSGIFPQFLQNASQLLFLDLSHNRFFGSLPKWLPERMPNLQILRLRSNIFHGHIPKNIIYLGKLHFLDIAHNNISGSIPDSLANFKAMTVIAQNSEDYIFEESIPVITKDQQRDYTFEIYNQVVNLDFSCNKLTGHIPE : 949
S  NSFTV_252   : LTGLKRLDLSGNKITGDLEQMQCWKQSDMTNTNSADKFGSSMLSLALNHNELSGIFPQFLQNASQLLFLDLSHNRFFGSLPKWLPERMPNLQILRLRSNIFHGHIPKNIIYLGKLHFLDIAHNNISGSIPDSLANFKAMTVIAQNSEDYIFEESIPVITKDQQRDYTFEIYNQVVNLDFSCNKLTGHIPE : 950
S  NSFTV_255   : LTGLKRLDLSGNKITGDLEQMQCWKQSDMTNTNSADKFGSSMLSLALNHNELSGIFPQFLQNASQLLFLDLSHNRFFGSLPKWLPERMPNLQILRLRSNIFHGHIPKNIIYLGKLHFLDIAHNNISGSIPDSLANFKAMTVIAQNSEDYIFEESIPVITKDQQRDYTFEIYNQVVNLDFSCNKLTGHIPE : 950
S  NSFTV_304   : LTGLKRLDLSGNKITGDLEQMQCWKQSDMTNTNSADKFGSSMLSLALNHNELSGIFPQFLQNASQLLFLDLSHNRFFGSLPKWLPERMPNLQILRLRSNIFHGHIPKNIIYLGKLHFLDIAHNNISGSIPDSLANFKAMTVIAQNSEDYIFEESIPVITKDQQRDYTFEIYNQVVNLDFSCNKLTGHIPE : 949
                 LTGL RLDLSGNKITGDLEQMQCWKQSDM NTNSADKFGSSMLSLALNHNELSGIFPQFLQNASQLLFLDLSHNRFFGSLPKWLPERMPNLQILRLRSNIFHGHIPKNIIYLGKLHFLDIAHNNISGSIPDSLANFKAMTVIAQNSEDYIFEESIPVITKDQQRDYTFEIYNQVVNLDFSCNKLT HIPE
```

```
qBK1.7
haplotype               960         *       980         *      1000         *      1020         *      1040         *      1060         *      1080         *      1100         *      1120
R  IR64        : EIHLLIGLTNLNLSSNQFSGTIHDQIGDLKQLESLDLSYNELSGEIPPSLSALTSLSHLNLSYNNLSGTIPSGSQLQALDDQIYIYVGNPGLCGPPLLKNCSTNGTQQSFYEDRSHMGSLYLGMSIGFVIGLWTVFCTMMMKRTWMMAYFRIIDNLYDKAYVQVAISWSRLMRKNQDAA : 1057
R  NSFTV_18    : EIHLLIGLTNLNLSSNQFSGTIHDQIGDLKQLESLDLSYNELSGEIPPSLSALTSLSHLNLSYNNLSGTIPSGSQLQALDDQIYIYVGNPGLCGPPLLKNCSTNGTQQSFYEDRSHMGSLYLGMSIGFVIGLWTVFCTMMMKRTWMMAYFRIIDNLYDKAYVQVAISWSRLMRKNQDAA : 1059
R  NSFTV_19    : EIHLLIGLTNLNLSSNQFSGTIHDQIGDLKQLESLDLSYNELSGEIPPSLSALTSLSHLNLSYNNLSGTIPSGSQLQALDDQIYIYVGNPGLCGPPLLKNCSTNGTQQSFYEDRSHMGSLYLGMSIGFVIGLWTVFCTMMMKRTWMMAYFRIIDNLYDKAYVQVAISWSRLMRKNQDAA : 1059
R  NSFTV_74    : EIHLLIGLTNLNLSSNQFSGTIHDQIGDLKQLESLDLSYNELSGEIPPSLSALTSLSHLNLSYNNLSGTIPSGSQLQALDDQIYIYVGNPGLCGPPLLKNCSTNGTQQSFYEDRSHMGSLYLGMSIGFVIGLWTVFCTMMMKRTWMMAYFRIIDNLYDKAYVQVAISWSRLMRKNQDAA : 1057
R  NSFTV_85    : EIHLLIGLTNLNLSSNQFSGTIHDQIGDLKQLESLDLSYNELSGEIPPSLSALTSLSHLNLSYNNLSGTIPSGSQLQALDDQIYIYVGNPGLCGPPLLKNCSTNGTQQSFYEDRSHMGSLYLGMSIGFVIGLWTVFCTMMMKRTWMMAYFRIIDNLYDKAYVQVAISWSRLMRKNQDAA : 1059
R  NSFTV_137   : EIHLLIGLTNLNLSSNQFSGTIHDQIGDLKQLESLDLSYNELSGEIPPSLSALTSLSHLNLSYNNLSGTIPSGSQLQALDDQIYIYVGNPGLCGPPLLKNCSTNGTQQSFYEDRSHMGSLYLGMSIGFVIGLWTVFCTMMMKRTWMMAYFRIIDNLYDKAYVQVAISWSRLMRKNQDAA : 1057
R  NSFTV_161   : EIHLLIGLTNLNLSSNQFSGTIHDQIGDLKQLESLDLSYNELSGEIPPSLSALTSLSHLNLSYNNLSGTIPSGSQLQALDDQIYIYVGNPGLCGPPLLKNCSTNGTQQSFYEDRSHMGSLYLGMSIGFVIGLWTVFCTMMMKRTWMMAYFRIIDNLYDKAYVQVAISWSRLMRKNQDAA : 1057
R  NSFTV_171   : EIHLLIGLTNLNLSSNQFSGTIHDQIGDLKQLESLDLSYNELSGEIPPSLSALTSLSHLNLSYNNLSGTIPSGSQLQALDDQIYIYVGNPGLCGPPLLKNCSTNGTQQSFYEDRSHMGSLYLGMSIGFVIGLWTVFCTMMMKRTWMMAYFRIIDNLYDKAYVQVAISWSRLMRKNQDAA : 1057
R  NSFTV_209   : EIHLLIGLTNLNLSSNQFSGTIHDQIGDLKQLESLDLSYNELSGEIPPSLSALTSLSHLNLSYNNLSGTIPSGSQLQALDDQIYIYVGNPGLCGPPLLKNCSTNGTQQSFYEDRSHMGSLYLGMSIGFVIGLWTVFCTMMMKRTWMMAYFRIIDNLYDKAYVQVAISWSRLMRKNQDAA : 1057
R  NSFTV_313   : EIHLLIGLTNLNLSSNQFSGTIHDQIGDLKQLESLDLSYNELSGEIPPSLSALTSLSHLNLSYNNLSGTIPSGSQLQALDDQIYIYVGNPGLCGPPLLKNCSTNGTQQSFYEDRSHMGSLYLGMSIGFVIGLWTVFCTMMMKRTWMMAYFRIIDNLYDKAYVQVAISWSRLMRKNQDAA : 1057
R  NSFTV_337   : EIHLLIGLTNLNLSSNQFSGTIHDQIGDLKQLESLDLSYNELSGEIPPSLSALTSLSHLNLSYNNLSGTIPSGSQLQALDDQIYIYVGNPGLCGPPLLKNCSTNGTQQSFYEDRSHMGSLYLGMSIGFVIGLWTVFCTMMMKRTWMMAYFRIIDNLYDKAYVQVAISWSRLMRKNQDAA : 1057
S  Nipponbare  : EIHLLIGLTNLNLSSNQFSGTIHDQIGDLKQLESLDLSYNELSGEIPPSLSALTSLSHLNLSYNNLSGTIPSGSQLQALDDQIYIYVGNPGLCGPPLLKNCSTNGTQQSFYEDRSHMRSLYLGMSIGFVIGLWTVFCTMMMKRTWMMAYFRIIDNLYDKAYVQVAISWSRLMRKNQDAA : 1128
S  NSFTV_17    : EIHLLIGLTNLNLSSNQFSGTIHDQIGDLKQLESLDLSYNELSGEIPPSLSALTSLSHLNLSYNNLSGTIPSGSQLQALDDQIYIYVGNPGLCGPPLLKNCSTNGTQQSFYEDRSHMRSLYLGMSIGFVIGLWTVFCTMMMKRTWMMAYFRIIDNLYDKAYVQVAISWSRLMRKNQDAA : 1128
S  NSFTV_66    : EIHLLIGLTNLNLSSNQFSGTIHDQIGDLKQLESLDLSYNELSGEIPPSLSALTSLSHLNLSYNNLSGTIPSGSQLQALDDQIYIYVGNPGLCGPPLLKNCSTNGTQQSFYEDRSHMRSLYLGMSIGFVIGLWTVFCTMMMKRTWMMAYFRIIDNLYDKAYVQVAISWSRLMRKNQDAA : 1128
S  NSFTV_110   : EIHLLIGLTNLNLSSNQFSGTIHDQIGDLKQLESLDLSYNELSGEIPPSLSALTSLSHLNLSYNNLSGTIPSGSQLQALDDQIYIYVGNPGLCGPPLLKNCSTNGTQQSFYEDRSHMRSLYLGMSIGFVIGLWTVFCTMMMKRTWMMAYFRIIDNLYDKAYVQVAISWSRLMRKNQDAA : 1129
S  NSFTV_138   : EIHLLIGLTNLNLSSNQFSGTIHDQIGDLKQLESLDLSYNELSGEIPPSLSALTSLSHLNLSYNNLSGTIPSGSQLQALDDQIYIYVGNPGLCGPPLLKNCSTNGTQQSFYEDRSHMRSLYLGMSIGFVIGLWTVFCTMMMKRTWMMAYFRIIDNLYDKAYVQVAISWSRLMRKNQDAA : 1129
S  NSFTV_145   : EIHLLIGLTNLNLSSNQFSGTIHDQIGDLKQLESLDLSYNELSGEIPPSLSALTSLSHLNLSYNNLSGTIPSGSQLQALDDQIYIYVGNPGLCGPPLLKNCSTNGTQQSFYEDRSHMRSLYLGMSIGFVIGLWTVFCTMMMKRTWMMAYFRIIDNLYDKAYVQVAISWSRLMRKNQDAA : 1128
S  NSFTV_252   : EIHLLIGLTNLNLSSNQFSGTIHDQIGDLKQLESLDLSYNELSGEIPPSLSALTSLSHLNLSYNNLSGTIPSGSQLQALDDQIYIYVGNPGLCGPPLLKNCSTNGTQQSFYEDRSHMRSLYLGMSIGFVIGLWTVFCTMMMKRTWMMAYFRIIDNLYDKAYVQVAISWSRLMRKNQDAA : 1129
S  NSFTV_255   : EIHLLIGLTNLNLSSNQFSGTIHDQIGDLKQLESLDLSYNELSGEIPPSLSALTSLSHLNLSYNNLSGTIPSGSQLQALDDQIYIYVGNPGLCGPPLLKNCSTNGTQQSFYEDRSHMRSLYLGMSIGFVIGLWTVFCTMMMKRTWMMAYFRIIDNLYDKAYVQVAISWSRLMRKNQDAA : 1129
S  NSFTV_304   : EIHLLIGLTNLNLSSNQFSGTIHDQIGDLKQLESLDLSYNELSGEIPPSLSALTSLSHLNLSYNNLSGTIPSGSQLQALDDQIYIYVGNPGLCGPPLLKNCSTNGTQQSFYEDRSHMRSLYLGMSIGFVIGLWTVFCTMMMKRTWMMAYFRIIDNLYDKAYVQVAISWSRLMRKNQDAA : 1128
                 EIHLLIGLTNLNLSSNQFSGTIHDQIGDLKQLESLDLSYNELSGEIPPSLSALTSLSHLNLSYNNLSGTIPSGSQLQALDDQIYIYVGNPGLCGPPLLKNCSTNGTQQSFYEDRSHM SLYLGMSIGFVIGLWTVFCTMMMKRTWMMAYFRIIDNLYDKAYVQVAISWSRLMRKNQDAA
```
