# Supplementary material for: Genome-wide association mapping of gene loci affecting disease resistance in the rice-Fusarium fujikuroi pathosystem
Source: Rice (N Y). 2019 Nov 21;12:85. doi: 10.1186/s12284-019-0337-3 (PMC6872702; doi:10.1186/s12284-019-0337-3)
Supplement: Supplementary file 8 — Additional file 8: Fig. S3. Alignment of the amino acid sequences of Os01g0601675. [file 12284_2019_337_MOESM8_ESM.pdf]

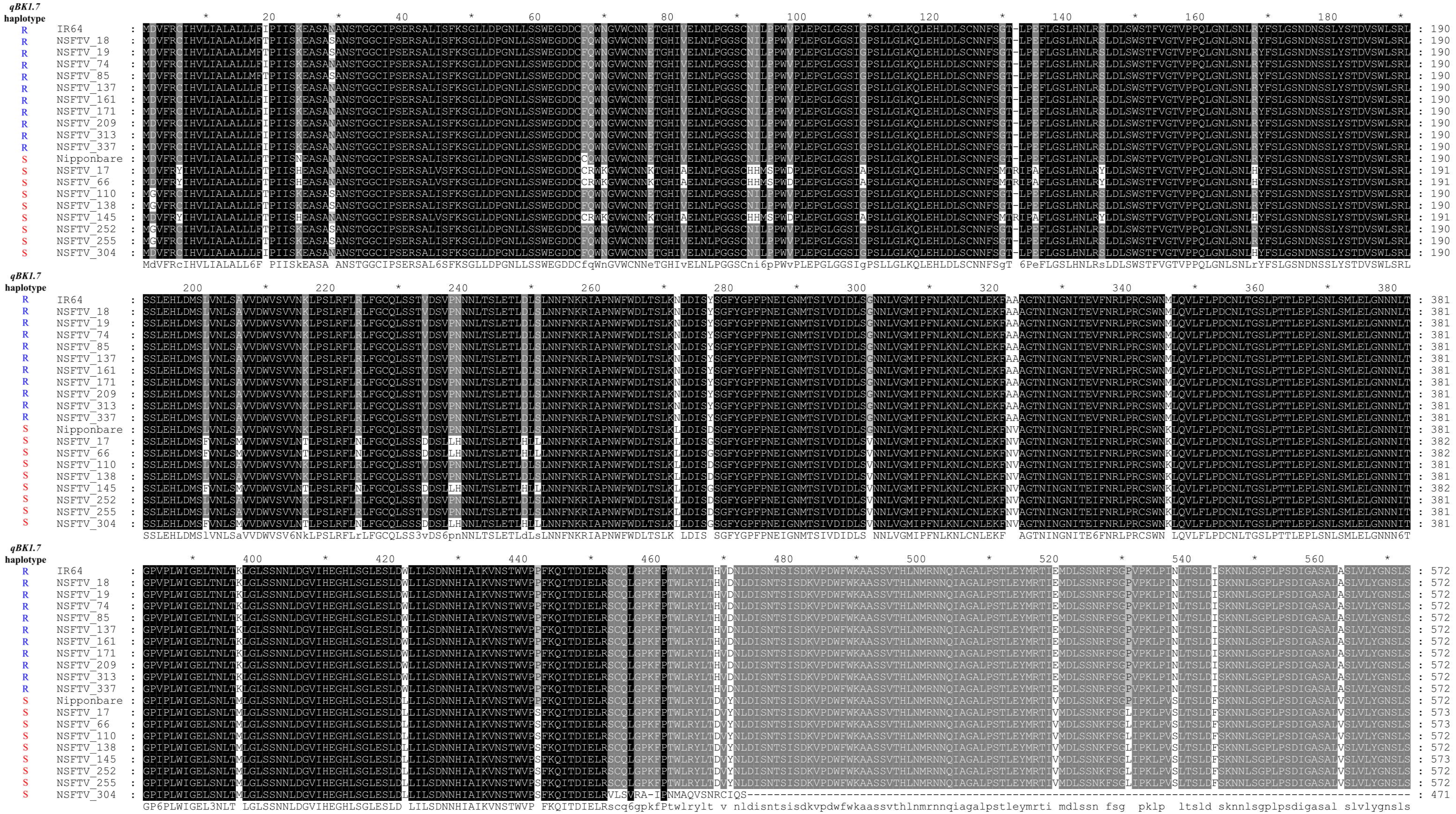
qBK1.7 haplotype
R IR64
R NSFTV_18
R NSFTV_19
R NSFTV_74
R NSFTV_85
R NSFTV_137
R NSFTV_161
R NSFTV_171
R NSFTV_209
R NSFTV_313
R NSFTV_337
S Nipponbare
S NSFTV_17
S NSFTV_66
S NSFTV_110
S NSFTV_138
S NSFTV_145
S NSFTV_252
S NSFTV_255
S NSFTV_304

```
qBK1.7
haplotype             580       *       600         *       620         *       640         *       660         *       680         *       700         *       720         *       740         *       760
R   IR64        : GSIPSYLCKMQSLELLDISRNKITGPLPDCAINSSSANSTCMNIINISLRNNNISGQFPSFFKNCKNLVFLDLAENQLSGTLPTWIGGKLPSLVFLRLRSNSFSGHIPIELTSLAGLQYLDLAHNNFSGCIPNSLAKFHRMTLEQDKEDRFSGAIRYGIGINDNDLVNYIENITVVTKGQERLYTGEIVYM : 763
R   NSFTV_18    : GSIPSYLCKMQSLELLDISRNKITGPLPDCAINSSSANSTCMNIINISLRNNNISGQFPSFFKNCKNLVFLDLAENQLSGTLPTWIGGKLPSLVFLRLRSNSFSGHIPIELTSLAGLQYLDLAHNNFSGCIPNSLAKFHRMTLEQDKEDRFSGAIRYGIGINDNDLVNYIENITVVTKGQERLYTGEIVYM : 763
R   NSFTV_19    : GSIPSYLCKMQSLELLDISRNKITGPLPDCAINSSSANSTCMNIINISLRNNNISGQFPSFFKNCKNLVFLDLAENQLSGTLPTWIGGKLPSLVFLRLRSNSFSGHIPIELTSLAGLQYLDLAHNNFSGCIPNSLAKFHRMTLEQDKEDRFSGAIRYGIGINDNDLVNYIENITVVTKGQERLYTGEIVYM : 763
R   NSFTV_74    : GSIPSYLCKMQSLELLDISRNKITGPLPDCAINSSSANSTCMNIINISLRNNNISGQFPSFFKNCKNLVFLDLAENQLSGTLPTWIGGKLPSLVFLRLRSNSFSGHIPIELTSLAGLQYLDLAHNNFSGCIPNSLAKFHRMTLEQDKEDRFSGAIRYGIGINDNDLVNYIENITVVTKGQERLYTGEIVYM : 763
R   NSFTV_85    : GSIPSYLCKMQSLELLDISRNKITGPLPDCAINSSSANSTCMNIINISLRNNNISGQFPSFFKNCKNLVFLDLAENQLSGTLPTWIGGKLPSLVFLRLRSNSFSGHIPIELTSLAGLQYLDLAHNNFSGCIPNSLAKFHRMTLEQDKEDRFSGAIRYGIGINDNDLVNYIENITVVTKGQERLYTGEIVYM : 763
R   NSFTV_137   : GSIPSYLCKMQSLELLDISRNKITGPLPDCAINSSSANSTCMNIINISLRNNNISGQFPSFFKNCKNLVFLDLAENQLSGTLPTWIGGKLPSLVFLRLRSNSFSGHIPIELTSLAGLQYLDLAHNNFSGCIPNSLAKFHRMTLEQDKEDRFSGAIRYGIGINDNDLVNYIENITVVTKGQERLYTGEIVYM : 763
R   NSFTV_161   : GSIPSYLCKMQSLELLDISRNKITGPLPDCAINSSSANSTCMNIINISLRNNNISGQFPSFFKNCKNLVFLDLAENQLSGTLPTWIGGKLPSLVFLRLRSNSFSGHIPIELTSLAGLQYLDLAHNNFSGCIPNSLAKFHRMTLEQDKEDRFSGAIRYGIGINDNDLVNYIENITVVTKGQERLYTGEIVYM : 763
R   NSFTV_171   : GSIPSYLCKMQSLELLDISRNKITGPLPDCAINSSSANSTCMNIINISLRNNNISGQFPSFFKNCKNLVFLDLAENQLSGTLPTWIGGKLPSLVFLRLRSNSFSGHIPIELTSLAGLQYLDLAHNNFSGCIPNSLAKFHRMTLEQDKEDRFSGAIRYGIGINDNDLVNYIENITVVTKGQERLYTGEIVYM : 763
R   NSFTV_209   : GSIPSYLCKMQSLELLDISRNKITGPLPDCAINSSSANSTCMNIINISLRNNNISGQFPSFFKNCKNLVFLDLAENQLSGTLPTWIGGKLPSLVFLRLRSNSFSGHIPIELTSLAGLQYLDLAHNNFSGCIPNSLAKFHRMTLEQDKEDRFSGAIRYGIGINDNDLVNYIENITVVTKGQERLYTGEIVYM : 763
R   NSFTV_313   : GSIPSYLCKMQSLELLDISRNKITGPLPDCAINSSSANSTCMNIINISLRNNNISGQFPSFFKNCKNLVFLDLAENQLSGTLPTWIGGKLPSLVFLRLRSNSFSGHIPIELTSLAGLQYLDLAHNNFSGCIPNSLAKFHRMTLEQDKEDRFSGAIRYGIGINDNDLVNYIENITVVTKGQERLYTGEIVYM : 763
R   NSFTV_337   : GSIPSYLCKMQSLELLDISRNKITGPLPDCAINSSSANSTCMNIINISLRNNNISGQFPSFFKNCKNLVFLDLAENQLSGTLPTWIGGKLPSLVFLRLRSNSFSGHIPIELTSLAGLQYLDLAHNNFSGCIPNSLAKFHRMTLEQDKEDRFSGAIRYGIGINDNDLVNYIENITVVTKGQERLYTGEIVYM : 763
S   Nipponbare  : GSIPSYLCKMQSLELLDISRNKITGPISDCAIDSSSANYTCTNIINISLRKNNLSGQFPSFFKNCKNLVFLDLAENQFSGTLPAWIGEKLPSLVFLRLRSNSFSGHIPIELTSLAGLQYLDLAHNNFSGCIPNSLAKFHRMTLEQDKEDRFSGAIRHGIGINDNDMVNYIENISVVTKGQERLYTGEIVYM : 763
S   NSFTV_17    : GSIPSYLCKMHSLELLDISRNKITGPISDCAIDSSSANYTCTNIINISLRKNNLSGQFPSFFKNCKNLVFLDLAENQFSGTLPEWIGEKLPSLVFLRLRSNSFSGHIPIELTSLAGLQIFRPCT------------------------------------------------------------------- : 697
S   NSFTV_66    : GSIPSYLCKMHSLELLDISRNKITGPISDCAIDSSSANYTCTNIINISLRKNNLSGQFPSFFKNCKNLVFLDLAENQFSGTLPEWIGEKLPSLVFLRLRSNSFSGHIPIELTSLAGLQIFRPCT------------------------------------------------------------------- : 697
S   NSFTV_110   : GSIPSYLCKMHSLELLDISRNKITGPISDCAIDSSSANYTCTNIINISLRKNNLSGQFPSFFKNCKNLVFLDLAENQFSGTLPEWIGEKLPSLVFLRLRSNSFSGHIPIELTSLAGLQIFRPCT-----QLFRMHTEFFGEIS-DDTRTR-RRQIFRGY-IWNWDQ------------------------- : 730
S   NSFTV_138   : GSIPSYLCKMHSLELLDISRNKITGPISDCAIDSSSANYTCTNIINISLRKNNLSGQFPSFFKNCKNLVFLDLAENQFSGTLPEWIGEKLPSLVFLRLRSNSFSGHIPIELTSLAGLQIFRPCT-----QLFRMHTEFFGEIS-DDTRTR-RRQIFRGY-IWNWDQ------------------------- : 730
S   NSFTV_145   : GSIPSYLCKMHSLELLDISRNKITGPISDCAIDSSSANYTCTNIINISLRKNNLSGQFPSFFKNCKNLVFLDLAENQFSGTLPEWIGEKLPSLVFLRLRSNSFSGHIPIELTSLAGLQIFRPCT------------------------------------------------------------------- : 697
S   NSFTV_252   : GSIPSYLCKMHSLELLDISRNKITGPISDCAIDSSSANYTCTNIINISLRKNNLSGQFPSFFKNCKNLVFLDLAENQFSGTLPEWIGEKLPSLVFLRLRSNSFSGHIPIELTSLAGLQIFRPCT------------------------------------------------------------------- : 696
S   NSFTV_255   : GSIPSYLCKMHSLELLDISRNKITGPISDCAIDSSSANYTCTNIINISLRKNNLSGQFPSFFKNCKNLVFLDLAENQFSGTLPEWIGEKLPSLVFLRLRSNSFSGHIPIELTSLAGLQIFRPCT------------------------------------------------------------------- : 696
S   NSFTV_304   : ------------------------------------------------------------------------------------------------------------------------------------------------------------------------------------------------ :   -
                  gsipsylckm slelldisrnkitgp  dcai sssan tc niinislr nn sgqfpsffkncknlvfldlaenq sgtlp wig klpslvflrlrsnsfsghipieltslaglq

qBK1.7
haplotype           *       780         *       800         *       820         *       840         *       860         *       880         *       900         *       920         *       940         *
R   IR64        : VNIDLSSNNLTGEIPEEIISLVALTNLNLSWNSLSGQIPEKIGSLSQLESLDLSHNVLSGGIPSSIASLTYLSHMNLSYNNLSGRIPAGNQLDILEDPASMYVGNIDLCGHPLPNNCSINGDTKIERDDLVNMSFHFSMIIGFMVGLLLVFYFMLFSRRWRNTCFVFVDGLYDRTYVQVAVTCRRLWRRN : 953
R   NSFTV_18    : VNIDLSSNNLTGEIPEEIISLVALTNLNLSWNSLSGQIPEKIGSLSQLESLDLSHNVLSGGIPSSIASLTYLSHMNLSYNNLSGRIPAGNQLDILEDPASMYVGNIDLCGHPLPNNCSINGDTKIERDDLVNMSFHFSMIIGFMVGLLLVFYFMLFSRRWRNTCFVFVDGLYDRTYVQVAVTCRRLWRRN : 953
R   NSFTV_19    : VNIDLSSNNLTGEIPEEIISLVALTNLNLSWNSLSGQIPEKIGSLSQLESLDLSHNVLSGGIPSSIASLTYLSHMNLSYNNLSGRIPAGNQLDILEDPASMYVGNIDLCGHPLPNNCSINGDTKIERDDLVNMSFHFSMIIGFMVGLLLVFYFMLFSRRWRNTCFVFVDGLYDRTYVQVAVTCRRLWRRN : 953
R   NSFTV_74    : VNIDLSSNNLTGEIPEEIISLVALTNLNLSWNSLSGQIPEKIGSLSQLESLDLSHNVLSGGIPSSIASLTYLSHMNLSYNNLSGRIPAGNQLDILEDPASMYVGNIDLCGHPLPNNCSINGDTKIERDDLVNMSFHFSMIIGFMVGLLLVFYFMLFSRRWRNTCFVFVDGLYDRTYVQVAVTCRRLWRRN : 953
R   NSFTV_85    : VNIDLSSNNLTGEIPEEIISLVALTNLNLSWNSLSGQIPEKIGSLSQLESLDLSHNVLSGGIPSSIASLTYLSHMNLSYNNLSGRIPAGNQLDILEDPASMYVGNIDLCGHPLPNNCSINGDTKIERDDLVNMSFHFSMIIGFMVGLLLVFYFMLFSRRWRNTCFVFVDGLYDRTYVQVAVTCRRLWRRN : 953
R   NSFTV_137   : VNIDLSSNNLTGEIPEEIISLVALTNLNLSWNSLSGQIPEKIGSLSQLESLDLSHNVLSGGIPSSIASLTYLSHMNLSYNNLSGRIPAGNQLDILEDPASMYVGNIDLCGHPLPNNCSINGDTKIERDDLVNMSFHFSMIIGFMVGLLLVFYFMLFSRRWRNTCFVFVDGLYDRTYVQVAVTCRRLWRRN : 953
R   NSFTV_161   : VNIDLSSNNLTGEIPEEIISLVALTNLNLSWNSLSGQIPEKIGSLSQLESLDLSHNVLSGGIPSSIASLTYLSHMNLSYNNLSGRIPAGNQLDILEDPASMYVGNIDLCGHPLPNNCSINGDTKIERDDLVNMSFHFSMIIGFMVGLLLVFYFMLFSRRWRNTCFVFVDGLYDRTYVQVAVTCRRLWRRN : 953
R   NSFTV_171   : VNIDLSSNNLTGEIPEEIISLVALTNLNLSWNSLSGQIPEKIGSLSQLESLDLSHNVLSGGIPSSIASLTYLSHMNLSYNNLSGRIPAGNQLDILEDPASMYVGNIDLCGHPLPNNCSINGDTKIERDDLVNMSFHFSMIIGFMVGLLLVFYFMLFSRRWRNTCFVFVDGLYDRTYVQVAVTCRRLWRRN : 953
R   NSFTV_209   : VNIDLSSNNLTGEIPEEIISLVALTNLNLSWNSLSGQIPEKIGSLSQLESLDLSHNVLSGGIPSSIASLTYLSHMNLSYNNLSGRIPAGNQLDILEDPASMYVGNIDLCGHPLPNNCSINGDTKIERDDLVNMSFHFSMIIGFMVGLLLVFYFMLFSRRWRNTCFVFVDGLYDRTYVQVAVTCRRLWRRN : 953
R   NSFTV_313   : VNIDLSSNNLTGEIPEEIISLVALTNLNLSWNSLSGQIPEKIGSLSQLESLDLSHNVLSGGIPSSIASLTYLSHMNLSYNNLSGRIPAGNQLDILEDPASMYVGNIDLCGHPLPNNCSINGDTKIERDDLVNMSFHFSMIIGFMVGLLLVFYFMLFSRRWRNTCFVFVDGLYDRTYVQVAVTCRRLWRRN : 953
R   NSFTV_337   : VNIDLSSNNLTGEIPEEIISLVALTNLNLSWNSLSGQIPEKIGSLSQLESLDLSHNVLSGGIPSSIASLTYLSHMNLSYNNLSGRIPAGNQLDILEDPASMYVGNIDLCGHPLPNNCSINGDTKIERDDLVNMSFHFSMIIGFMVGLLLVFYFMLFSRRWRNTCFVFVDGLYDRTYVQVAVTCRRLWRRN : 953
S   Nipponbare  : VNIDLSSNNLTGEIPEEIISLVALTNLNLSWNSLSGQIPEKIGSLSQLESLDLSHNVLSGGIPSSIASLTYLSHMNLSYNNLSGRIPAGNQLDILEDPASMYVGNIDLCGHPLPNNCSINGDTKIERDDLVNMSFHFSMIIGFMVGLLLVFYFMLFSRRWRNTCFVFVDGLYDRTYVQVAVTCRRLWRRN : 953
S   NSFTV_17    : ---------------------------------------------------------------------------------------------------------------------------------------------------------------------------------------------- :   -
S   NSFTV_66    : ---------------------------------------------------------------------------------------------------------------------------------------------------------------------------------------------- :   -
S   NSFTV_110   : ---------------------------------------------------------------------------------------------------------------------------------------------------------------------------------------------- :   -
S   NSFTV_138   : ---------------------------------------------------------------------------------------------------------------------------------------------------------------------------------------------- :   -
S   NSFTV_145   : ---------------------------------------------------------------------------------------------------------------------------------------------------------------------------------------------- :   -
S   NSFTV_252   : ---------------------------------------------------------------------------------------------------------------------------------------------------------------------------------------------- :   -
S   NSFTV_255   : ---------------------------------------------------------------------------------------------------------------------------------------------------------------------------------------------- :   -
S   NSFTV_304   : ---------------------------------------------------------------------------------------------------------------------------------------------------------------------------------------------- :   -
```
